# Supplementary material for: Competition for the conserved branch point sequence influences physiological outcomes in pre-mRNA splicing
Source: eLife. 2026 Mar 20;13:RP103167. doi: 10.7554/eLife.103167 (PMC13004596; doi:10.7554/eLife.103167)
Supplement: Figure 2—source data 1. — See text under each set of images for additional details. [file elife-103167-fig2-data1.pdf]

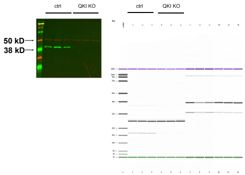

Figure 2—source data 1. Original western blot and capillary electrophoresis images for Figure 2A. Original uncropped images showing QKI protein levels and RAI14 ex 11 splicing patterns in control and QKI KO HEK293 cells. The left panel displays the original WB probed for Tubulin (red) and pan-QKI (green); the first lane corresponds to the Chameleon Duo molecular weight ladder with sizes indicated in kilodaltons (kDa). The right panel displays the original BioAnalyzer gel-like image for RAI14 ex 11. Lane L corresponds to the molecular weight ladder with sizes indicated in base pairs (bp). Lanes 1–6 correspond to the biological triplicates of endogenous RAI14 splicing in controll and QKI KO HEK293 cells as depicted in the main manuscript. Lanes 7–12 correspond to biological triplicates of control and QKI KO HEK293 cells transfected with the RAI14 ex 11 reporter construct; these lanes were not depicted in the final version of Figure 2A.

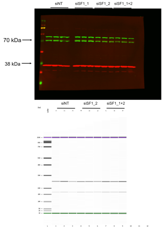

Figure 2—source data 2. Original western blot and capillary electrophoresis images for Figure 2C. Original uncropped images showing SF1 protein levels and RAI14 ex 11 splicing patterns following siRNA knockdown in HEK293 cells. The top panel displays the original WB probed for SF1 (green) and Gapdh (red); The first lane corresponds to the Chameleon Duo molecular weight ladder with sizes indicated in kilodaltons (kDa). Following the ladder, the blot includes biological triplicates for siNT, for siSF1_1 (not depicted in the final version of Figure 2C), siSF1_2 and siSF1_1+2. The bottom panel displays the original BioAnalyzer gel-like image for RAI14 ex 11. Lane L corresponds to the molecular weight ladder with sizes indicated in base pairs (bp). Lanes 1–9 correspond to the biological triplicates of siNT, siSF1_2, and siSF1_1+2 RT-PCR samples as depicted in the main manuscript.

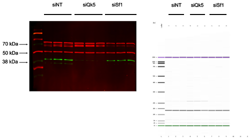

Figure 2—source data 3. Original western blot and capillary electrophoresis images for Figure 2D. Original uncropped images showing SF1 and QKI protein levels and RAI14 ex 11 splicing patterns following siRNA knockdown in C2C12 myoblasts. The left panel displays the original WB probed for SF1 (red), Tubulin (red, 50 kDa), and pan-QKI (green); the first lane corresponds to the Chameleon Duo molecular weight ladder with sizes indicated in kilodaltons (kDa). Following the ladder, the blot includes

Figure 2, Source Data 1. Original membranes and bioanalyzer gel-like images corresponding to Figure 2A (left), Figure 2C (middle), and Fig.ure 2D (right). See text under each for additional details
