# Supplementary figures and images for: Competition for the conserved branch point sequence influences physiological outcomes in pre-mRNA splicing
Source: eLife. 2026 Mar 20;13:RP103167. doi: 10.7554/eLife.103167 (PMC13004596; doi:10.7554/eLife.103167)

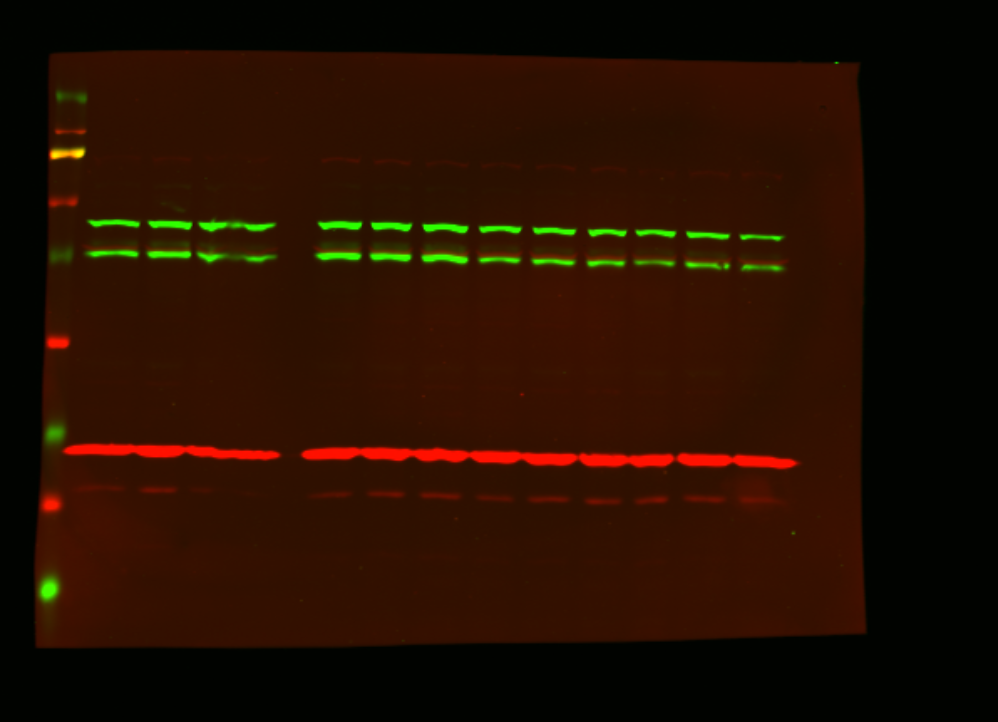

Supplement: Figure 2—source data 2. [file elife-103167-fig2-data2.zip › Fig2/1_18_23_HEKsiRNAsf1wb.png]

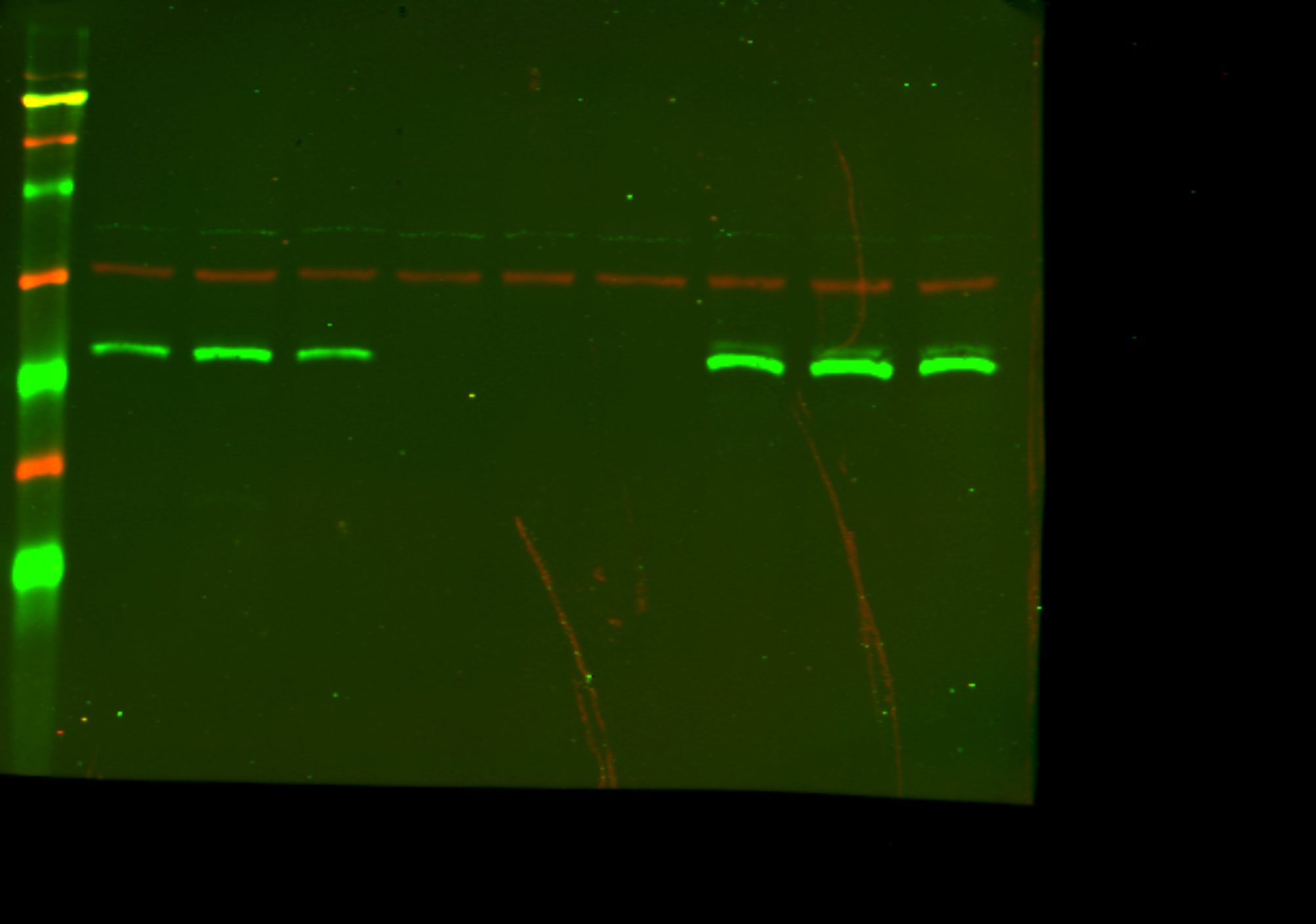

Supplement: Figure 2—source data 2. [file elife-103167-fig2-data2.zip › Fig2/hek_wt_ko.tif]

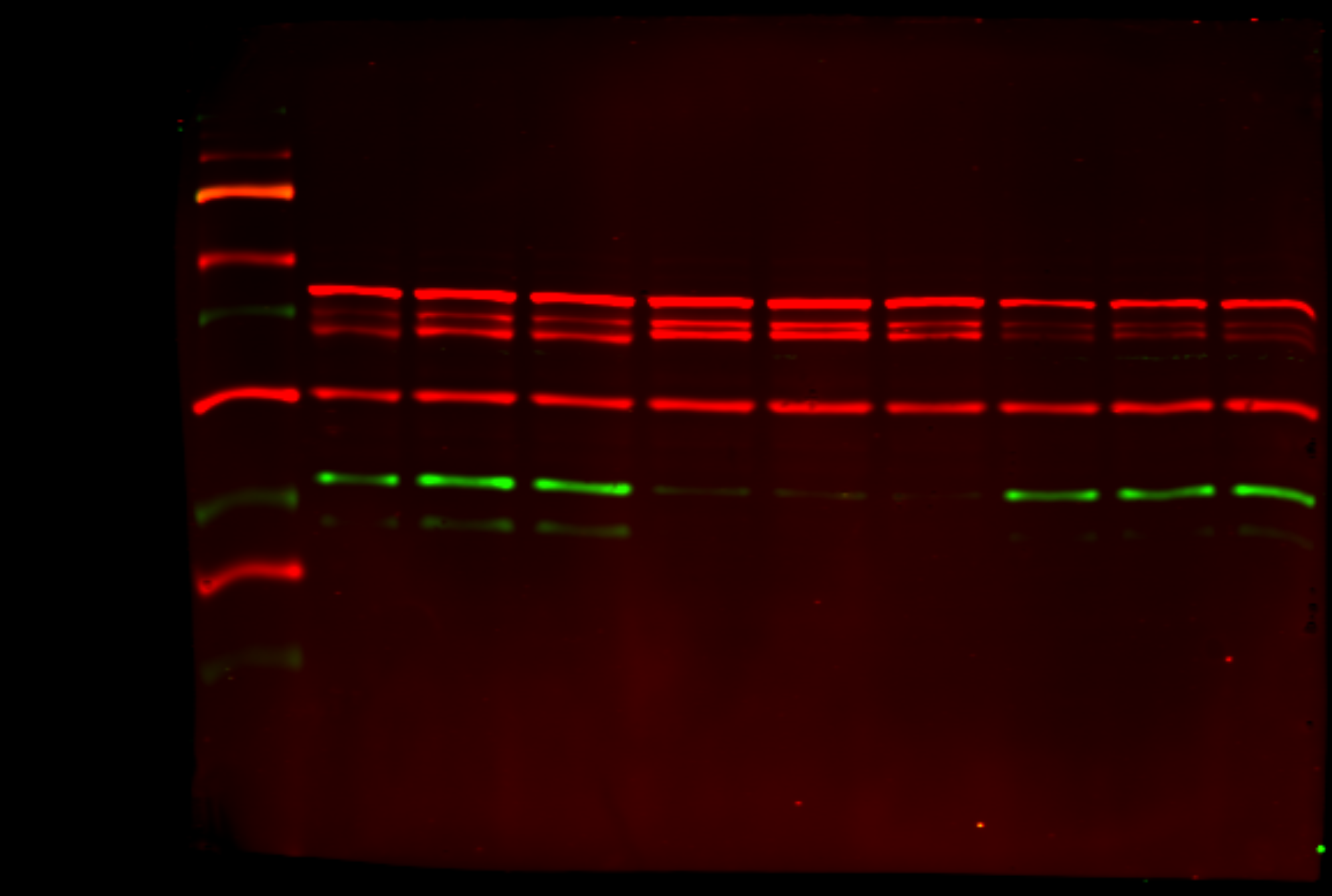

Supplement: Figure 2—source data 2. [file elife-103167-fig2-data2.zip › Fig2/3_12_24_c2c12_siNT_siQKI_siSF1_qki_tubulin_sf1.tif]

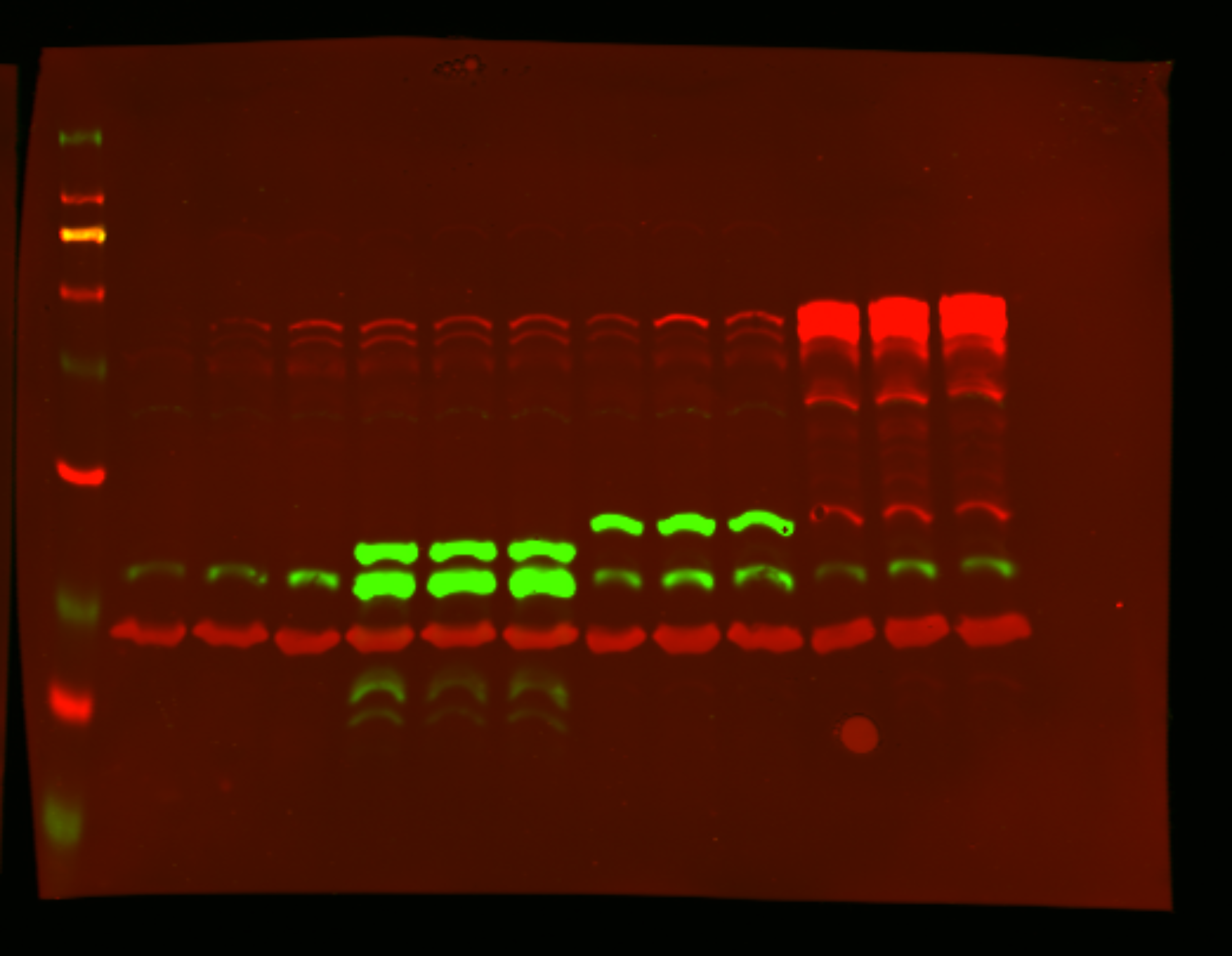

Supplement: Figure 2—figure supplement 1—source data 2. [file elife-103167-fig2-figsupp1-data2.zip › SupplFigS2/2_10_23_hek_wt_mt_sf1.tif]

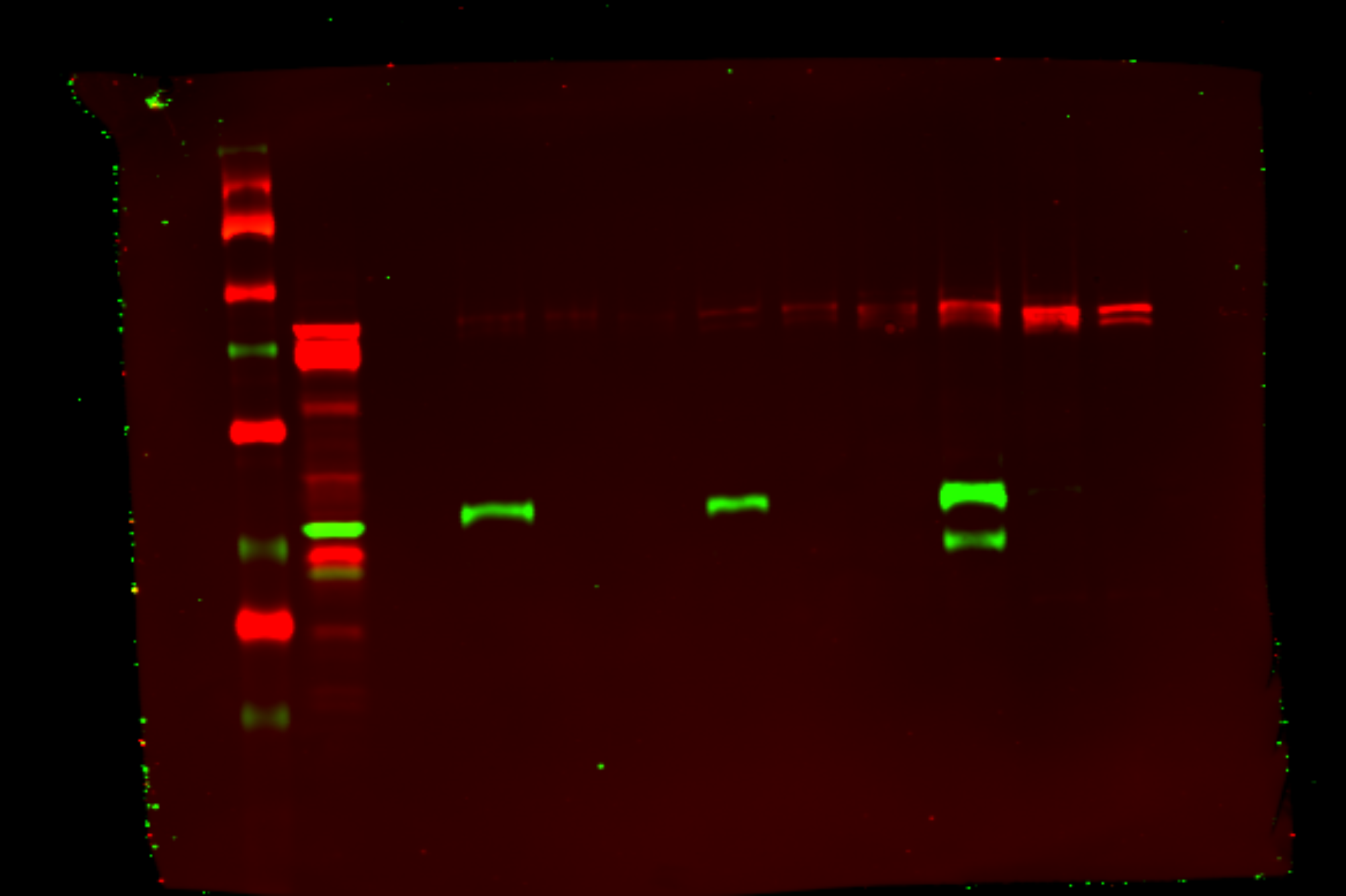

Supplement: Figure 4—source data 2. [file elife-103167-fig4-data2.zip › Fig4/4_25_24_tobra_noatp_7.5_15_30min_SF1_qki.tif]

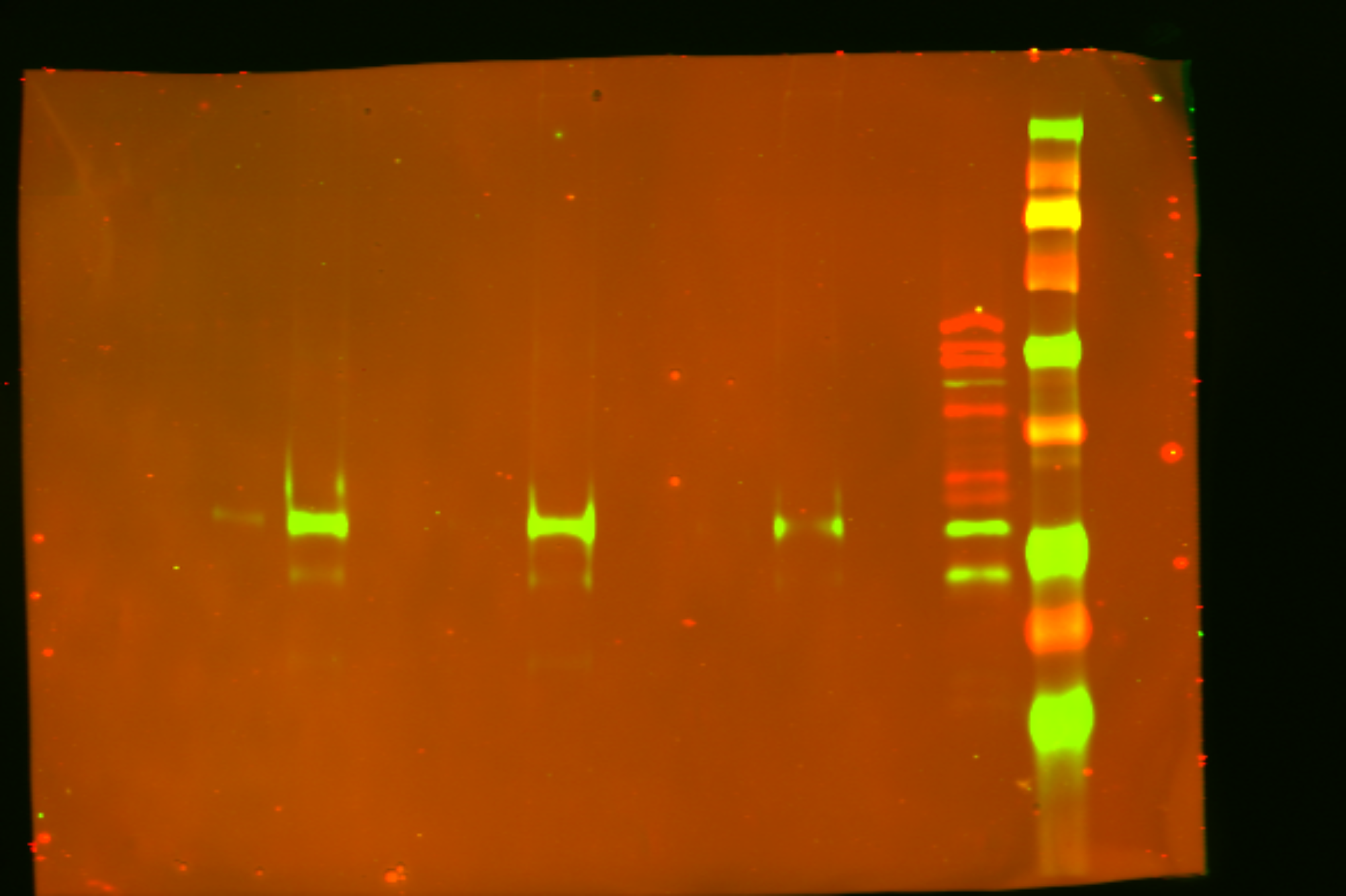

Supplement: Figure 4—source data 2. [file elife-103167-fig4-data2.zip › Fig4/040524-Rai14-RAC-withATP_WB-Sf1-Qki-elution_color.tif]

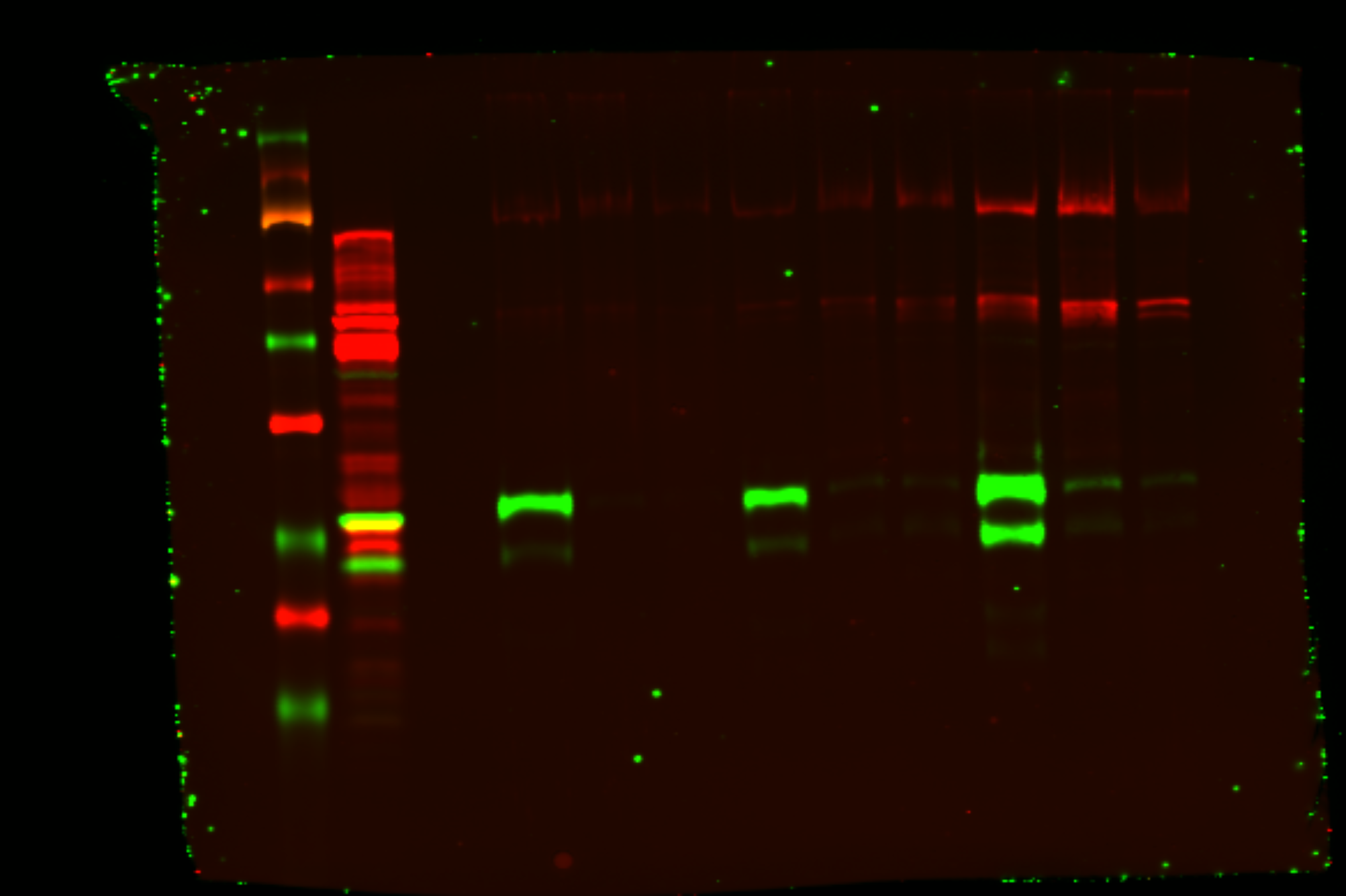

Supplement: Figure 4—source data 2. [file elife-103167-fig4-data2.zip › Fig4/4_26_24_tobra_noatp_7.5_15_30min_SF1_qki_htatsf1.tif]

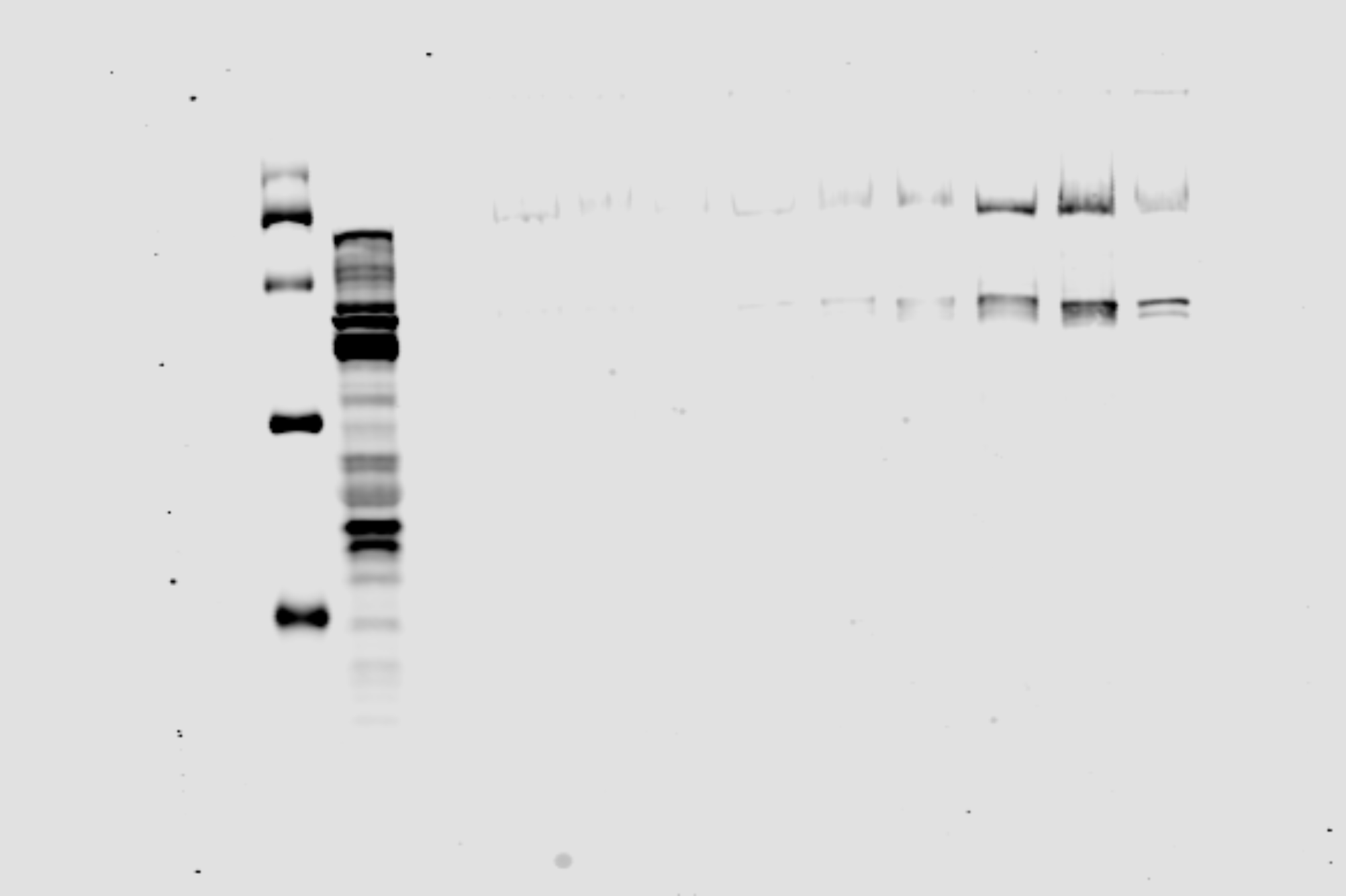

Supplement: Figure 4—source data 2. [file elife-103167-fig4-data2.zip › Fig4/042524-Rai14-RAC-noATP_WB-Sf1-Tatsf1-flowthrough_grayscale.tif]

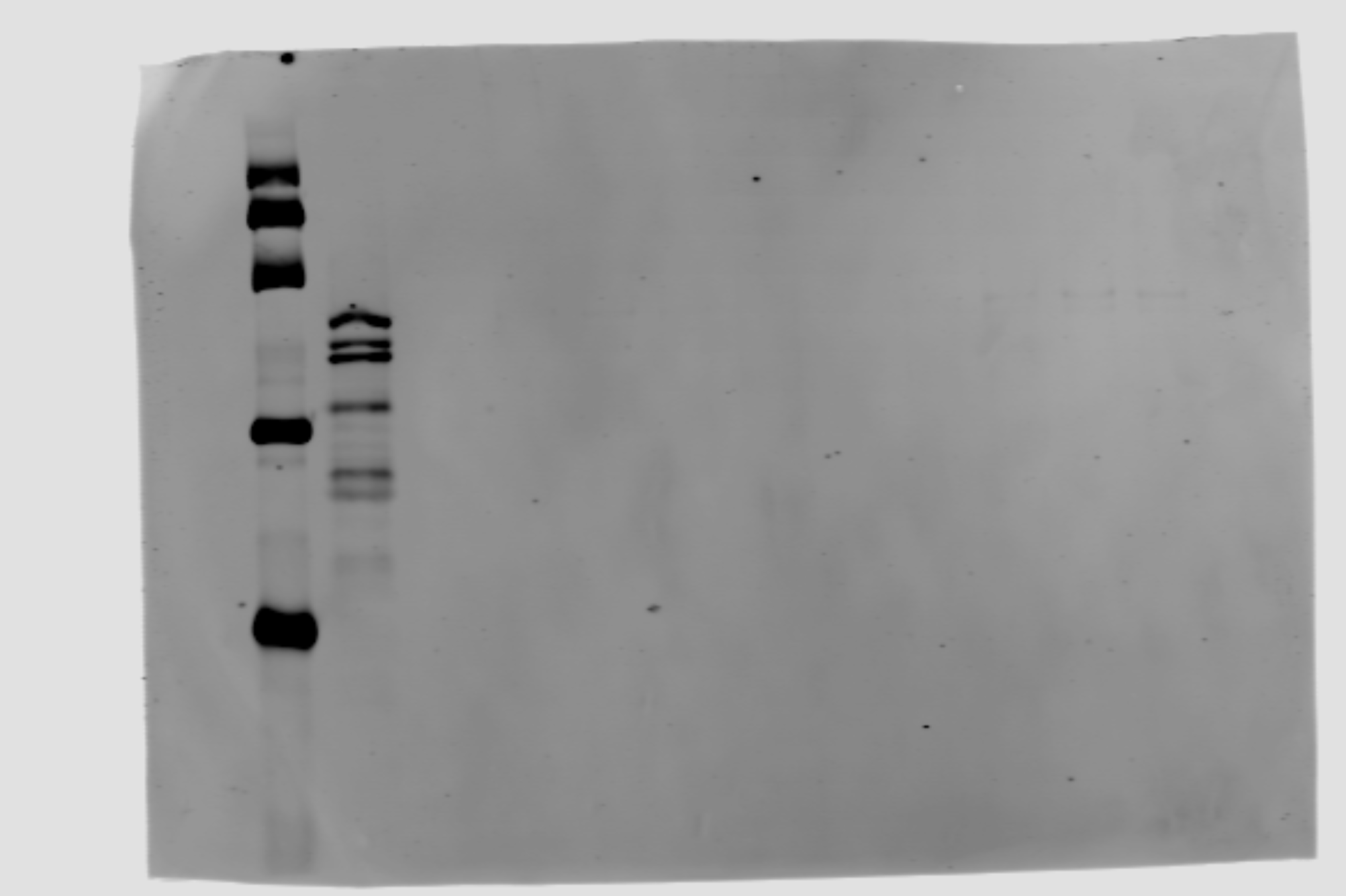

Supplement: Figure 4—source data 2. [file elife-103167-fig4-data2.zip › Fig4/040524-Rai14-RAC-withATP_WB-Sf1-elution_grayscale.tif]

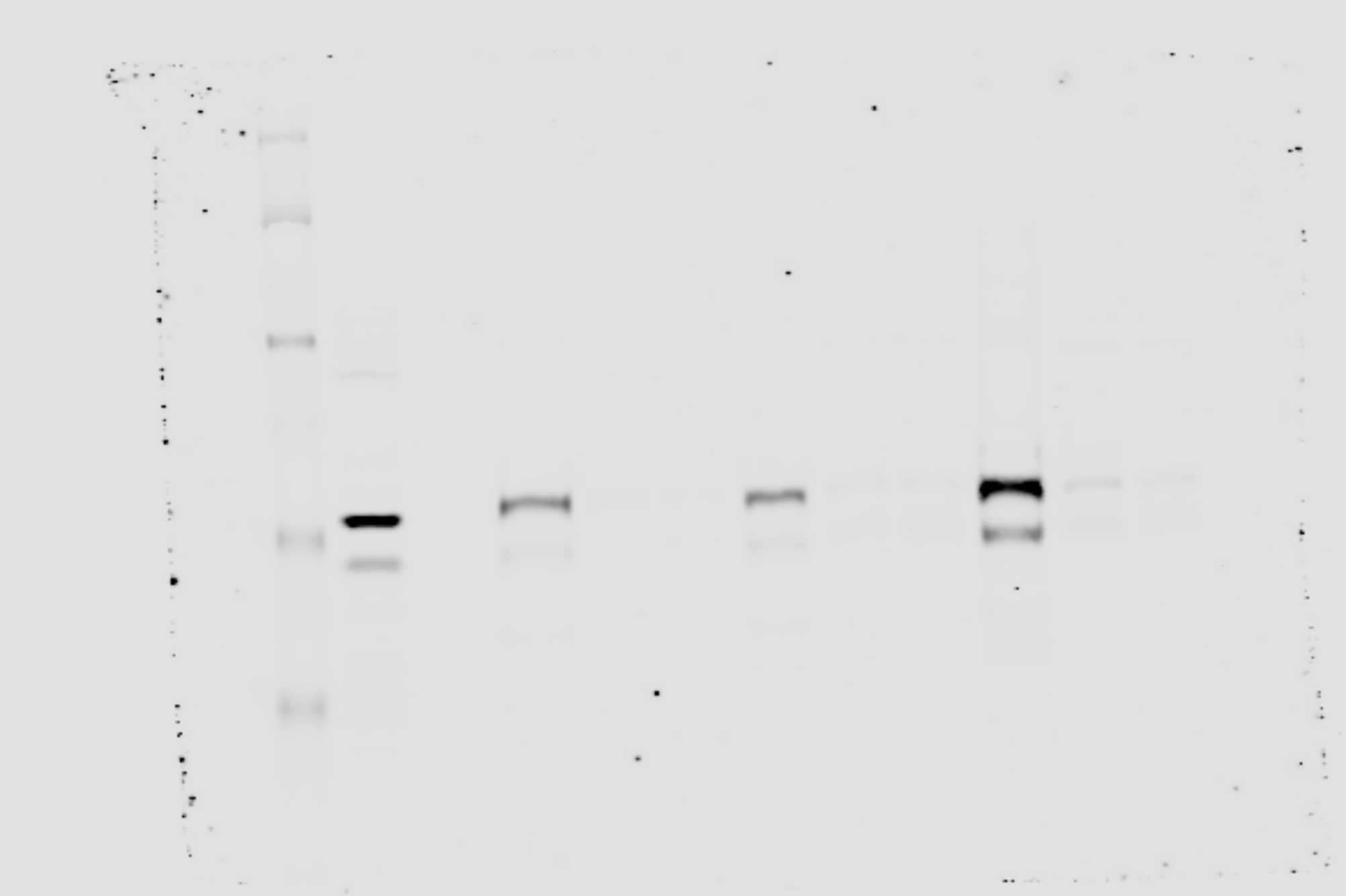

Supplement: Figure 4—source data 2. [file elife-103167-fig4-data2.zip › Fig4/042624-Rai14-RAC-noATP_WB-Qki-elution_grayscale.tif]

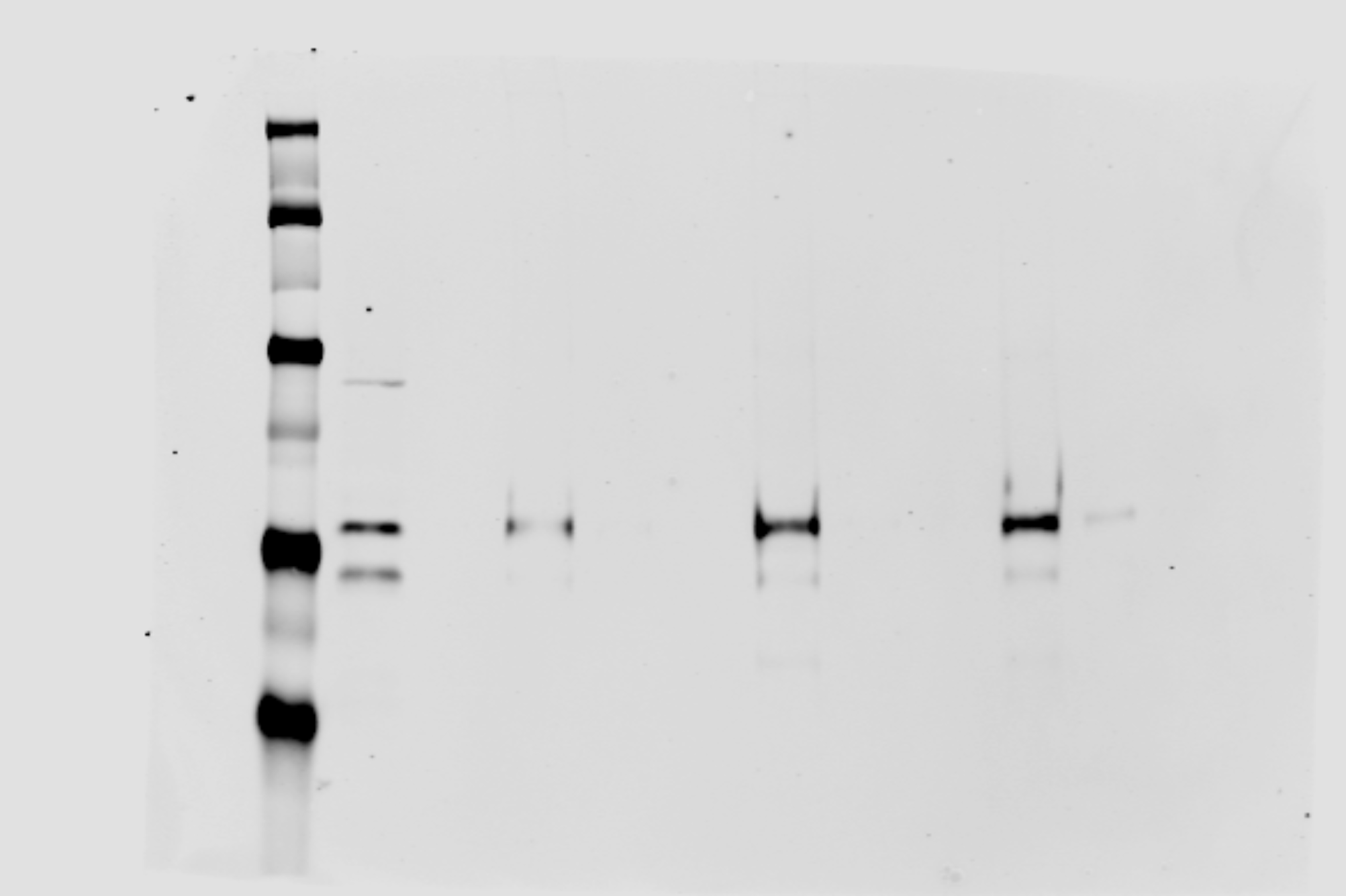

Supplement: Figure 4—source data 2. [file elife-103167-fig4-data2.zip › Fig4/040524-Rai14-RAC-withATP_WB-Qki-elution_grayscale.tif]

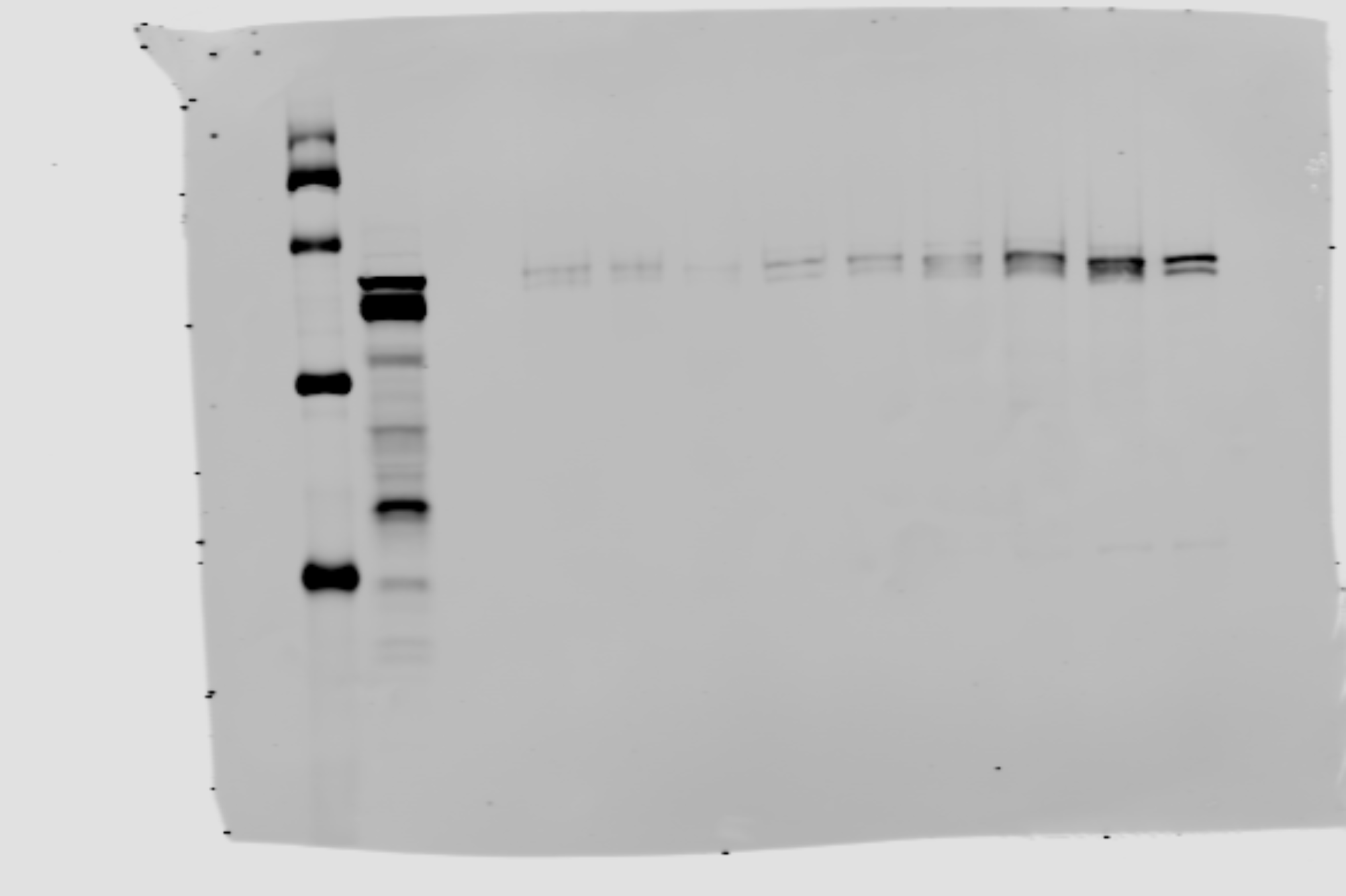

Supplement: Figure 4—source data 2. [file elife-103167-fig4-data2.zip › Fig4/042524-Rai14-RAC-noATP_WB-Sf1i-elution_grayscale.tif]

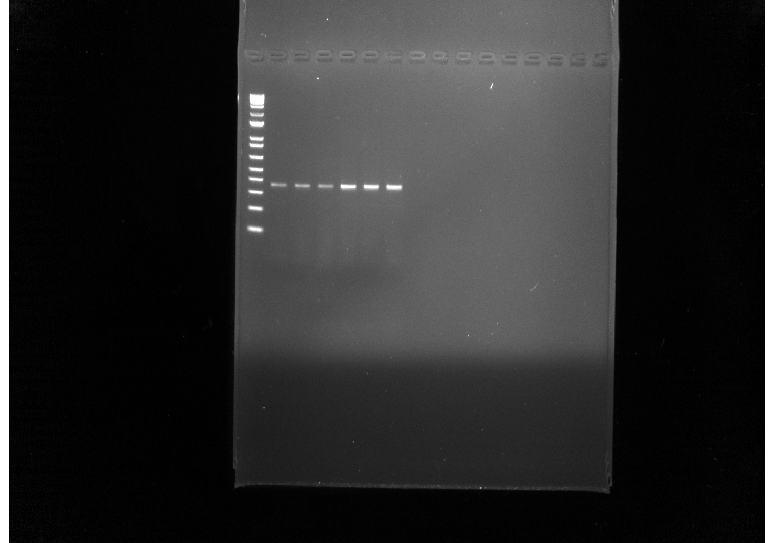

Supplement: Figure 5—figure supplement 1—source data 2. [file elife-103167-fig5-figsupp1-data2.zip › SupplFigS5/2023-12-19_VPS29_362.tif]

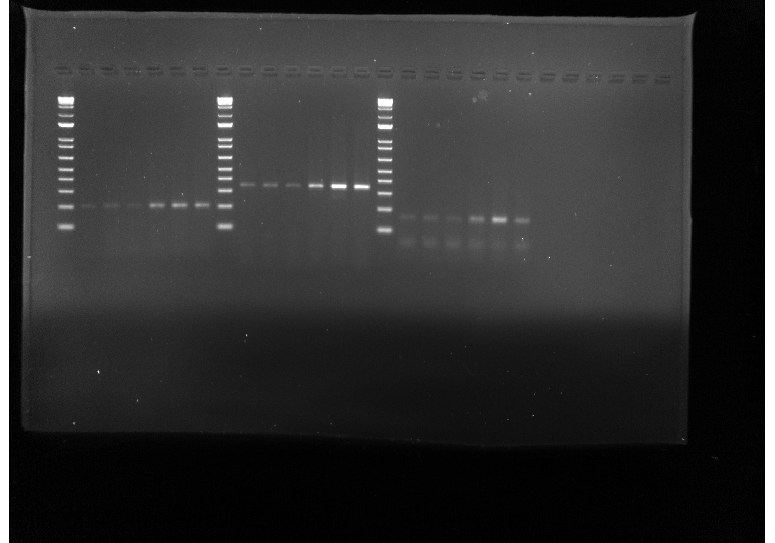

Supplement: Figure 5—figure supplement 1—source data 2. [file elife-103167-fig5-figsupp1-data2.zip › SupplFigS5/2023-12-19_RPL7Bintron1_203_POP8_341_YOS1_160.tif]

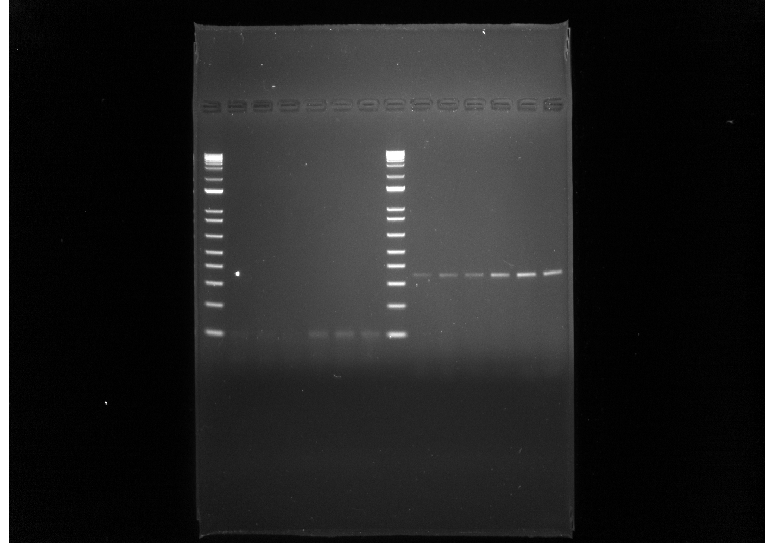

Supplement: Figure 5—figure supplement 1—source data 2. [file elife-103167-fig5-figsupp1-data2.zip › SupplFigS5/2023-12-22_1SUS1_100_RPS25A_362.tif]

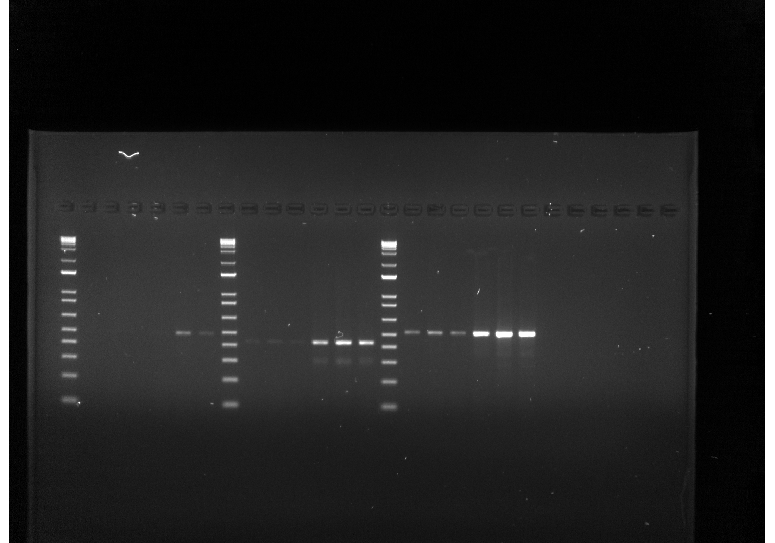

Supplement: Figure 5—figure supplement 1—source data 2. [file elife-103167-fig5-figsupp1-data2.zip › SupplFigS5/2023-12-15_RPL17B_490_RPL30_430_RPL28_522.tif]

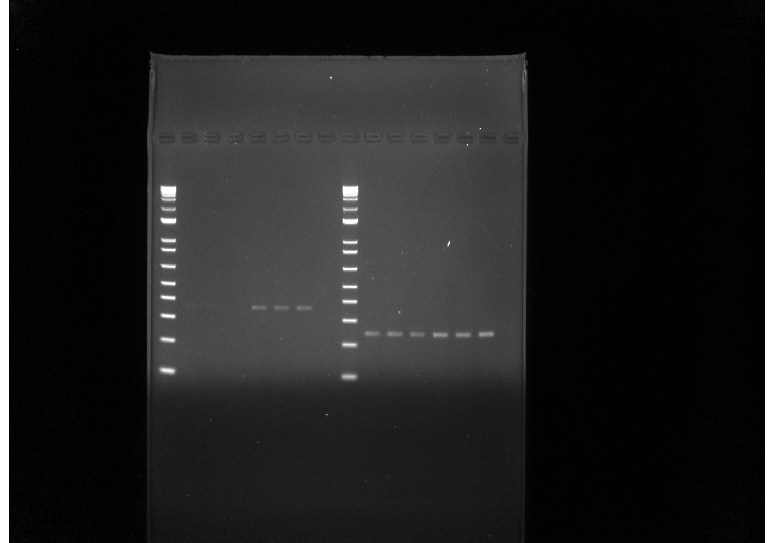

Supplement: Figure 5—figure supplement 1—source data 2. [file elife-103167-fig5-figsupp1-data2.zip › SupplFigS5/2024-01-02__OM14_359_hac1_244.tif]

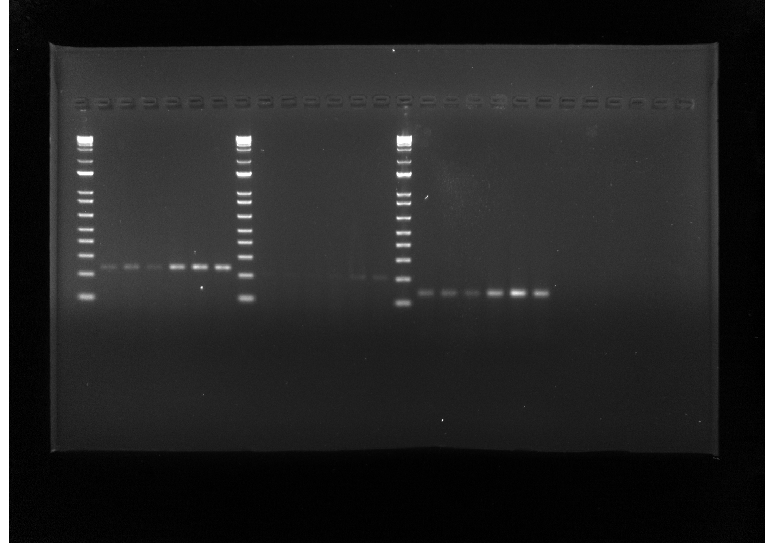

Supplement: Figure 5—figure supplement 1—source data 2. [file elife-103167-fig5-figsupp1-data2.zip › SupplFigS5/2023-12-18_GOT1_236_RPL7Bintron1_203_DB639731_135.tif]

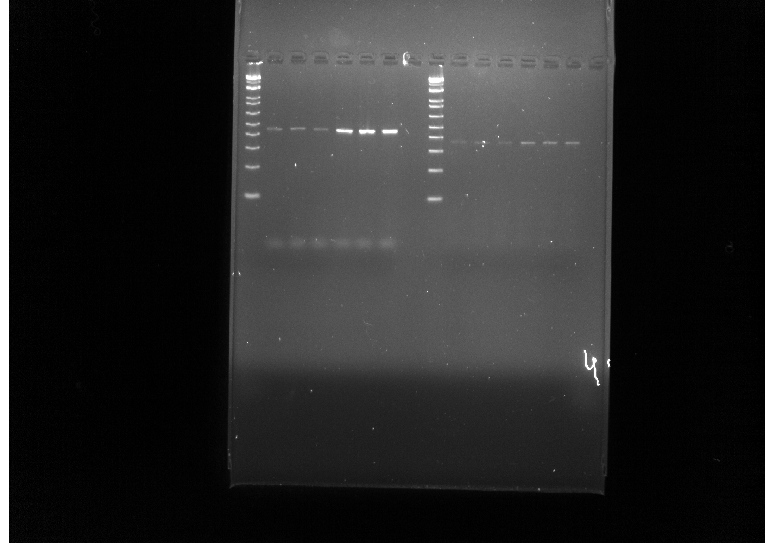

Supplement: Figure 5—figure supplement 1—source data 2. [file elife-103167-fig5-figsupp1-data2.zip › SupplFigS5/2023-12-19_TMA20_443_vps29_362.tif]

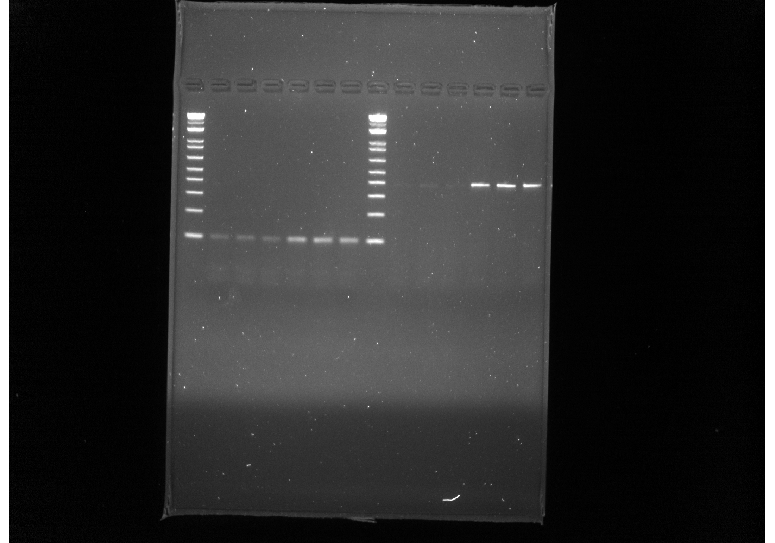

Supplement: Figure 5—figure supplement 1—source data 2. [file elife-103167-fig5-figsupp1-data2.zip › SupplFigS5/2023-12-22_sus1_100_RPL17B_374(differentprimerset).tif]

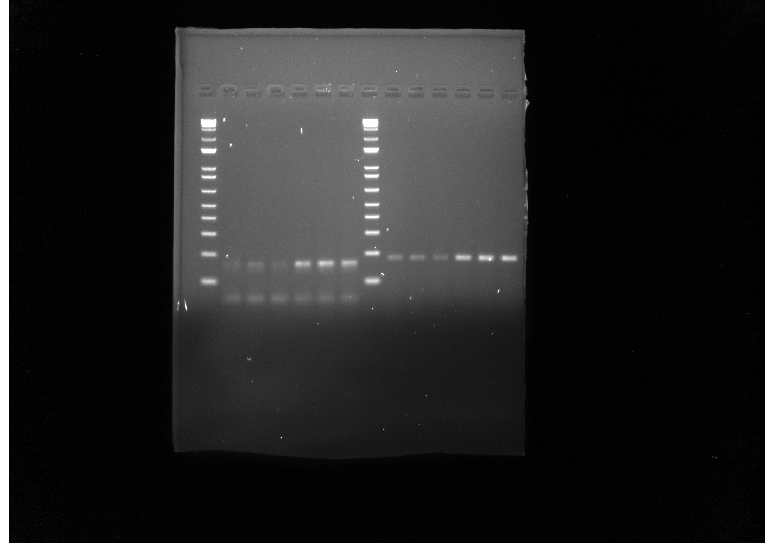

Supplement: Figure 5—figure supplement 1—source data 2. [file elife-103167-fig5-figsupp1-data2.zip › SupplFigS5/2023-12-19_YOS1_160_YPR170W_179.tif]
