# Supplementary material for: Competition for the conserved branch point sequence influences physiological outcomes in pre-mRNA splicing
Source: eLife. 2026 Mar 20;13:RP103167. doi: 10.7554/eLife.103167 (PMC13004596; doi:10.7554/eLife.103167)
Supplement: Figure 2—figure supplement 1—source data 1. [file elife-103167-fig2-figsupp1-data1.pdf]

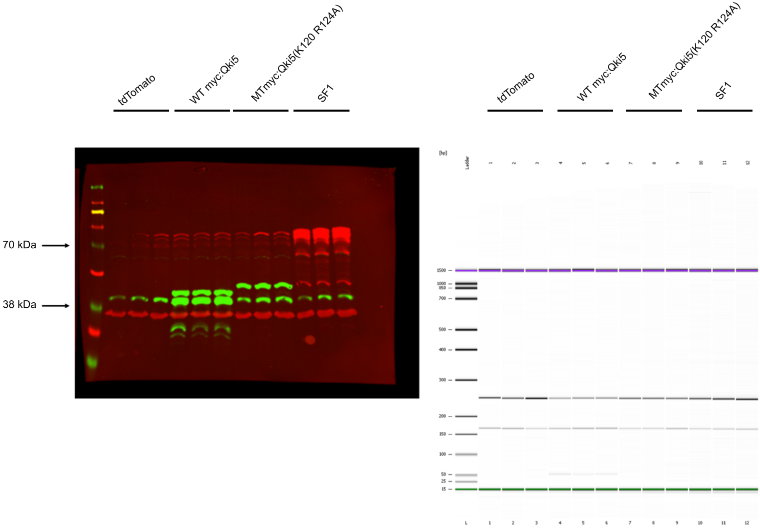

Supplementary Figure 2—source data 1. Original western blot and capillary electrophoresis images for Supplementary Figure 2. Original uncropped images showing SF1, QKI, and GAPDH protein levels and RAI14 ex 11 splicing patterns in HEK293 cells. The left panel displays the original WB probed for SF1 (red), pan-QKI (green), and GAPDH (red); the first lane corresponds to the Chameleon Duo molecular weight ladder with sizes indicated in kilodaltons (kDa). Following the ladder, the blot in cludes biological triplicates for cells transfected with tdTomato, WT myc:-Qki5, MT myc:Qki5 and SF1 expression vectors as depicted in the main manuscript. The right panel displays the original BioAnalyzer gel-like image for RAI14 ex 11. Lane L corresponds to the molecular weight ladder with sizes indicated in base pairs (bp). Lanes 1–12 correspond to the biological triplicates of tdTomato, WT myc:Qki5, MT myc:Qki5, and SF1 RT-PCR samples as depicted in the main manuscript.
