# Supplementary material for: Competition for the conserved branch point sequence influences physiological outcomes in pre-mRNA splicing
Source: eLife. 2026 Mar 20;13:RP103167. doi: 10.7554/eLife.103167 (PMC13004596; doi:10.7554/eLife.103167)

Assay Class:	DNA 1000	Created:	4/26/2023 1:07:52 PM
Data Path:	C:\...-26\2100 expert_DNA 1000_DE13804763_2023-04-26_13-07-53.xad	Modified:	4/26/2023 1:50:45 PM

Electrophoresis File Run Summary

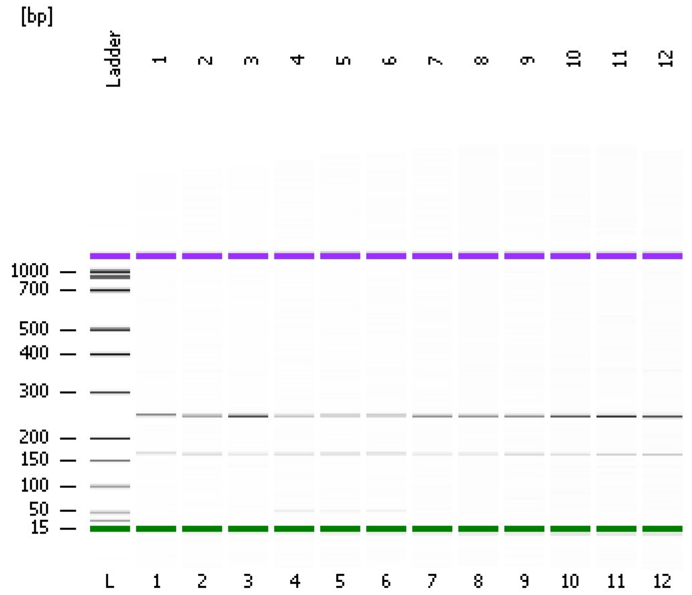

Instrument Information:

Instrument Name:	DE13804763	Firmware:	C.01.069
Serial#:	DE13804763	Type:	G2939A

Assay Information:

Assay Origin Path:	C:\Program Files\Agilent\2100 bioanalyzer\2100 expert\assays\dsDNA\DNA 1000 Series II.xsy
Assay Class:	DNA 1000
Version:	2.3
Assay Comments:	DNA Analysis 25 -1000 bp
	© Copyright 2003-2009 Agilent Technologies, Inc.

Chip Information:

Chip Lot #:

Reagent Kit Lot #:

Chip Comments:

1

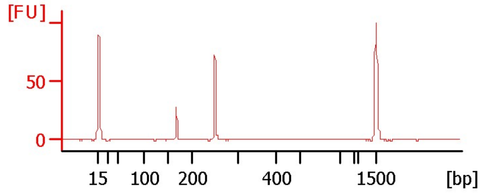

2

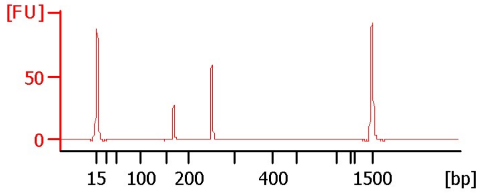

3

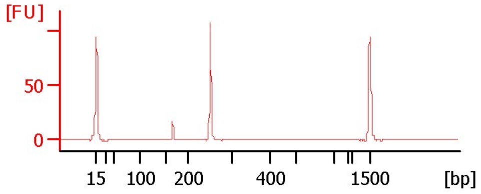

4

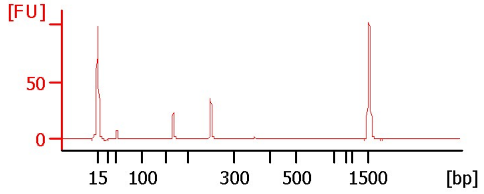

5

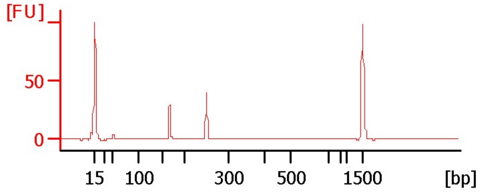

6

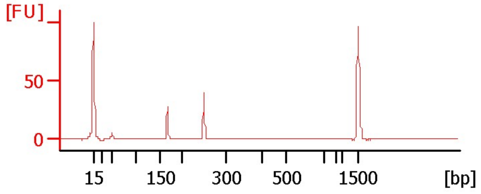

7

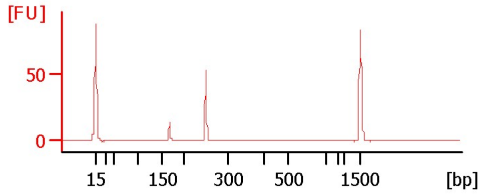

8

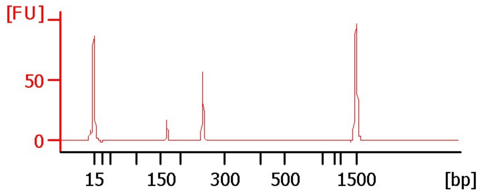

9

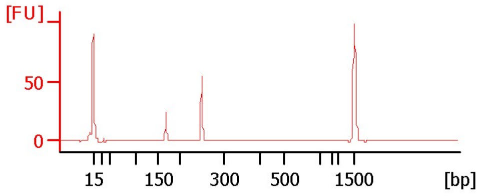

10

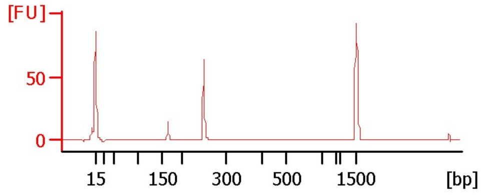

11

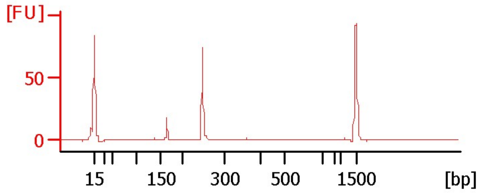

12

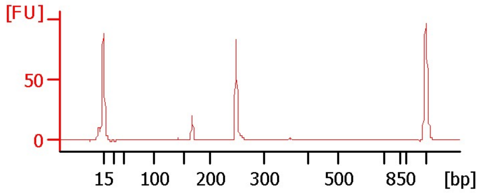

Assay Class: DNA 1000
Data Path: C:\...-26\2100 expert_DNA 1000_DE13804763_2023-04-26_13-07-53.xad
Created: 4/26/2023 1:07:52 PM
Modified: 4/26/2023 1:50:45 PM

Electrophoresis File Run Summary (Chip Summary)

Sample Name	Sample Comment	Rest. Digest	Status	Observation	Result Label	Result Color
1		☐	✔			
2		☐	✔			
3		☐	✔			
4		☐	✔			
5		☐	✔			
6		☐	✔			
7		☐	✔			
8		☐	✔			
9		☐	✔			
10		☐	✔			
11		☐	✔			
12		☐	✔			
Ladder		☐	✔			

Chip Lot # **Reagent Kit Lot #**

Chip Comments :

© Copyright 2003 - 2009 Agilent Technologies, Inc.

Assay Class: DNA 1000 Created: 4/26/2023 1:07:52 PM
Data Path: C:\...-26\2100 expert_DNA 1000_DE13804763_2023-04-26_13-07-53.xad Modified: 4/26/2023 1:50:45 PM

Electrophoresis Assay Details

General Analysis Settings

Number of Available Sample and Ladder Wells (Max.) : 13
Minimum Visible Range [s] : 30
Maximum Visible Range [s] : 129
Start Analysis Time Range [s] : 30
End Analysis Time Range [s] : 128.95
Ladder Concentration [ng/µl] : 44
Uses Standard Area for Ladder Fragments
Lower Marker Concentration [ng/µl] : 4.2
Upper Marker Concentration [ng/µl] : 2.1
Used Upper Marker for Quantitation
Standard Curve Fit is Point to Point
Show Data Aligned to Lower and Upper Marker

Integrator Settings

Integration Start Time [s] : 30
Integration End Time [s] : 128.95
Slope Threshold : 0.5
Height Threshold [FU] : 1
Area Threshold : 0.1
Width Threshold [s] : 0.5
Baseline Plateau [s] : 0.5

Filter Settings

Filter Width [s] : 0.5
Polynomial Order : 4

Ladder

Ladder Peak	Size	Area
1	15	25
2	25	26
3	50	34
4	100	41
5	150	45
6	200	52
7	300	63
8	400	76
9	500	83
10	700	88
11	850	86
12	1000	90
13	1500	52

 © Copyright 2003 - 2009 Agilent Technologies, Inc.

Assay Class: DNA 1000
Data Path: C:\...-26\2100 expert_DNA 1000_DE13804763_2023-04-26_13-07-53.xad

Created: 4/26/2023 1:07:52 PM
Modified: 4/26/2023 1:50:45 PM

Electropherogram Summary

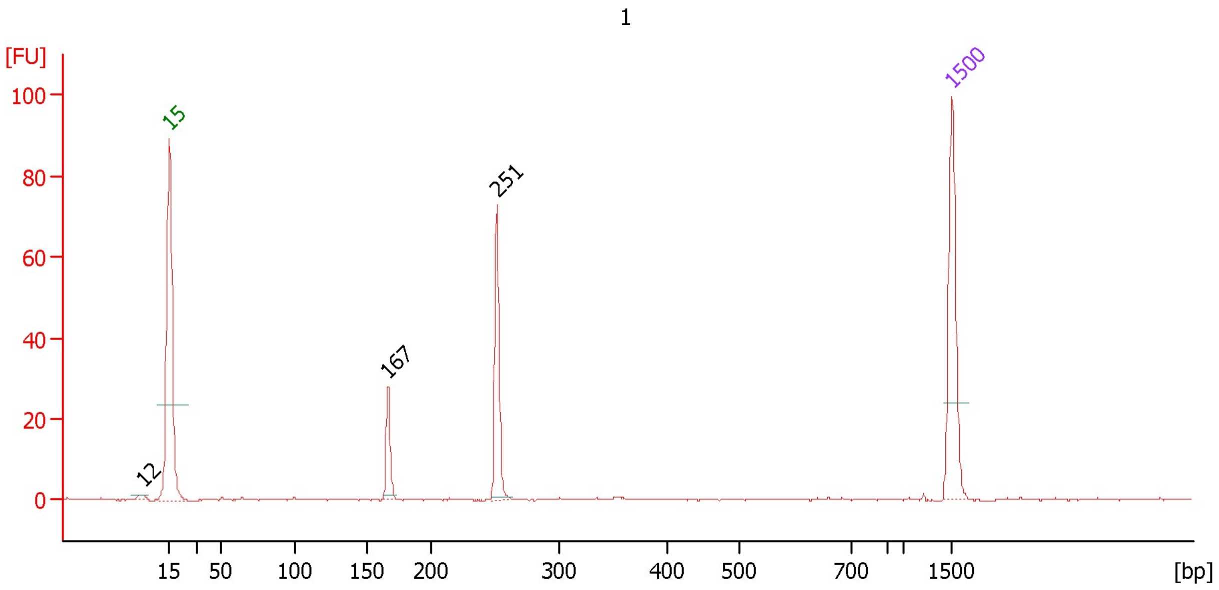

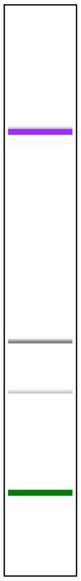

Overall Results for sample 1 : 1

Number of peaks found: 2

Peak table for sample 1 : 1

Peak		Size [bp]	Conc. [ng/µl]	Molarity [nmol/l]	Observations
1		12	0.00	0.0	
2	◀	15	4.20	424.2	Lower Marker
3		167	0.70	6.3	
4		251	1.63	9.8	
5	▶	1,500	2.10	2.1	Upper Marker

 © Copyright 2003 - 2009 Agilent Technologies, Inc.

Assay Class: DNA 1000
Data Path: C:\...-26\2100 expert_DNA 1000_DE13804763_2023-04-26_13-07-53.xad

Created: 4/26/2023 1:07:52 PM
Modified: 4/26/2023 1:50:45 PM

Electropherogram Summary Continued ...

2

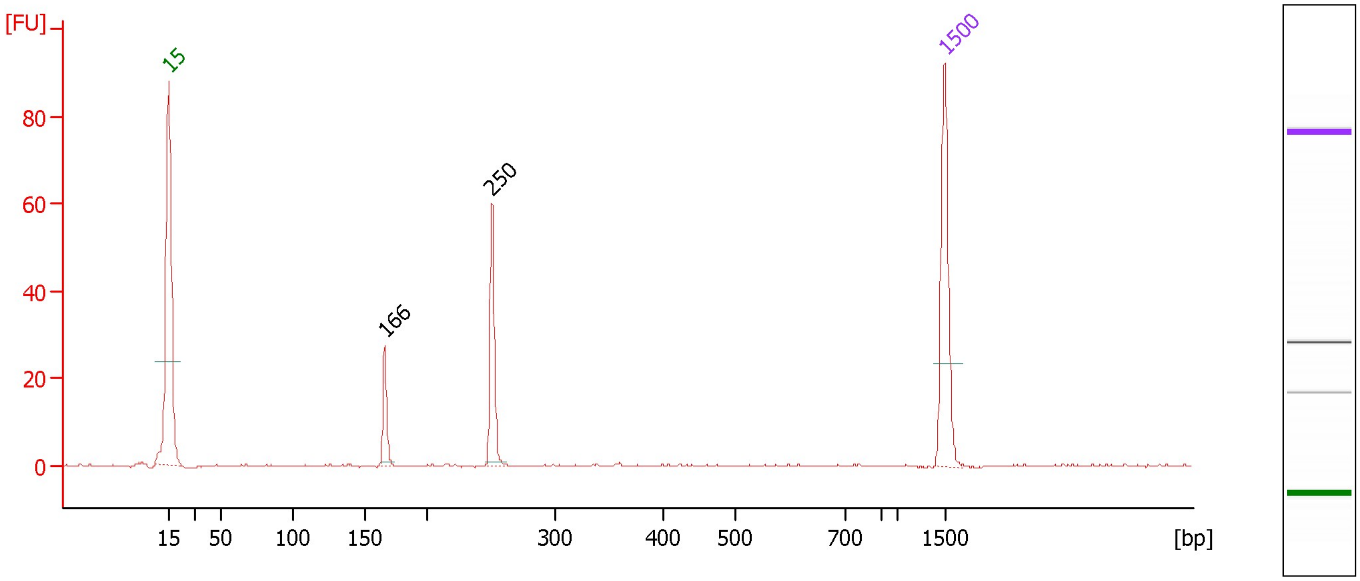

Overall Results for sample 2 : 2

Number of peaks found: 2

Peak table for sample 2 : 2

Peak		Size [bp]	Conc. [ng/µl]	Molarity [nmol/l]	Observations
1	◀	15	4.20	424.2	Lower Marker
2		166	0.71	6.5	
3		250	1.44	8.7	
4	▶	1,500	2.10	2.1	Upper Marker

 © Copyright 2003 - 2009 Agilent Technologies, Inc.

Assay Class: DNA 1000
Data Path: C:\...-26\2100 expert_DNA 1000_DE13804763_2023-04-26_13-07-53.xad
Created: 4/26/2023 1:07:52 PM
Modified: 4/26/2023 1:50:45 PM

Electropherogram Summary Continued ...

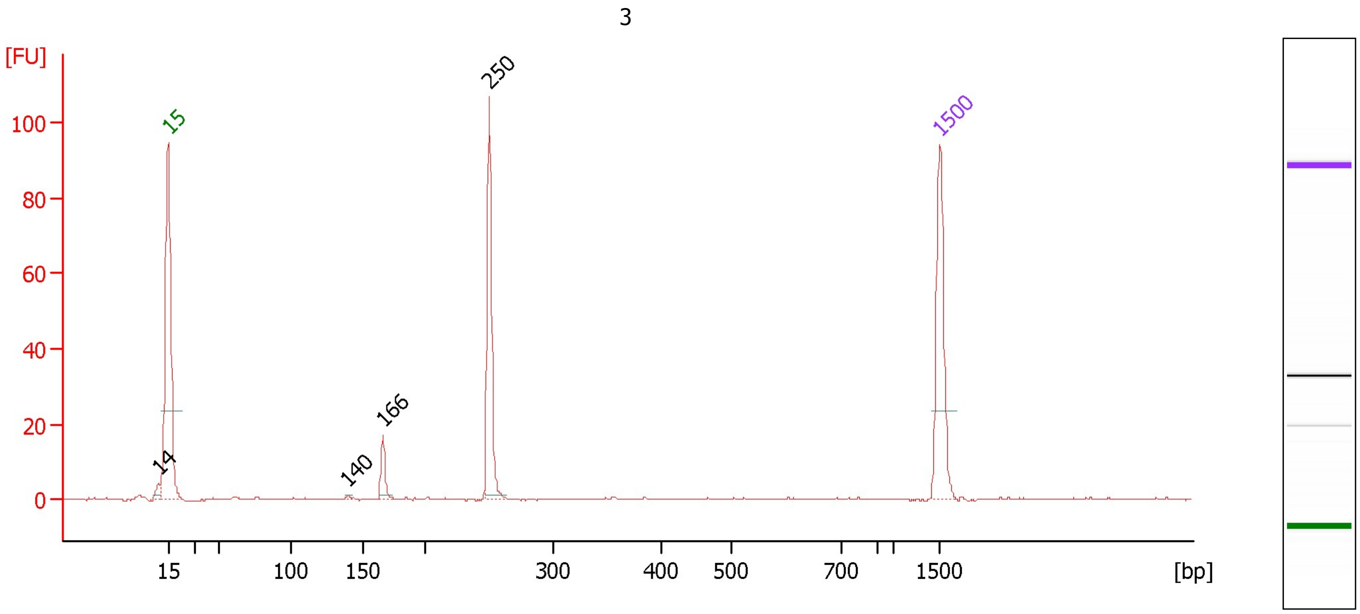

Overall Results for sample 3 : **3**

Number of peaks found: 3

Peak table for sample 3 : **3**

**Peak**		**Size [bp]**	**Conc. [ng/µl]**	**Molarity [nmol/l]**	**Observations**
1		14	0.00	0.0	
2	◀	15	4.20	424.2	Lower Marker
3		140	0.02	0.3	
4		166	0.44	4.0	
5		250	2.47	15.0	
6	▶	1,500	2.10	2.1	Upper Marker

 © Copyright 2003 - 2009 Agilent Technologies, Inc.

Assay Class:	DNA 1000	Created:	4/26/2023 1:07:52 PM
Data Path:	C:\...-26\2100 expert_DNA 1000_DE13804763_2023-04-26_13-07-53.xad	Modified:	4/26/2023 1:50:45 PM

Electropherogram Summary Continued ...

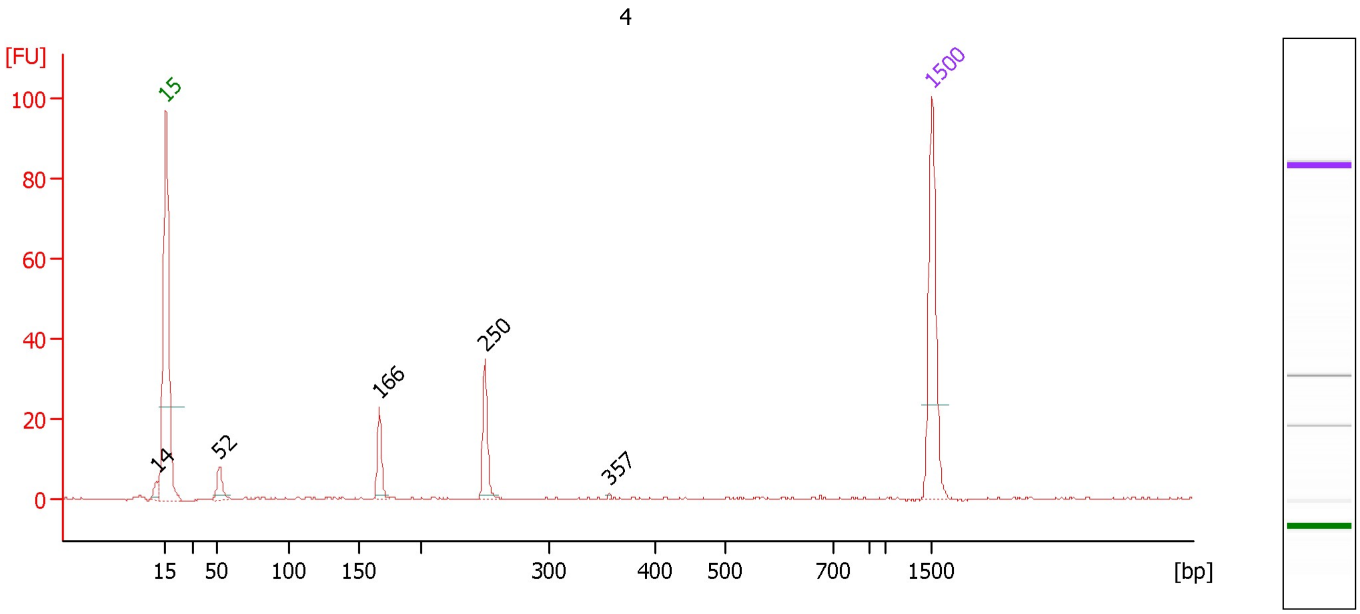

Overall Results for sample 4 : 4

Number of peaks found: 4

Peak table for sample 4 : 4

Peak		Size [bp]	Conc. [ng/µl]	Molarity [nmol/l]	Observations
1		14	0.00	0.0	
2	◀	15	4.20	424.2	Lower Marker
3		52	0.39	11.3	
4		166	0.57	5.2	
5		250	0.79	4.8	
6		357	0.01	0.1	
7	▶	1,500	2.10	2.1	Upper Marker

 © Copyright 2003 - 2009 Agilent Technologies, Inc.

Assay Class: DNA 1000
Data Path: C:\...-26\2100 expert_DNA 1000_DE13804763_2023-04-26_13-07-53.xad

Created: 4/26/2023 1:07:52 PM
Modified: 4/26/2023 1:50:45 PM

Electropherogram Summary Continued ...

5

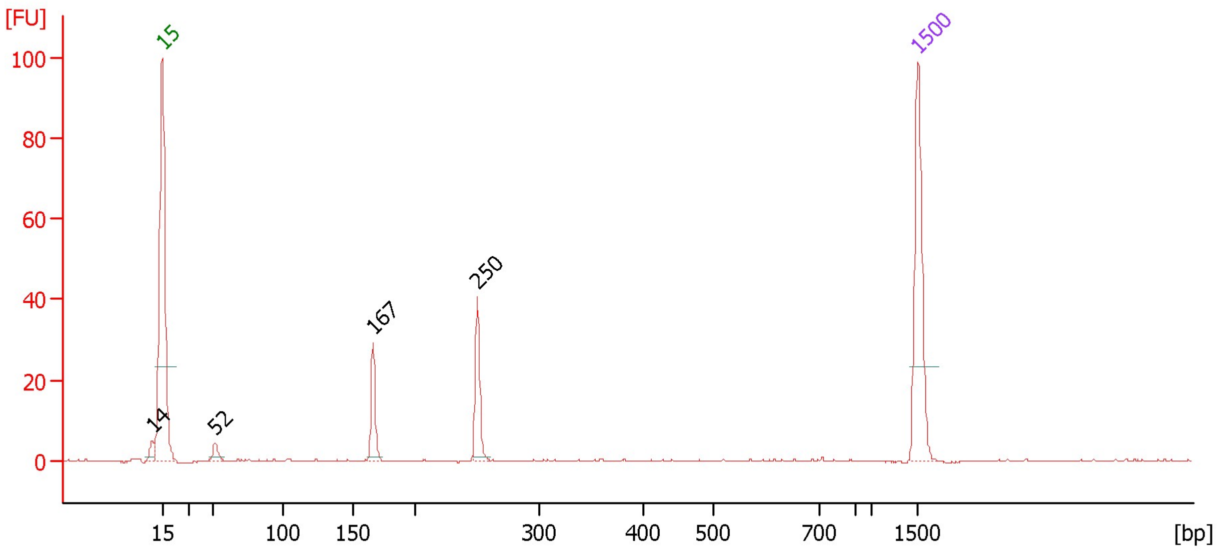

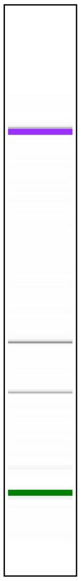

Overall Results for sample 5 : 5

Number of peaks found: 3

Peak table for sample 5 : 5

Peak		Size [bp]	Conc. [ng/µl]	Molarity [nmol/l]	Observations
1		14	0.00	0.0	
2	◀	15	4.20	424.2	Lower Marker
3		52	0.21	6.2	
4		167	0.72	6.6	
5		250	0.91	5.5	
6	▶	1,500	2.10	2.1	Upper Marker

 © Copyright 2003 - 2009 Agilent Technologies, Inc.

Assay Class: DNA 1000
Data Path: C:\...-26\2100 expert_DNA 1000_DE13804763_2023-04-26_13-07-53.xad
Created: 4/26/2023 1:07:52 PM
Modified: 4/26/2023 1:50:45 PM

Electropherogram Summary Continued ...

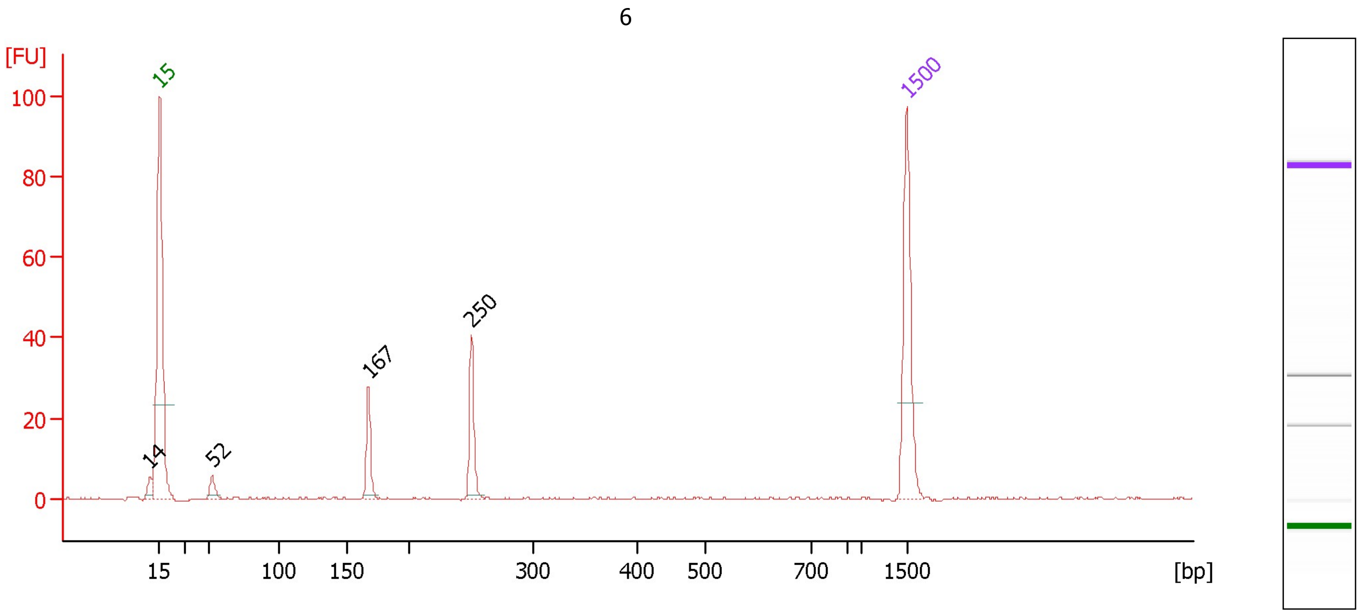

Overall Results for sample 6 : 6

Number of peaks found: 3

Peak table for sample 6 : 6

Peak		Size [bp]	Conc. [ng/µl]	Molarity [nmol/l]	Observations
1		14	0.00	0.0	
2	◀	15	4.20	424.2	Lower Marker
3		52	0.26	7.6	
4		167	0.72	6.5	
5		250	0.94	5.7	
6	▶	1,500	2.10	2.1	Upper Marker

 © Copyright 2003 - 2009 Agilent Technologies, Inc.

Assay Class: DNA 1000
Data Path: C:\...-26\2100 expert_DNA 1000_DE13804763_2023-04-26_13-07-53.xad

Created: 4/26/2023 1:07:52 PM
Modified: 4/26/2023 1:50:45 PM

Electropherogram Summary Continued ...

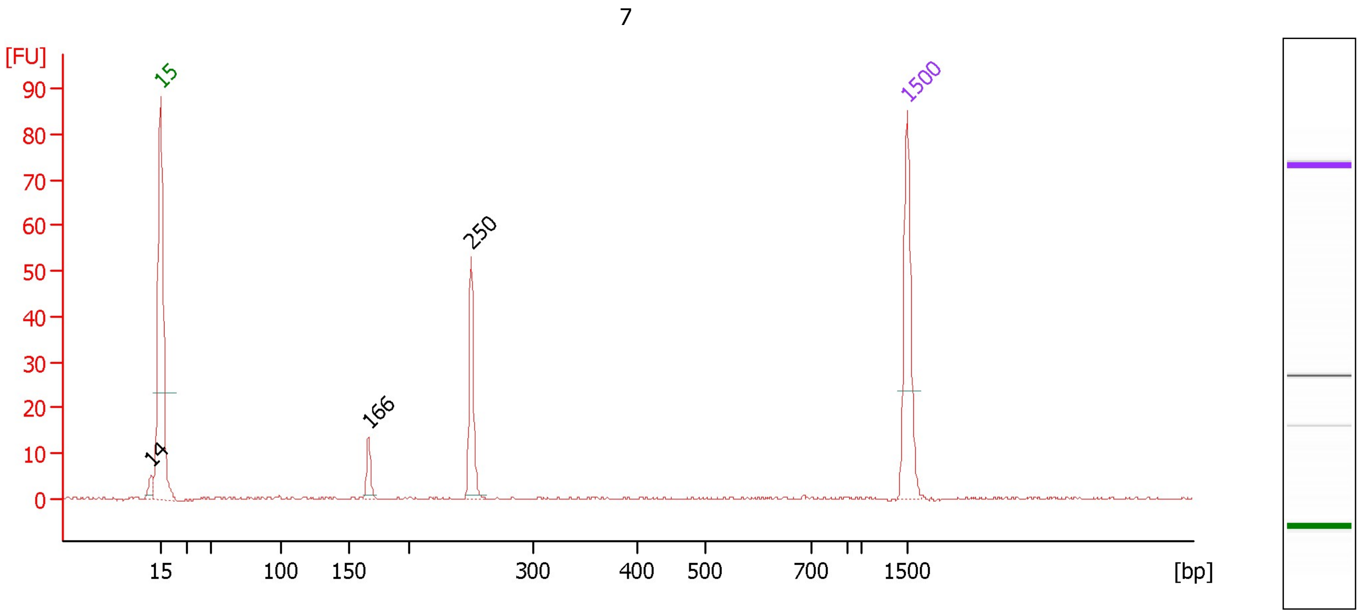

Overall Results for sample 7 : 7

Number of peaks found: 2

Peak table for sample 7 : 7

Peak		Size [bp]	Conc. [ng/µl]	Molarity [nmol/l]	Observations
1		14	0.00	0.0	
2	◀	15	4.20	424.2	Lower Marker
3		166	0.41	3.8	
4		250	1.42	8.6	
5	▶	1,500	2.10	2.1	Upper Marker

 © Copyright 2003 - 2009 Agilent Technologies, Inc.

Assay Class: DNA 1000
Data Path: C:\...-26\2100 expert_DNA 1000_DE13804763_2023-04-26_13-07-53.xad

Created: 4/26/2023 1:07:52 PM
Modified: 4/26/2023 1:50:45 PM

Electropherogram Summary Continued ...

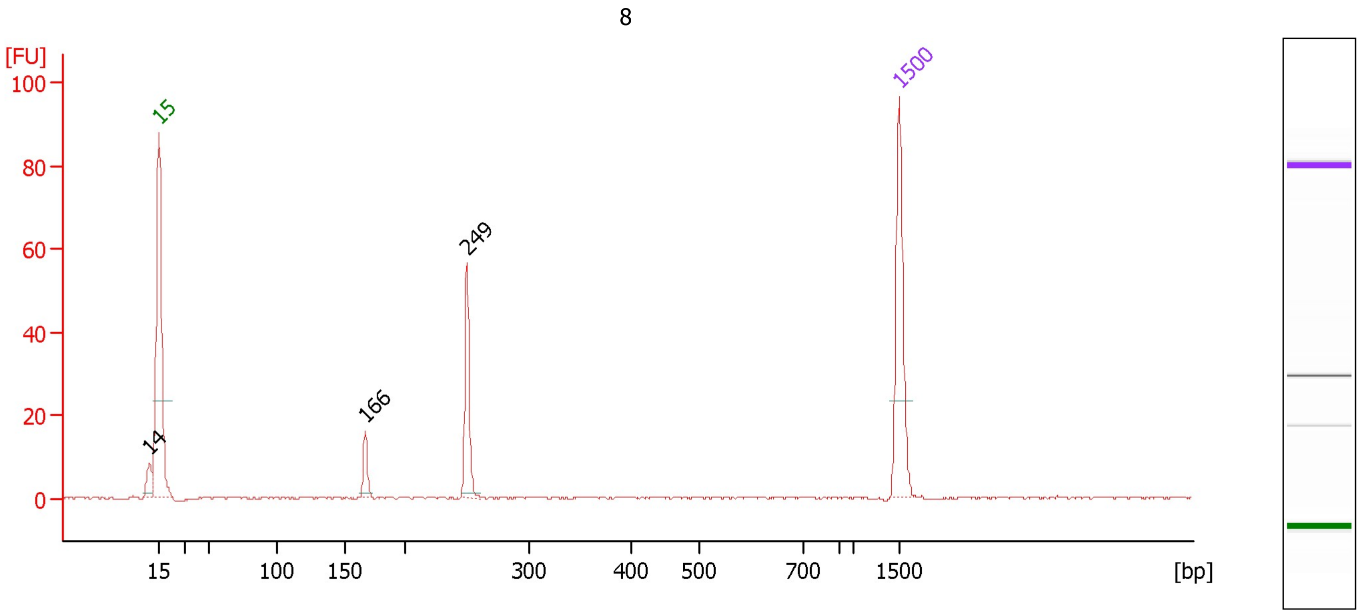

Overall Results for sample 8 : 8

Number of peaks found: 2

Peak table for sample 8 : 8

Peak		Size [bp]	Conc. [ng/µl]	Molarity [nmol/l]	Observations
1		14	0.00	0.0	
2	◀	15	4.20	424.2	Lower Marker
3		166	0.42	3.9	
4		249	1.32	8.0	
5	▶	1,500	2.10	2.1	Upper Marker

 © Copyright 2003 - 2009 Agilent Technologies, Inc.

Assay Class: DNA 1000 Created: 4/26/2023 1:07:52 PM
Data Path: C:\...-26\2100 expert_DNA 1000_DE13804763_2023-04-26_13-07-53.xad Modified: 4/26/2023 1:50:45 PM

Electropherogram Summary Continued ...

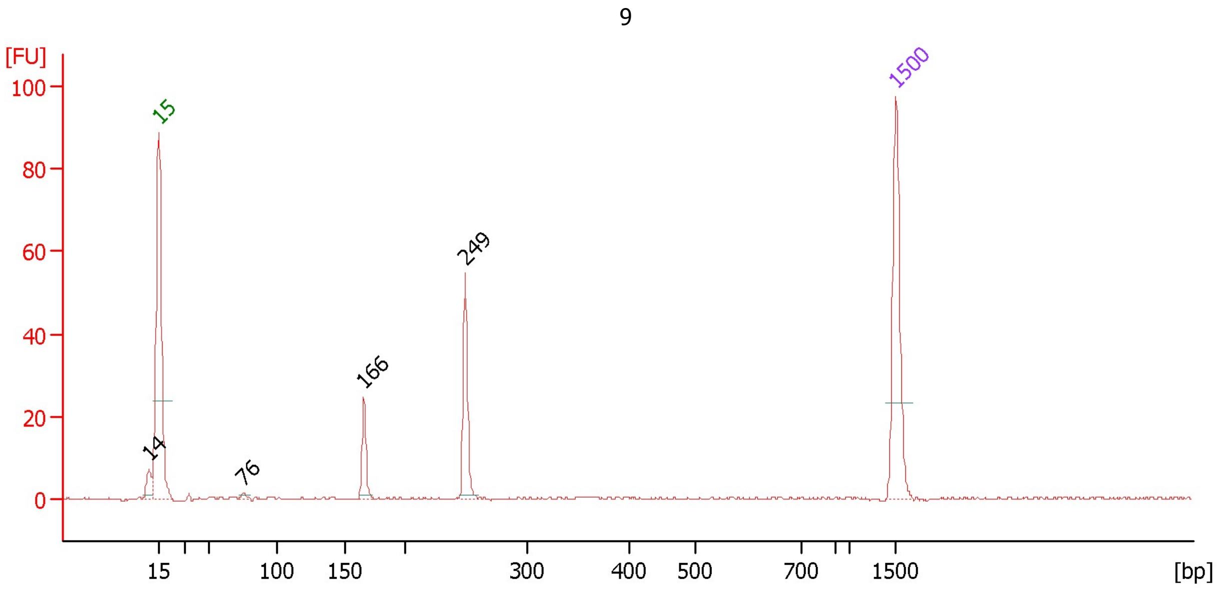

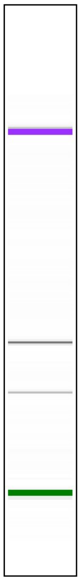

Overall Results for sample 9 : 9

Number of peaks found: 3

Peak table for sample 9 : 9

Peak		Size [bp]	Conc. [ng/µl]	Molarity [nmol/l]	Observations
1		14	0.00	0.0	
2	◀	15	4.20	424.2	Lower Marker
3		76	0.05	0.9	
4		166	0.62	5.6	
5		249	1.25	7.6	
6	▶	1,500	2.10	2.1	Upper Marker

 © Copyright 2003 - 2009 Agilent Technologies, Inc.

Assay Class: DNA 1000 Created: 4/26/2023 1:07:52 PM
Data Path: C:\...-26\2100 expert_DNA 1000_DE13804763_2023-04-26_13-07-53.xad Modified: 4/26/2023 1:50:45 PM

Electropherogram Summary Continued ...

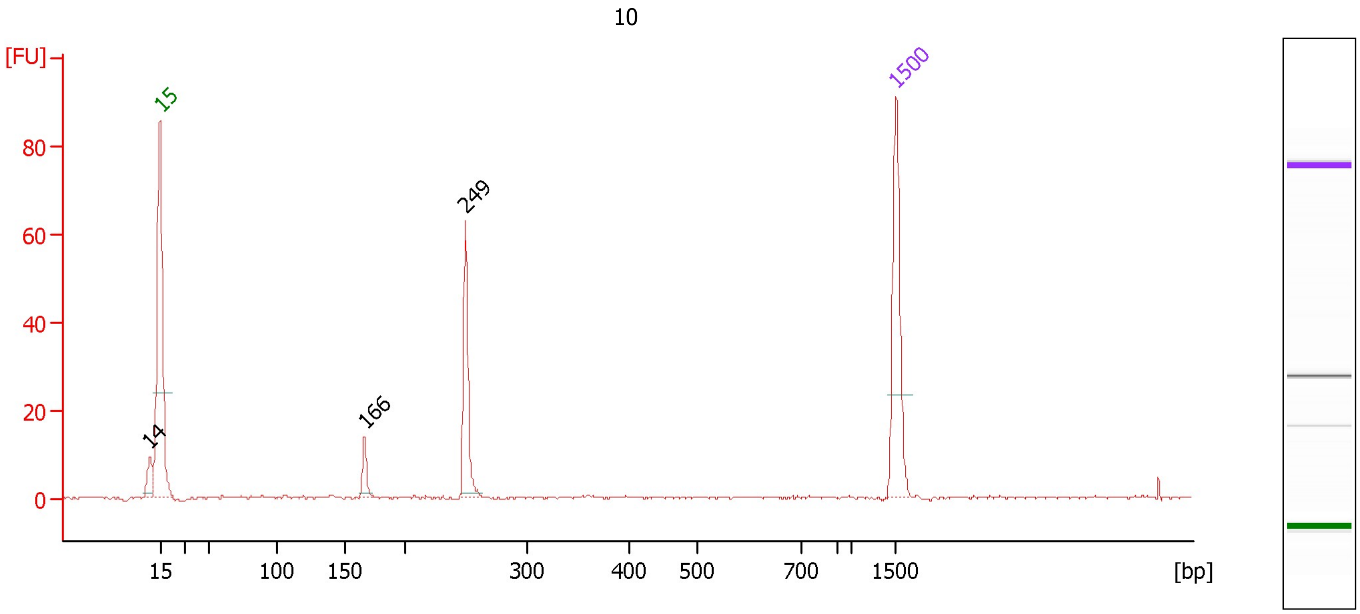

Overall Results for sample 10 : 10

Number of peaks found: 2

Peak table for sample 10 : 10

Peak		Size [bp]	Conc. [ng/µl]	Molarity [nmol/l]	Observations
1		14	0.00	0.0	
2	◀	15	4.20	424.2	Lower Marker
3		166	0.36	3.3	
4		249	1.60	9.7	
5	▶	1,500	2.10	2.1	Upper Marker

 © Copyright 2003 - 2009 Agilent Technologies, Inc.

Assay Class: DNA 1000
Data Path: C:\...-26\2100 expert_DNA 1000_DE13804763_2023-04-26_13-07-53.xad

Created: 4/26/2023 1:07:52 PM
Modified: 4/26/2023 1:50:45 PM

Electropherogram Summary Continued ...

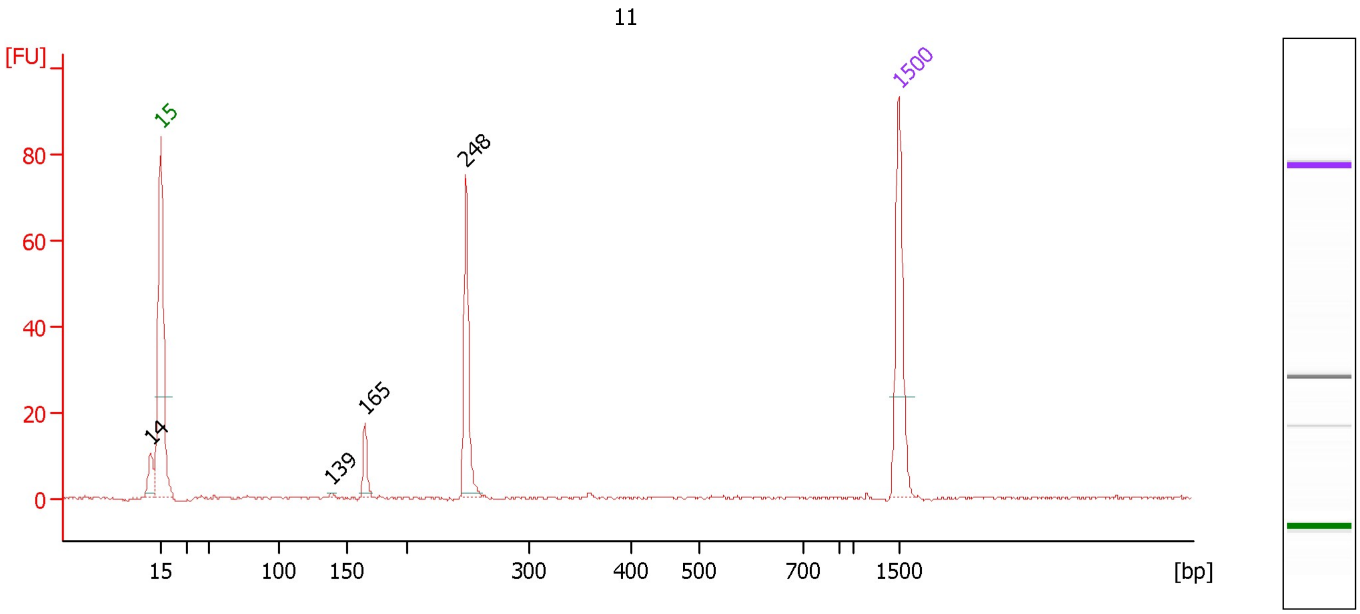

Overall Results for sample 11 : 11

Number of peaks found: 3

Peak table for sample 11 : 11

Peak		Size [bp]	Conc. [ng/µl]	Molarity [nmol/l]	Observations
1		14	0.00	0.0	
2	◀	15	4.20	424.2	Lower Marker
3		139	0.03	0.3	
4		165	0.46	4.2	
5		248	1.93	11.8	
6	▶	1,500	2.10	2.1	Upper Marker

© Copyright 2003 - 2009 Agilent Technologies, Inc.

Assay Class: DNA 1000 Created: 4/26/2023 1:07:52 PM
Data Path: C:\...-26\2100 expert_DNA 1000_DE13804763_2023-04-26_13-07-53.xad Modified: 4/26/2023 1:50:45 PM

Electropherogram Summary Continued ...

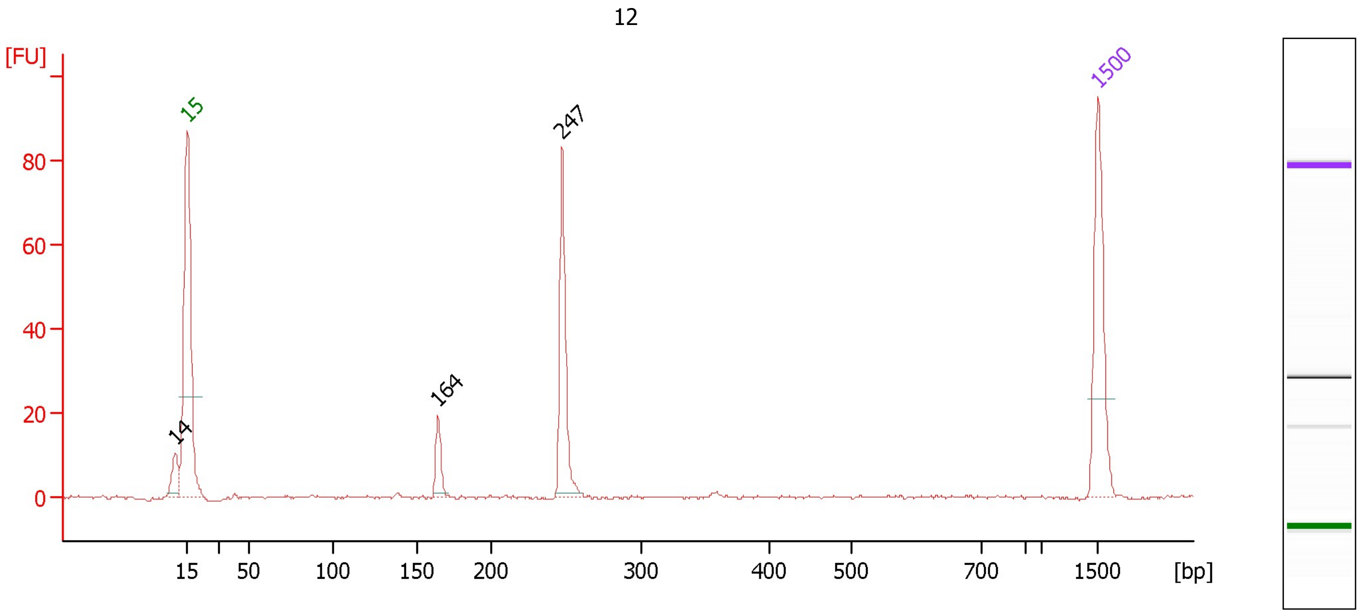

Overall Results for sample 12 : 12

Number of peaks found: 2

Peak table for sample 12 : 12

Peak		Size [bp]	Conc. [ng/µl]	Molarity [nmol/l]	Observations
1		14	0.00	0.0	
2	◀	15	4.20	424.2	Lower Marker
3		164	0.48	4.4	
4		247	2.01	12.3	
5	▶	1,500	2.10	2.1	Upper Marker

 © Copyright 2003 - 2009 Agilent Technologies, Inc.

Assay Class: DNA 1000
Data Path: C:\...-26\2100 expert_DNA 1000_DE13804763_2023-04-26_13-07-53.xad
Created: 4/26/2023 1:07:52 PM
Modified: 4/26/2023 1:50:45 PM

Gel Image

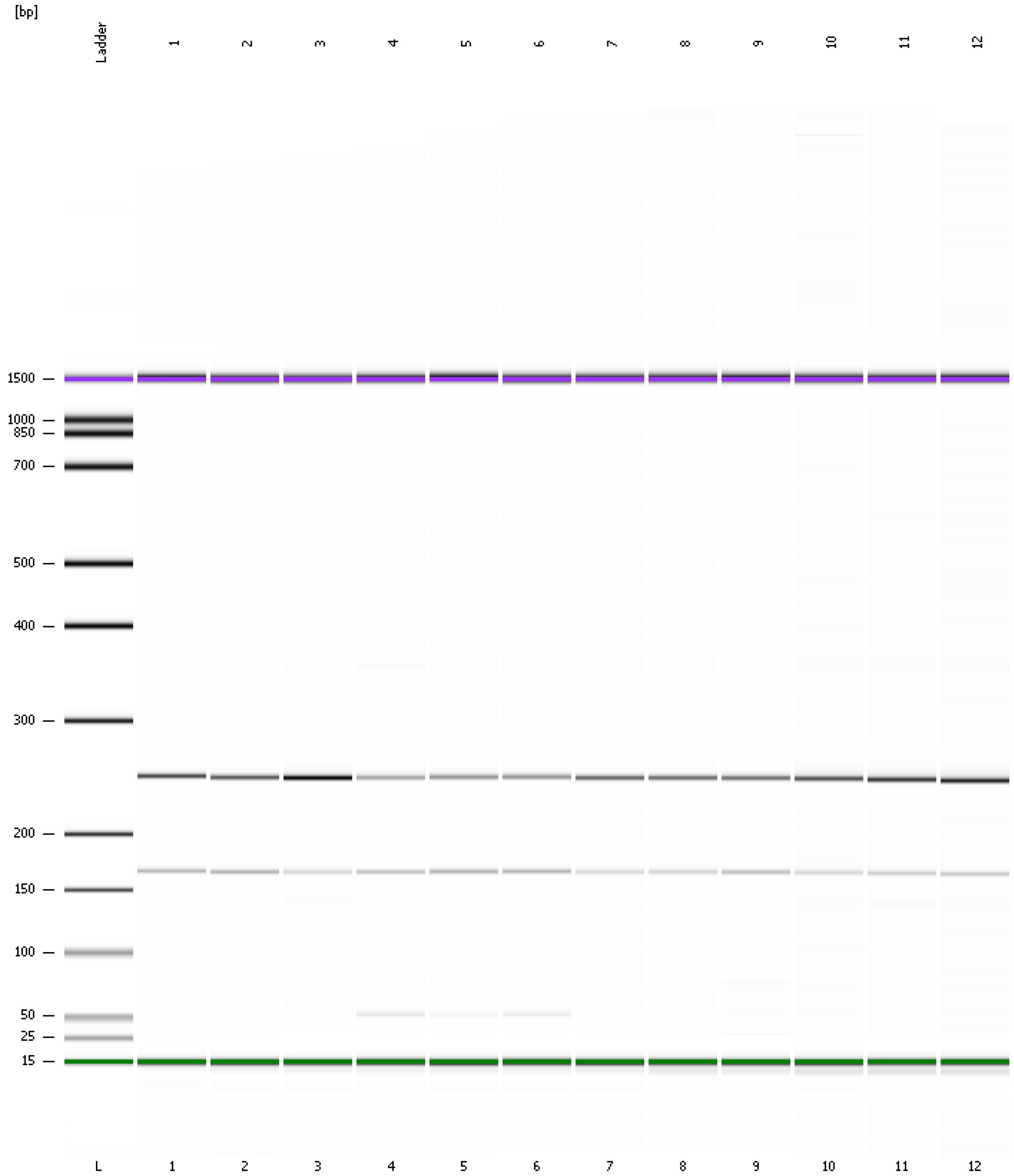

Supplement: Figure 2—figure supplement 1—source data 2. [file elife-103167-fig2-figsupp1-data2.zip › SupplFigS2/hek_qki_sf1_overexpression.pdf]
