# Supplementary material for: Competition for the conserved branch point sequence influences physiological outcomes in pre-mRNA splicing
Source: eLife. 2026 Mar 20;13:RP103167. doi: 10.7554/eLife.103167 (PMC13004596; doi:10.7554/eLife.103167)
Supplement: Figure 3—source data 1. [file elife-103167-fig3-data1.pdf]

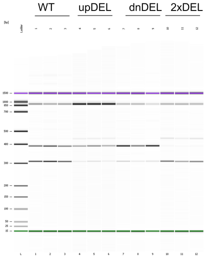

Figure 3—source data 1. Original capillary electrophoresis (BioAnalyzer) images for Figure 3B. Original BioAnalyzer gel-like image corresponding to Figure 3, panel B, showing the splicing patterns of RAI14 ex 11 reporters. Lane L corresponds to the molecular weight ladder with sizes indicated in base pairs (bp). Lanes 1–12 correspond to the biological triplicates of WT, upDEL, dnDEL, and 2xDEL reporter.

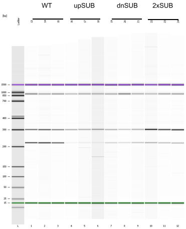

Figure 3—source data 2. Original capillary electrophoresis (BioAnalyzer) images for Figure 3D. Original BioAnalyzer gel-like image corresponding to Figure 3, panel D, showing the splicing patterns of RAI14 ex 11 substitution mutant reporters. Lane L corresponds to the molecular weight ladder with sizes indicated in base pairs (bp). Lanes 1–12 correspond to the biological triplicates of WT, upSUB, dnSUB, and 2xSUB reporter constructs

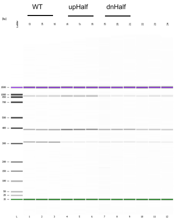

Figure 3—source data 3. Original capillary electrophoresis (BioAnalyzer) images for Figure 3F. Original BioAnalyzer gel-like image corresponding to Figure 3, panel F, showing the splicing patterns of RAI14 ex 11 half-site and motif deletion mutant reporters. Lane L corresponds to the molecular weight ladder with sizes indicated in base pairs (bp). Lanes 1–9 correspond to the biological triplicates of WT, upHalfsite, and dnHalfsite reporter constructs as described in the main manuscript text. Lanes 10–12 correspond to a mutant construct with a deletion of the second ACU motif ; these lanes were not depicted in the final version of Figure 3F.

Figure 3, Source Data 1. Original BioAnalyzer gel-like images corresponding to Figure 3A (left), Figure 3B (middle), and Figure 3C (right). See text under each for additional details.
