# Supplementary material for: Competition for the conserved branch point sequence influences physiological outcomes in pre-mRNA splicing
Source: eLife. 2026 Mar 20;13:RP103167. doi: 10.7554/eLife.103167 (PMC13004596; doi:10.7554/eLife.103167)
Supplement: Figure 3—source data 2. [file elife-103167-fig3-data2.zip › Fig3/112922_DUP-RAI14-SubMTs_C2C12_BioAnalyzer.pdf]

Assay Class: DNA 1000
Data Path: C:\...-29\2100 expert_DNA 1000_DE13804763_2022-11-29_13-43-34.xad

Created: 11/29/2022 1:43:34 PM
Modified: 11/29/2022 2:26:21 PM

Electrophoresis File Run Summary

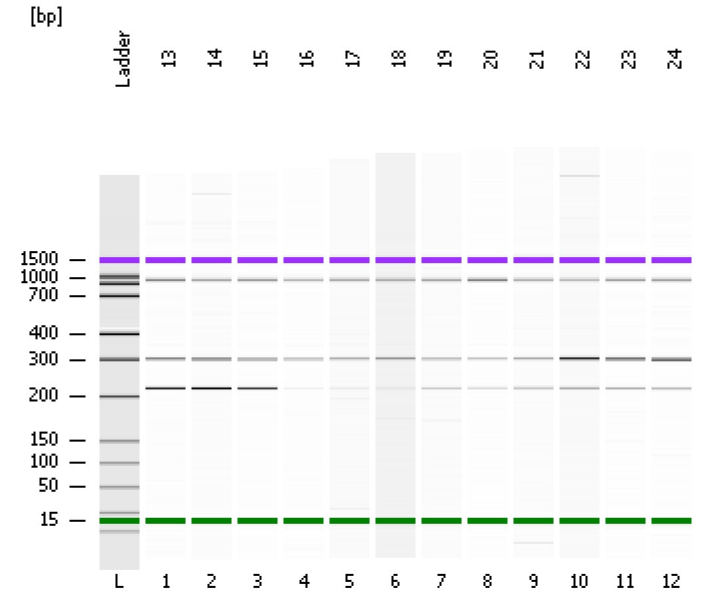

Instrument Information:

Instrument Name: DE13804763
Firmware: C.01.069
Serial#: DE13804763
Type: G2939A

Assay Information:

Assay Origin Path: C:\Program Files\Agilent\2100 bioanalyzer\2100 expert\assays\dsDNA\DNA 1000 Series II.xsy

Assay Class: DNA 1000

Version: 2.3

Assay Comments: DNA Analysis 25 -1000 bp

© Copyright 2003-2009 Agilent Technologies, Inc.

Chip Information:

Chip Lot #:

Reagent Kit Lot #:

Chip Comments:

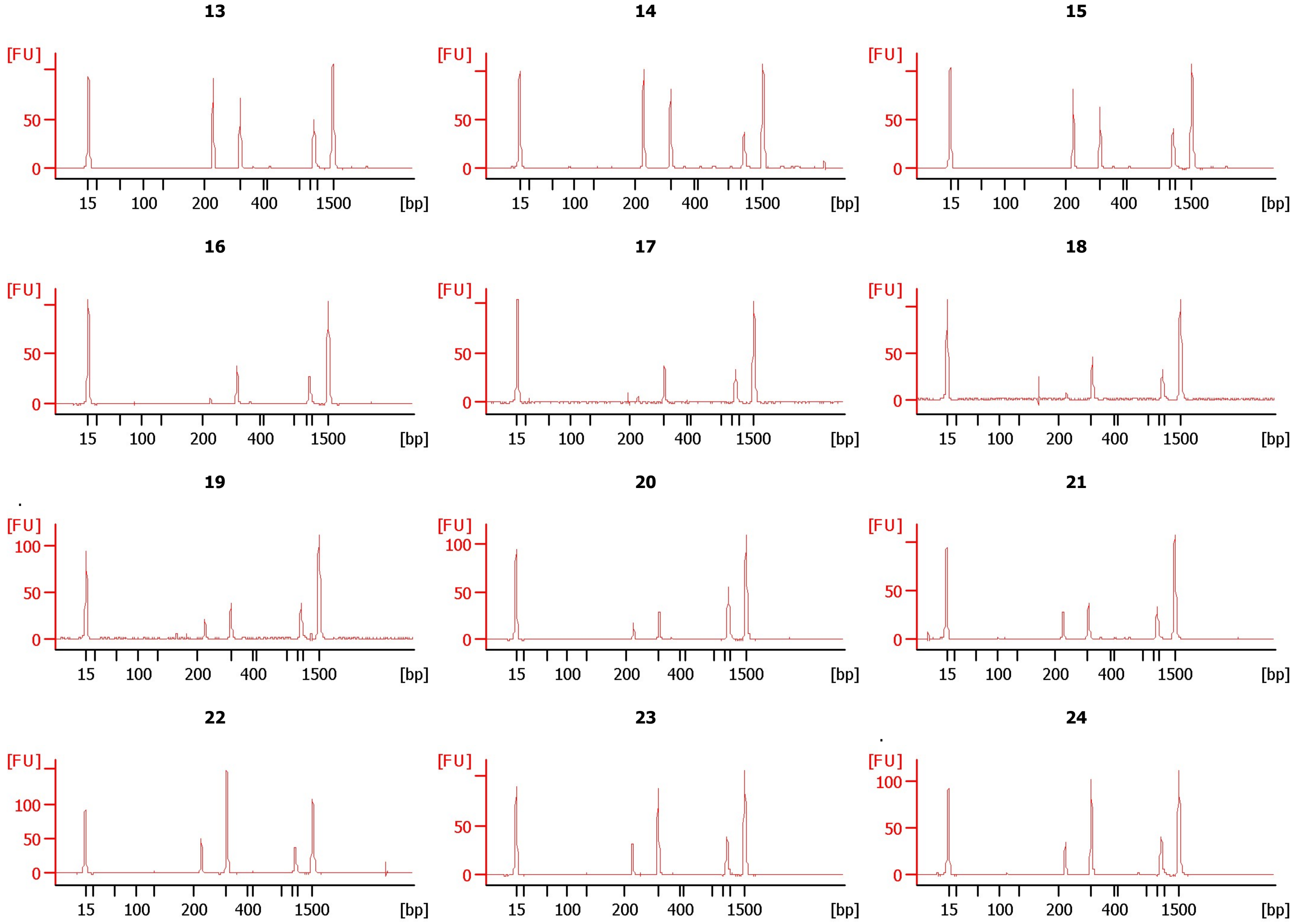

© Copyright 2003 - 2009 Agilent Technologies, Inc.

Assay Class: DNA 1000
Data Path: C:\...-29\2100 expert_DNA 1000_DE13804763_2022-11-29_13-43-34.xad
Created: 11/29/2022 1:43:34 PM
Modified: 11/29/2022 2:26:21 PM

Electrophoresis File Run Summary (Chip Summary)

Sample Name	Sample Comment	Rest. Digest	Status	Observation	Result Label	Result Color
13		☐	✓			
14		☐	✓			
15		☐	✓			
16		☐	✓			
17		☐	✓			
18		☐	✓			
19	.	☐	✓			
20		☐	✓			
21		☐	✓			
22		☐	✓			
23		☐	✓			
24	.	☐	✓			
Ladder		☐	✓			

Chip Lot # **Reagent Kit Lot #**

Chip Comments :

 © Copyright 2003 - 2009 Agilent Technologies, Inc.

Assay Class: DNA 1000
Data Path: C:\...-29\2100 expert_DNA 1000_DE13804763_2022-11-29_13-43-34.xad
Created: 11/29/2022 1:43:34 PM
Modified: 11/29/2022 2:26:21 PM

Electrophoresis Assay Details

General Analysis Settings

Number of Available Sample and Ladder Wells (Max.) : 13
Minimum Visible Range [s] : 30
Maximum Visible Range [s] : 129
Start Analysis Time Range [s] : 30
End Analysis Time Range [s] : 128.95
Ladder Concentration [ng/µl] : 44
Uses Standard Area for Ladder Fragments
Lower Marker Concentration [ng/µl] : 4.2
Upper Marker Concentration [ng/µl] : 2.1
Used Upper Marker for Quantitation
Standard Curve Fit is Point to Point
Show Data Aligned to Lower and Upper Marker

Integrator Settings

Integration Start Time [s] : 30
Integration End Time [s] : 128.95
Slope Threshold : 0.5
Height Threshold [FU] : 1
Area Threshold : 0.1
Width Threshold [s] : 0.5
Baseline Plateau [s] : 0.5

Filter Settings

Filter Width [s] : 0.5
Polynomial Order : 4

Ladder

Ladder Peak	Size	Area
1	15	25
2	25	26
3	50	34
4	100	41
5	150	45
6	200	52
7	300	63
8	400	76
9	500	83
10	700	88
11	850	86
12	1000	90
13	1500	52

 © Copyright 2003 - 2009 Agilent Technologies, Inc.

Assay Class: DNA 1000
Data Path: C:\...-29\2100 expert_DNA 1000_DE13804763_2022-11-29_13-43-34.xad
Created: 11/29/2022 1:43:34 PM
Modified: 11/29/2022 2:26:21 PM

Electropherogram Summary

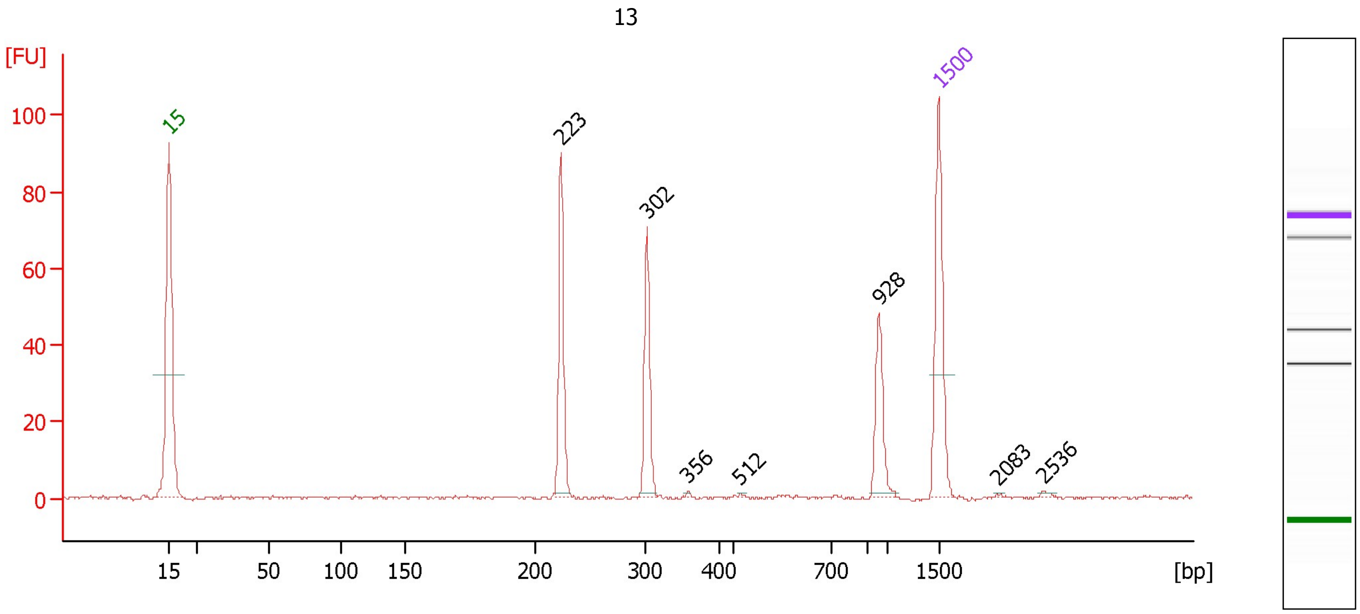

Overall Results for sample 1 : 13

Number of peaks found: 5

Peak table for sample 1 : 13

Peak		Size [bp]	Conc. [ng/µl]	Molarity [nmol/l]	Observations
1	◀	15	4.20	424.2	Lower Marker
2		223	2.16	14.6	
3		302	1.61	8.1	
4		356	0.02	0.1	
5		512	0.01	0.0	
6		928	1.14	1.9	
7	▶	1,500	2.10	2.1	Upper Marker
8		2,083	0.00	0.0	
9		2,536	0.00	0.0	

© Copyright 2003 - 2009 Agilent Technologies, Inc.

Assay Class: DNA 1000
Data Path: C:\...-29\2100 expert_DNA 1000_DE13804763_2022-11-29_13-43-34.xad

Created: 11/29/2022 1:43:34 PM
Modified: 11/29/2022 2:26:21 PM

Electropherogram Summary Continued ...

14

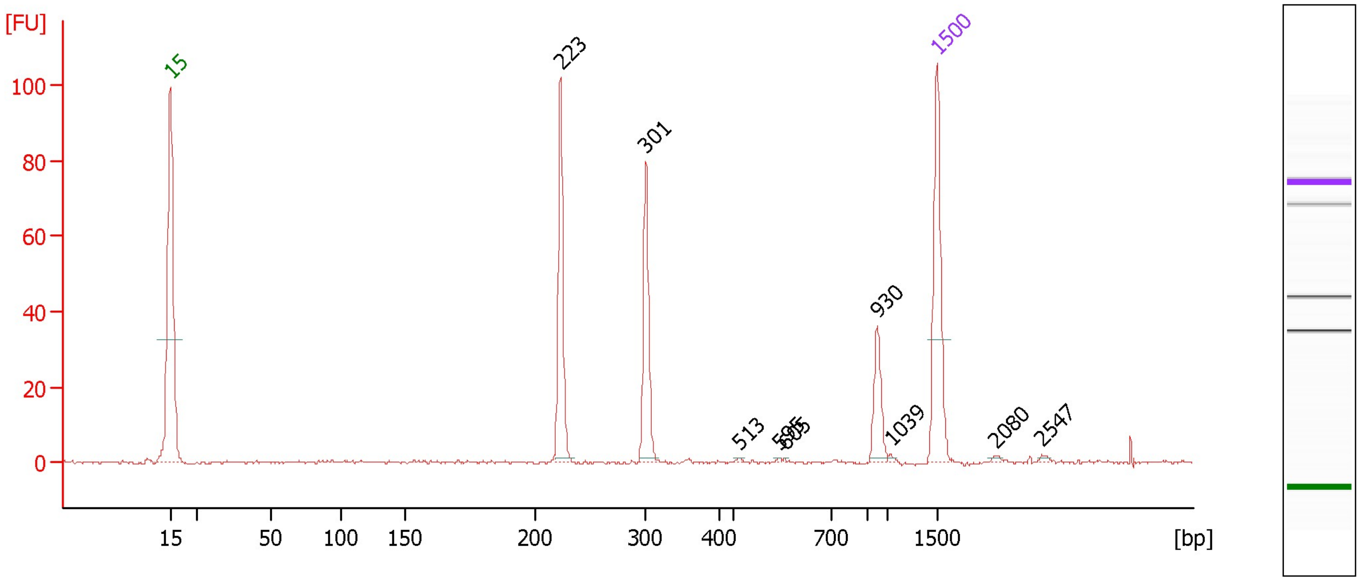

Overall Results for sample 2 : 14

Number of peaks found: 7

Peak table for sample 2 : 14

Peak		Size [bp]	Conc. [ng/µl]	Molarity [nmol/l]	Observations
1	◀	15	4.20	424.2	Lower Marker
2		223	2.44	16.5	
3		301	1.86	9.4	
4		513	0.02	0.1	
5		595	0.02	0.0	
6		605	0.01	0.0	
7		930	0.81	1.3	
8		1,039	0.03	0.0	
9	▶	1,500	2.10	2.1	Upper Marker
10		2,080	0.00	0.0	
11		2,547	0.00	0.0	

© Copyright 2003 - 2009 Agilent Technologies, Inc.

Assay Class: DNA 1000
Data Path: C:\...-29\2100 expert_DNA 1000_DE13804763_2022-11-29_13-43-34.xad

Created: 11/29/2022 1:43:34 PM
Modified: 11/29/2022 2:26:21 PM

Electropherogram Summary Continued ...

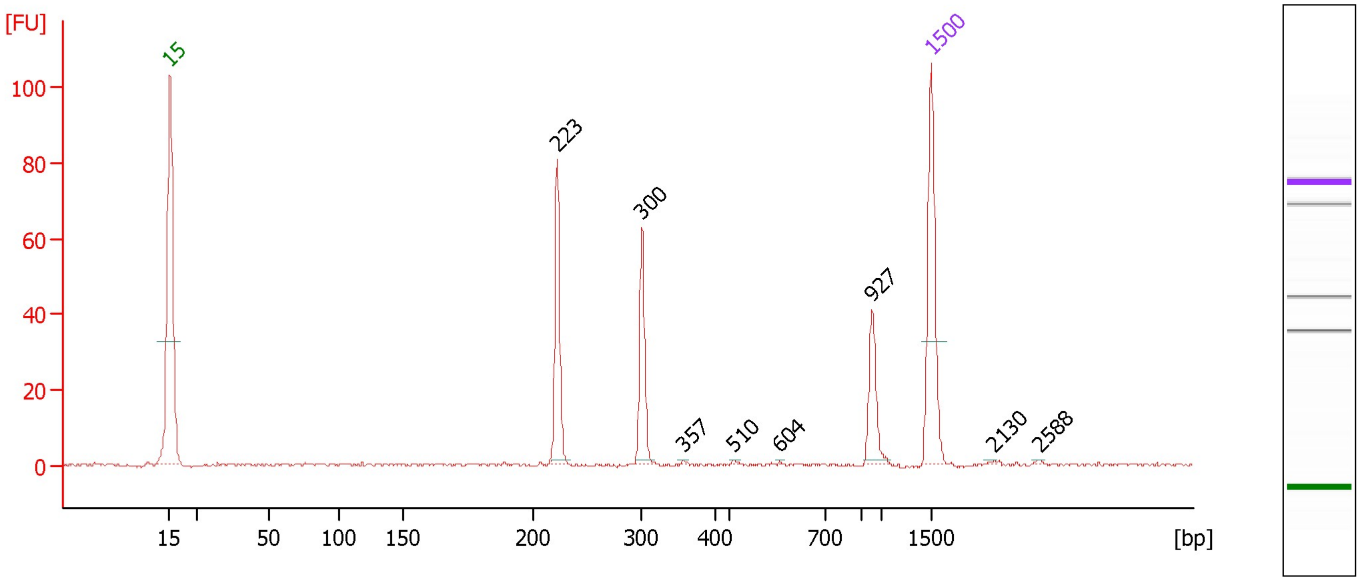

Overall Results for sample 3 : 15

Number of peaks found: 6

Peak table for sample 3 : 15

Peak		Size [bp]	Conc. [ng/µl]	Molarity [nmol/l]	Observations
1	◀	15	4.20	424.2	Lower Marker
2		223	1.94	13.2	
3		300	1.40	7.1	
4		357	0.02	0.1	
5		510	0.02	0.1	
6		604	0.01	0.0	
7		927	0.96	1.6	
8	▶	1,500	2.10	2.1	Upper Marker
9		2,130	0.00	0.0	
10		2,588	0.00	0.0	

 © Copyright 2003 - 2009 Agilent Technologies, Inc.

Assay Class: DNA 1000 Created: 11/29/2022 1:43:34 PM
Data Path: C:\...-29\2100 expert_DNA 1000_DE13804763_2022-11-29_13-43-34.xad Modified: 11/29/2022 2:26:21 PM

Electropherogram Summary Continued ...

16

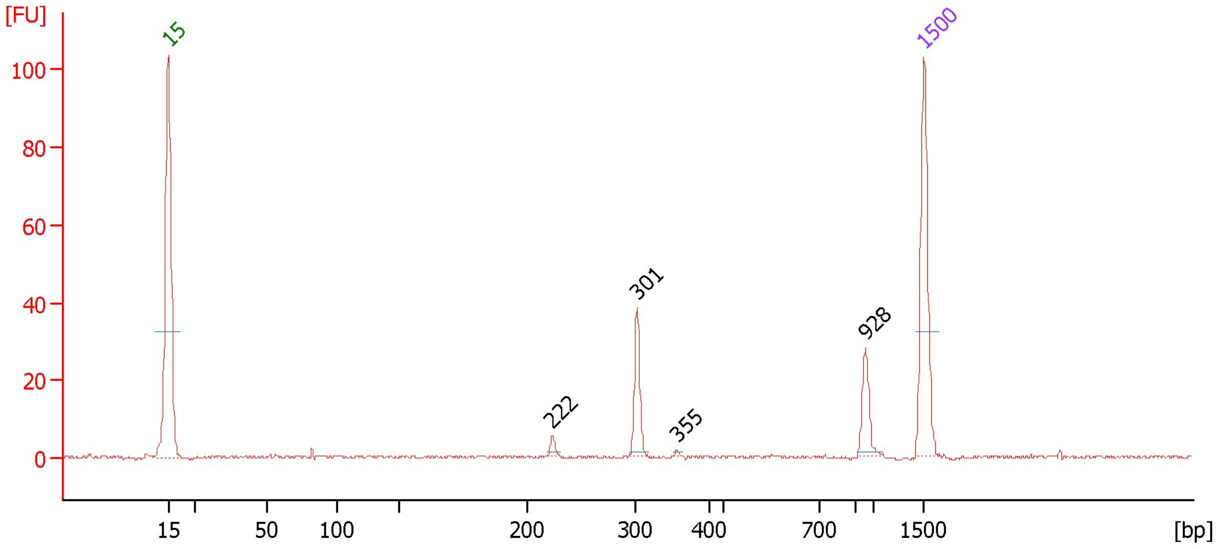

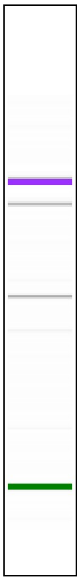

Overall Results for sample 4 : **16**

Number of peaks found: 4

Peak table for sample 4 : **16**

Peak		Size [bp]	Conc. [ng/µl]	Molarity [nmol/l]	Observations
1	◀	15	4.20	424.2	Lower Marker
2		222	0.14	0.9	
3		301	0.91	4.6	
4		355	0.03	0.1	
5		928	0.64	1.1	
6	▶	1,500	2.10	2.1	Upper Marker

 © Copyright 2003 - 2009 Agilent Technologies, Inc.

Assay Class: DNA 1000
Data Path: C:\...-29\2100 expert_DNA 1000_DE13804763_2022-11-29_13-43-34.xad

Created: 11/29/2022 1:43:34 PM
Modified: 11/29/2022 2:26:21 PM

Electropherogram Summary Continued ...

17

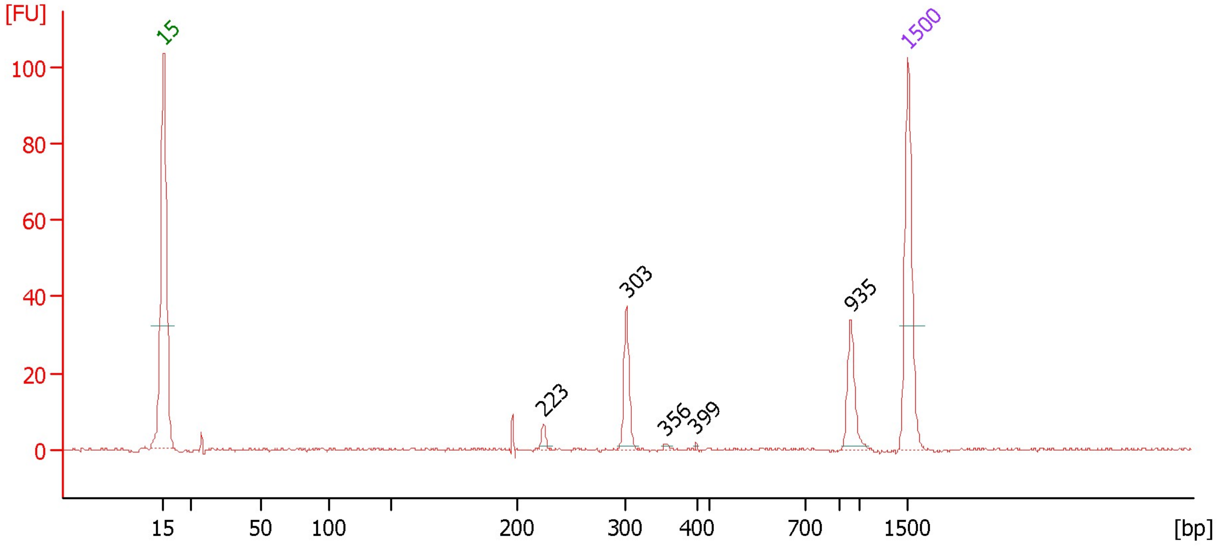

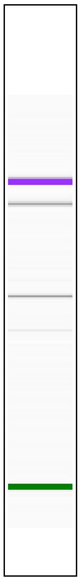

Overall Results for sample 5 : __17__

Number of peaks found: 5

Peak table for sample 5 : __17__

Peak		Size [bp]	Conc. [ng/µl]	Molarity [nmol/l]	Observations
1	◀	15	4.20	424.2	Lower Marker
2		223	0.16	1.1	
3		303	0.90	4.5	
4		356	0.04	0.2	
5		399	0.01	0.1	
6		935	0.80	1.3	
7	▶	1,500	2.10	2.1	Upper Marker

© Copyright 2003 - 2009 Agilent Technologies, Inc.

Assay Class: DNA 1000
Data Path: C:\...-29\2100 expert_DNA 1000_DE13804763_2022-11-29_13-43-34.xad

Created: 11/29/2022 1:43:34 PM
Modified: 11/29/2022 2:26:21 PM

Electropherogram Summary Continued ...

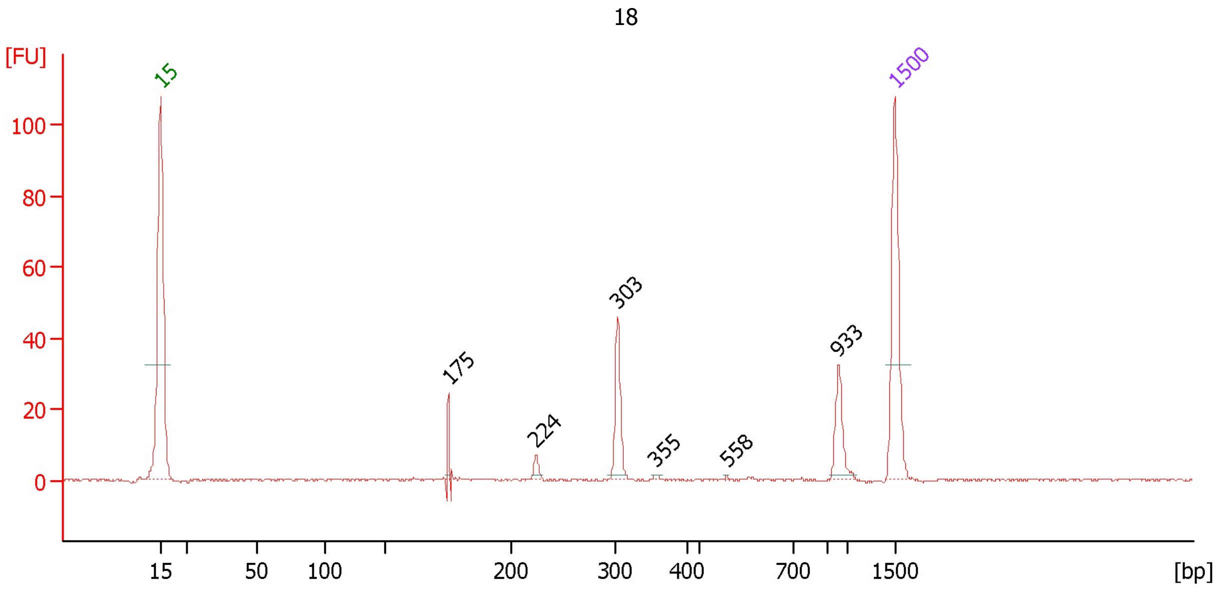

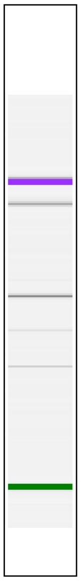

Overall Results for sample 6 : 18

Number of peaks found: 6

Peak table for sample 6 : 18

Peak		Size [bp]	Conc. [ng/µl]	Molarity [nmol/l]	Observations
1	◀	15	4.20	424.2	Lower Marker
2		175	0.24	2.1	
3		224	0.16	1.1	
4		303	1.05	5.2	
5		355	0.03	0.1	
6		558	0.01	0.0	
7		933	0.72	1.2	
8	▶	1,500	2.10	2.1	Upper Marker

 © Copyright 2003 - 2009 Agilent Technologies, Inc.

Assay Class: DNA 1000
Data Path: C:\...-29\2100 expert_DNA 1000_DE13804763_2022-11-29_13-43-34.xad
Created: 11/29/2022 1:43:34 PM
Modified: 11/29/2022 2:26:21 PM

Electropherogram Summary Continued ...

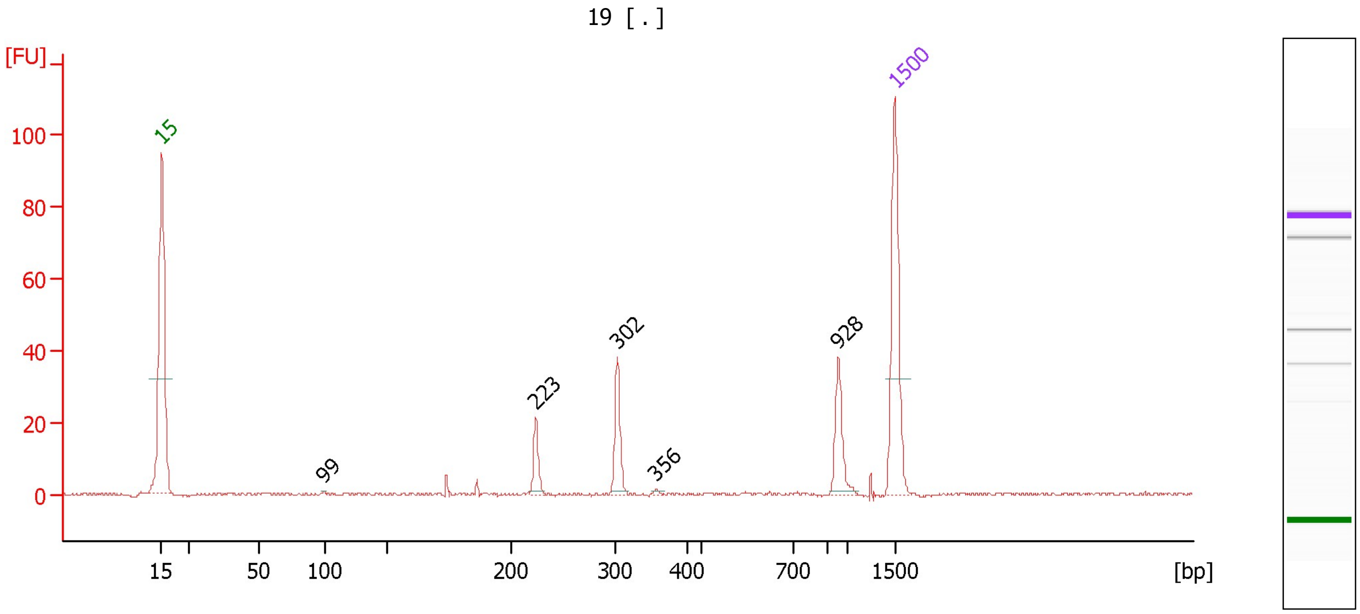

Overall Results for sample 7 : 19

Number of peaks found: 5

Peak table for sample 7 : 19

Peak		Size [bp]	Conc. [ng/µl]	Molarity [nmol/l]	Observations
1	◀	15	4.20	424.2	Lower Marker
2		99	0.01	0.2	
3		223	0.48	3.3	
4		302	0.83	4.1	
5		356	0.04	0.2	
6		928	0.79	1.3	
7	▶	1,500	2.10	2.1	Upper Marker

© Copyright 2003 - 2009 Agilent Technologies, Inc.

Assay Class: DNA 1000
Data Path: C:\...-29\2100 expert_DNA 1000_DE13804763_2022-11-29_13-43-34.xad

Created: 11/29/2022 1:43:34 PM
Modified: 11/29/2022 2:26:21 PM

Electropherogram Summary Continued ...

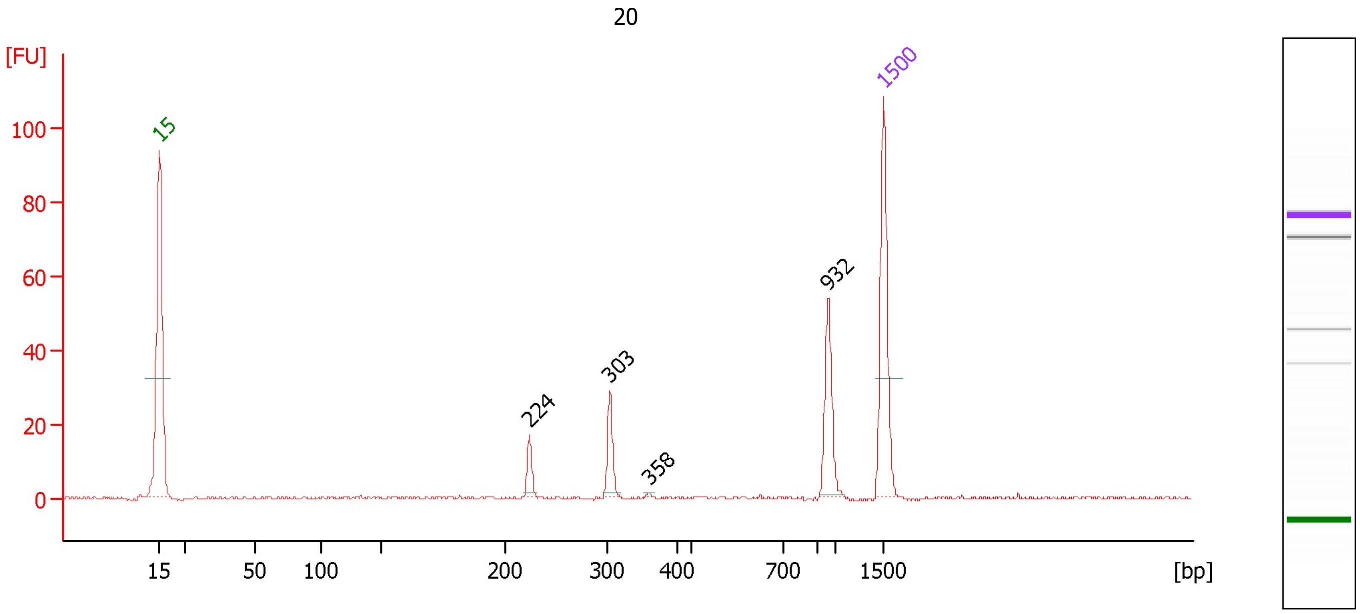

Overall Results for sample 8 : __20__

Number of peaks found: 4

Peak table for sample 8 : __20__

Peak		Size [bp]	Conc. [ng/µl]	Molarity [nmol/l]	Observations
1	◀	15	4.20	424.2	Lower Marker
2		224	0.39	2.6	
3		303	0.65	3.2	
4		358	0.02	0.1	
5		932	1.22	2.0	
6	▶	1,500	2.10	2.1	Upper Marker

 © Copyright 2003 - 2009 Agilent Technologies, Inc.

Assay Class: DNA 1000
Data Path: C:\...-29\2100 expert_DNA 1000_DE13804763_2022-11-29_13-43-34.xad
Created: 11/29/2022 1:43:34 PM
Modified: 11/29/2022 2:26:21 PM

Electropherogram Summary Continued ...

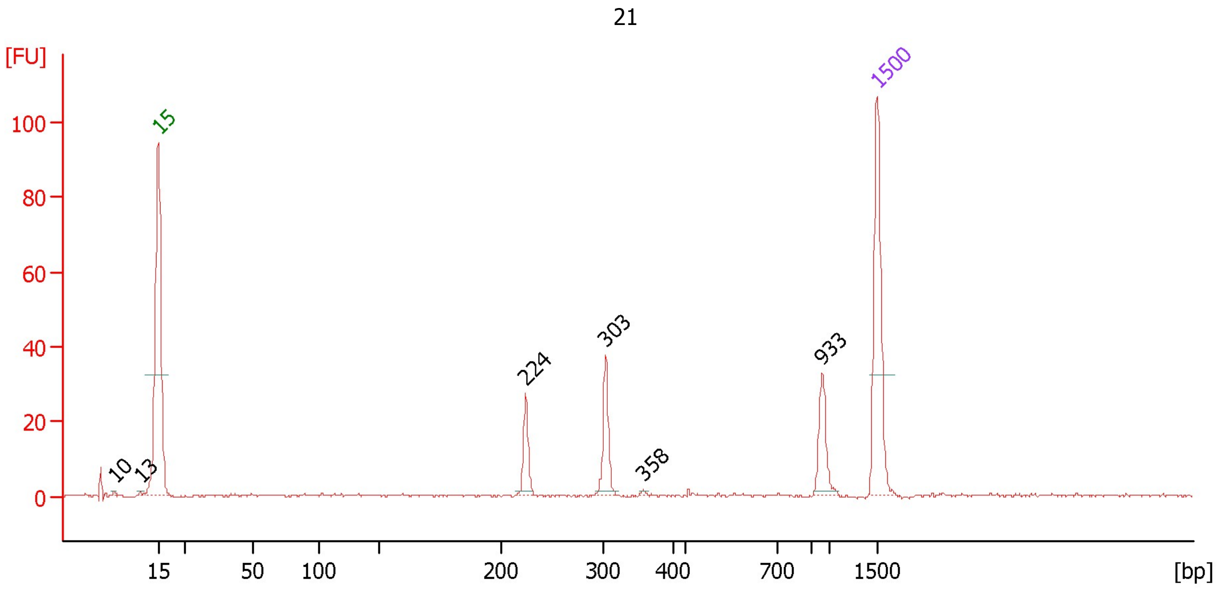

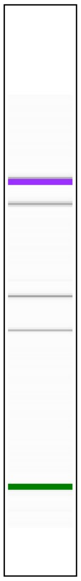

Overall Results for sample 9 : 21

Number of peaks found: 4

Peak table for sample 9 : 21

Peak		Size [bp]	Conc. [ng/µl]	Molarity [nmol/l]	Observations
1		10	0.00	0.0	
2		13	0.00	0.0	
3	◀	15	4.20	424.2	Lower Marker
4		224	0.64	4.4	
5		303	0.85	4.2	
6		358	0.03	0.1	
7		933	0.74	1.2	
8	▶	1,500	2.10	2.1	Upper Marker

 © Copyright 2003 - 2009 Agilent Technologies, Inc.

Assay Class: DNA 1000
Data Path: C:\...-29\2100 expert_DNA 1000_DE13804763_2022-11-29_13-43-34.xad

Created: 11/29/2022 1:43:34 PM
Modified: 11/29/2022 2:26:21 PM

Electropherogram Summary Continued ...

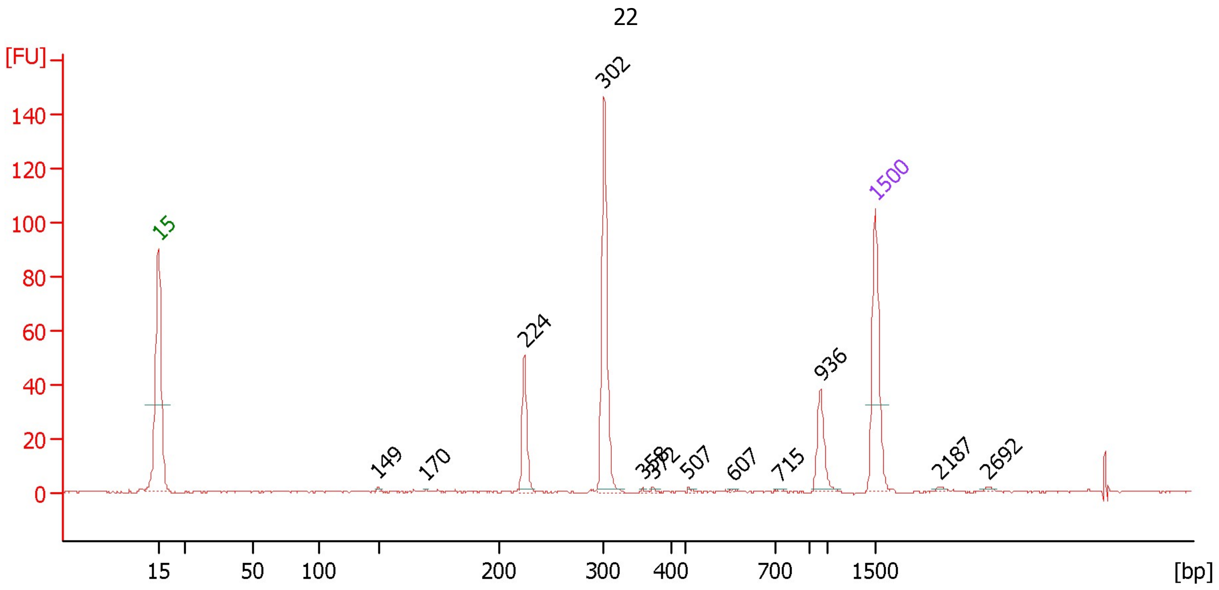

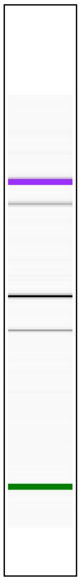

Overall Results for sample 10 : __22__

Number of peaks found: 10

Peak table for sample 10 : __22__

Peak		Size [bp]	Conc. [ng/µl]	Molarity [nmol/l]	Observations
1	◀	15	4.20	424.2	Lower Marker
2		149	0.04	0.4	
3		170	0.01	0.1	
4		224	1.24	8.4	
5		302	3.49	17.5	
6		358	0.02	0.1	
7		372	0.03	0.1	
8		507	0.02	0.1	
9		607	0.03	0.1	
10		715	0.02	0.1	
11		936	0.87	1.4	
12	▶	1,500	2.10	2.1	Upper Marker
13		2,187	0.00	0.0	
14		2,692	0.00	0.0	

 © Copyright 2003 - 2009 Agilent Technologies, Inc.

Assay Class: DNA 1000
Data Path: C:\...-29\2100 expert_DNA 1000_DE13804763_2022-11-29_13-43-34.xad

Created: 11/29/2022 1:43:34 PM
Modified: 11/29/2022 2:26:21 PM

Electropherogram Summary Continued ...

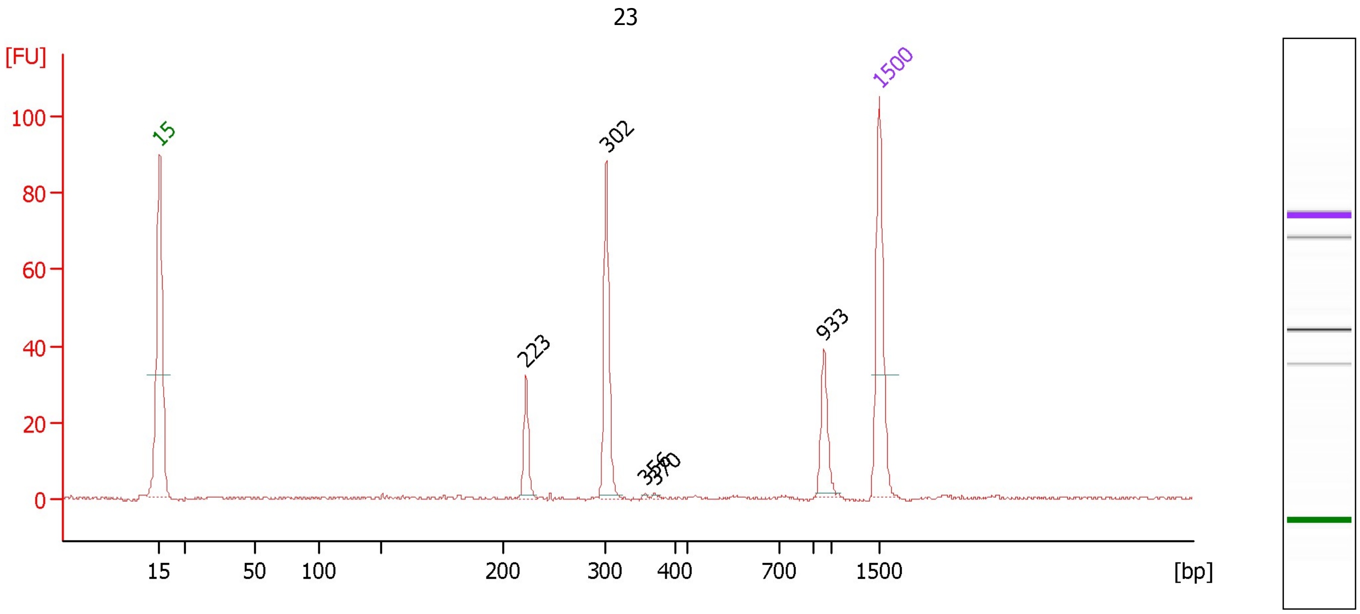

Overall Results for sample 11 : 23

Number of peaks found: 5

Peak table for sample 11 : 23

Peak		Size [bp]	Conc. [ng/µl]	Molarity [nmol/l]	Observations
1	◀	15	4.20	424.2	Lower Marker
2		223	0.76	5.2	
3		302	2.09	10.5	
4		356	0.02	0.1	
5		370	0.03	0.1	
6		933	0.85	1.4	
7	▶	1,500	2.10	2.1	Upper Marker

 © Copyright 2003 - 2009 Agilent Technologies, Inc.

Assay Class: DNA 1000
Data Path: C:\...-29\2100 expert_DNA 1000_DE13804763_2022-11-29_13-43-34.xad
Created: 11/29/2022 1:43:34 PM
Modified: 11/29/2022 2:26:21 PM

Electropherogram Summary Continued ...

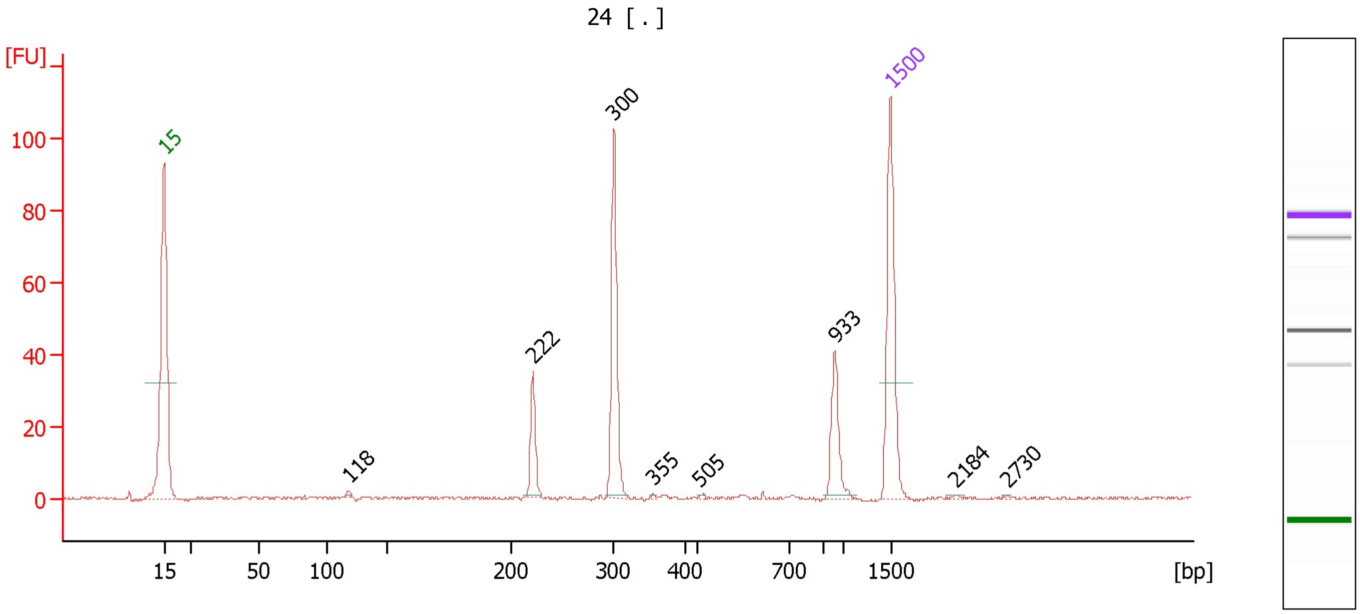

Overall Results for sample 12 : 24

Number of peaks found: 6

Peak table for sample 12 : 24

Peak		Size [bp]	Conc. [ng/µl]	Molarity [nmol/l]	Observations
1	◀	15	4.20	424.2	Lower Marker
2		118	0.05	0.6	
3		222	0.78	5.3	
4		300	2.23	11.3	
5		355	0.02	0.1	
6		505	0.02	0.1	
7		933	0.89	1.4	
8	▶	1,500	2.10	2.1	Upper Marker
9		2,184	0.00	0.0	
10		2,730	0.00	0.0	

 © Copyright 2003 - 2009 Agilent Technologies, Inc.

Assay Class: DNA 1000
Data Path: C:\...-29\2100 expert_DNA 1000_DE13804763_2022-11-29_13-43-34.xad
Created: 11/29/2022 1:43:34 PM
Modified: 11/29/2022 2:26:21 PM

Gel Image

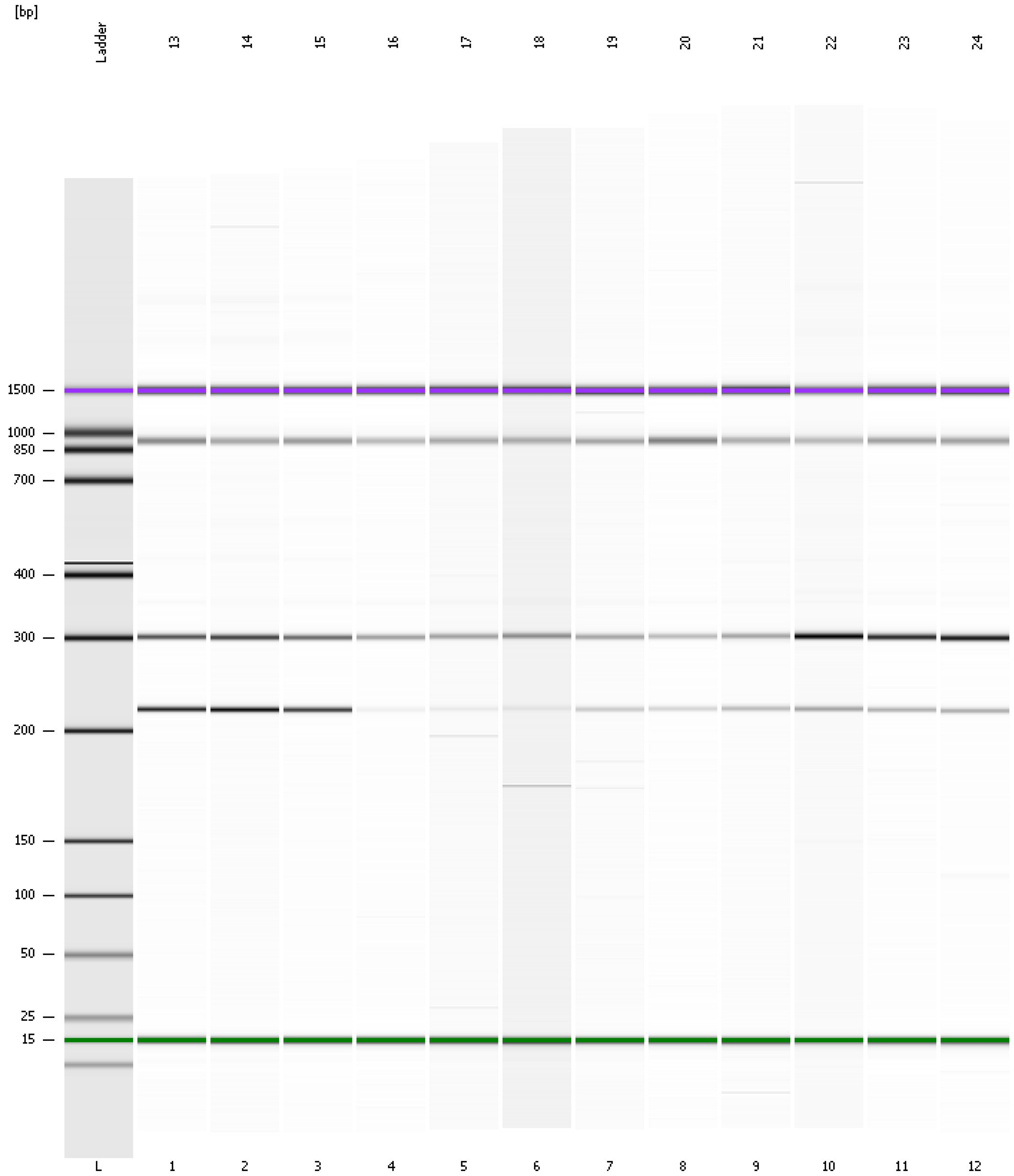

 © Copyright 2003 - 2009 Agilent Technologies, Inc.
