# Supplementary material for: Competition for the conserved branch point sequence influences physiological outcomes in pre-mRNA splicing
Source: eLife. 2026 Mar 20;13:RP103167. doi: 10.7554/eLife.103167 (PMC13004596; doi:10.7554/eLife.103167)
Supplement: Figure 3—source data 2. [file elife-103167-fig3-data2.zip › Fig3/4_28_23_C2C12_RAI14halfsite_BioAnalyzer.pdf]

Assay Class:	DNA 1000	Created:	4/26/2023 1:53:44 PM
Data Path:	C:\...-26\2100 expert_DNA 1000_DE13804763_2023-04-26_13-53-45.xad	Modified:	4/26/2023 2:36:02 PM

Electrophoresis File Run Summary

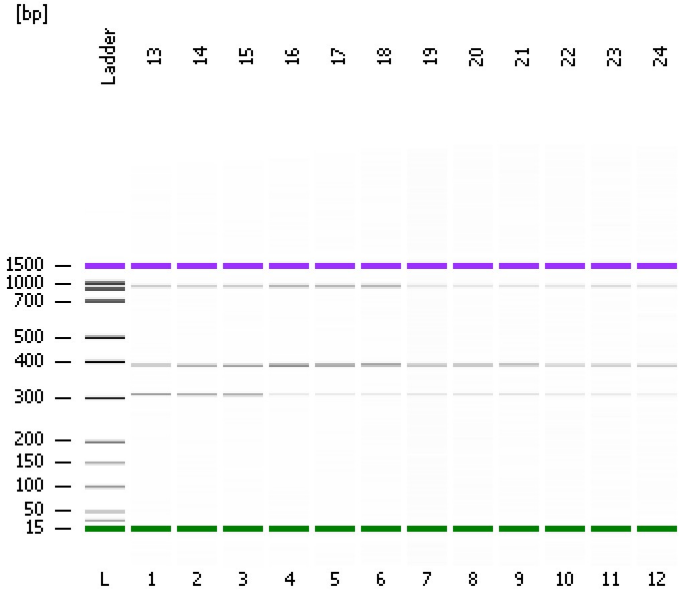

Instrument Information:

Instrument Name:	DE13804763	Firmware:	C.01.069
Serial#:	DE13804763	Type:	G2939A

Assay Information:

Assay Origin Path:	C:\Program Files\Agilent\2100 bioanalyzer\2100 expert\assays\dsDNA\DNA 1000 Series II.xsy
Assay Class:	DNA 1000
Version:	2.3
Assay Comments:	DNA Analysis 25 -1000 bp

© Copyright 2003-2009 Agilent Technologies, Inc.

Chip Information:

Chip Lot #:

Reagent Kit Lot #:

Chip Comments:

13

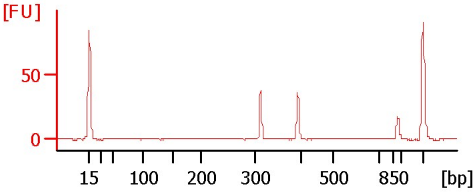

14

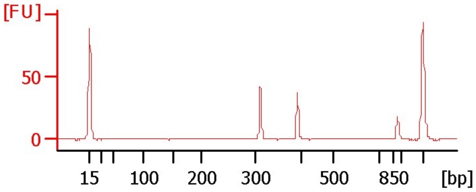

15

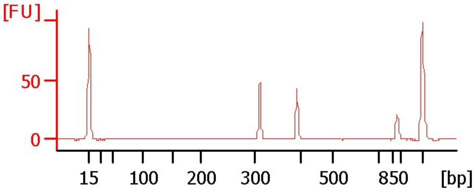

16

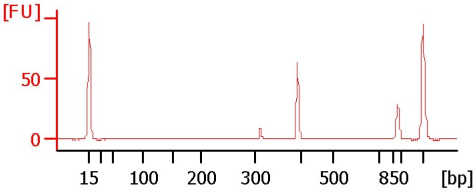

17

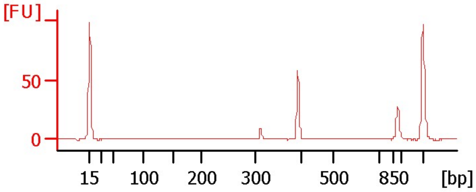

18

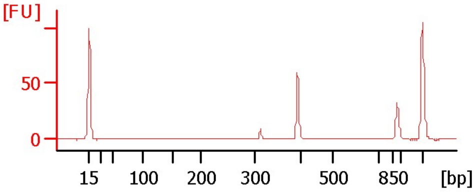

19

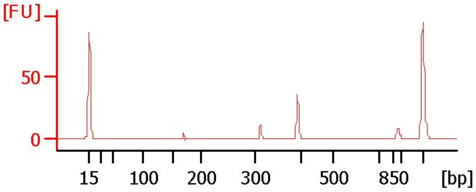

20

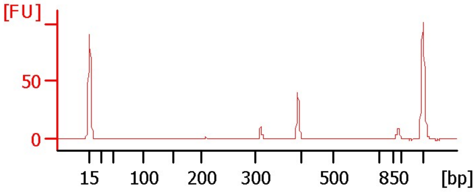

21

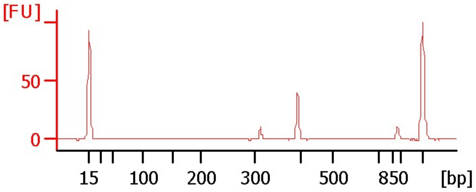

22

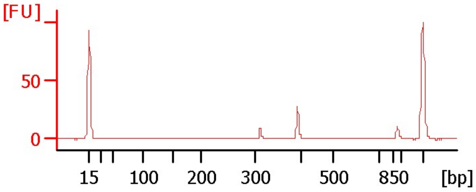

23

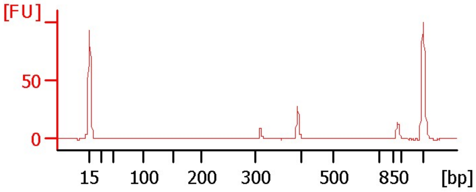

24

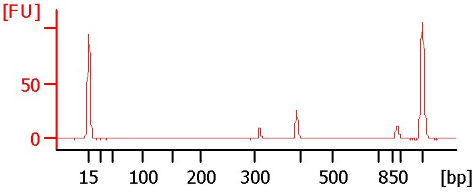

 © Copyright 2003 - 2009 Agilent Technologies, Inc.

Assay Class: DNA 1000
Data Path: C:\...-26\2100 expert_DNA 1000_DE13804763_2023-04-26_13-53-45.xad
Created: 4/26/2023 1:53:44 PM
Modified: 4/26/2023 2:36:02 PM

Electrophoresis File Run Summary (Chip Summary)

Sample Name	Sample Comment	Rest. Digest	Status	Observation	Result Label	Result Color
13		☐	✔			
14		☐	✔			
15		☐	✔			
16		☐	✔			
17		☐	✔			
18		☐	✔			
19		☐	✔			
20		☐	✔			
21		☐	✔			
22		☐	✔			
23		☐	✔			
24		☐	✔			
Ladder		☐	✔			

Chip Lot # **Reagent Kit Lot #**

Chip Comments :

© Copyright 2003 - 2009 Agilent Technologies, Inc.

Assay Class:	DNA 1000	Created:	4/26/2023 1:53:44 PM
Data Path:	C:\...-26\2100 expert_DNA 1000_DE13804763_2023-04-26_13-53-45.xad	Modified:	4/26/2023 2:36:02 PM

Electrophoresis Assay Details

General Analysis Settings

Number of Available Sample and Ladder Wells (Max.) : 13
Minimum Visible Range [s] : 30
Maximum Visible Range [s] : 129
Start Analysis Time Range [s] : 30
End Analysis Time Range [s] : 128.95
Ladder Concentration [ng/µl] : 44
Uses Standard Area for Ladder Fragments
Lower Marker Concentration [ng/µl] : 4.2
Upper Marker Concentration [ng/µl] : 2.1
Used Upper Marker for Quantitation
Standard Curve Fit is Point to Point
Show Data Aligned to Lower and Upper Marker

Integrator Settings

Integration Start Time [s] : 30
Integration End Time [s] : 128.95
Slope Threshold : 0.5
Height Threshold [FU] : 1
Area Threshold : 0.1
Width Threshold [s] : 0.5
Baseline Plateau [s] : 0.5

Filter Settings

Filter Width [s] : 0.5
Polynomial Order : 4

Ladder

Ladder Peak	Size	Area
1	15	25
2	25	26
3	50	34
4	100	41
5	150	45
6	200	52
7	300	63
8	400	76
9	500	83
10	700	88
11	850	86
12	1000	90
13	1500	52

 © Copyright 2003 - 2009 Agilent Technologies, Inc.

Assay Class:	DNA 1000	Created:	4/26/2023 1:53:44 PM
Data Path:	C:\...-26\2100 expert_DNA 1000_DE13804763_2023-04-26_13-53-45.xad	Modified:	4/26/2023 2:36:02 PM

Electropherogram Summary

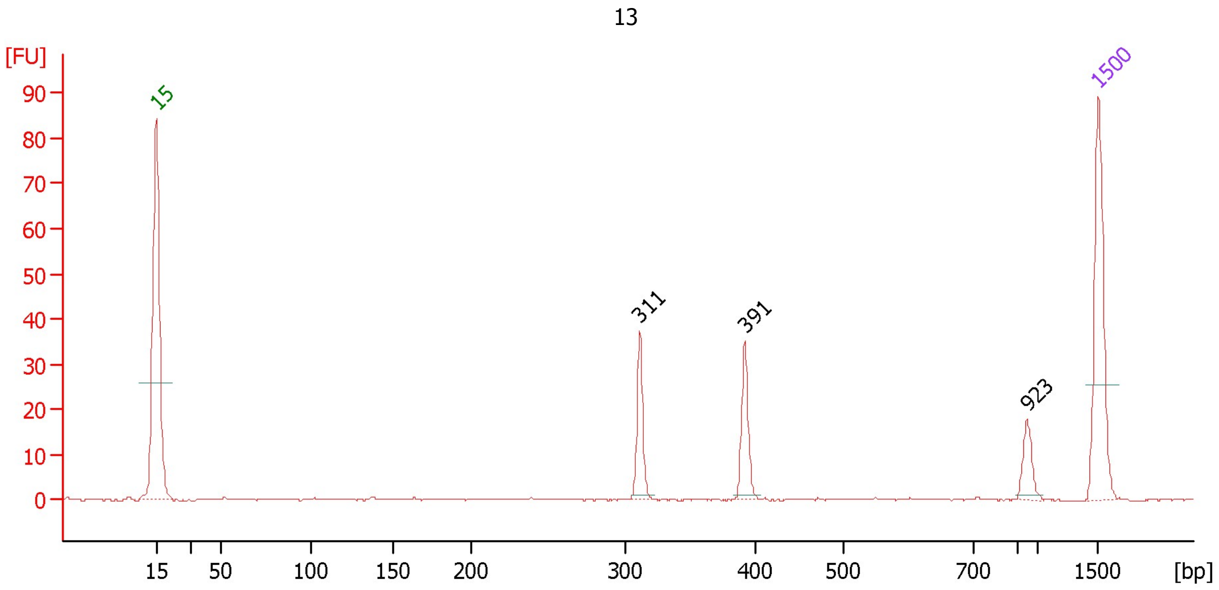

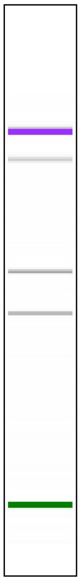

Overall Results for sample 1 : 13

Number of peaks found: 3

Peak table for sample 1 : 13

Peak		Size [bp]	Conc. [ng/µl]	Molarity [nmol/l]	Observations
1	◀	15	4.20	424.2	Lower Marker
2		311	0.86	4.2	
3		391	0.78	3.0	
4		923	0.46	0.8	
5	▶	1,500	2.10	2.1	Upper Marker

 © Copyright 2003 - 2009 Agilent Technologies, Inc.

Assay Class: DNA 1000
Data Path: C:\...-26\2100 expert_DNA 1000_DE13804763_2023-04-26_13-53-45.xad

Created: 4/26/2023 1:53:44 PM
Modified: 4/26/2023 2:36:02 PM

Electropherogram Summary Continued ...

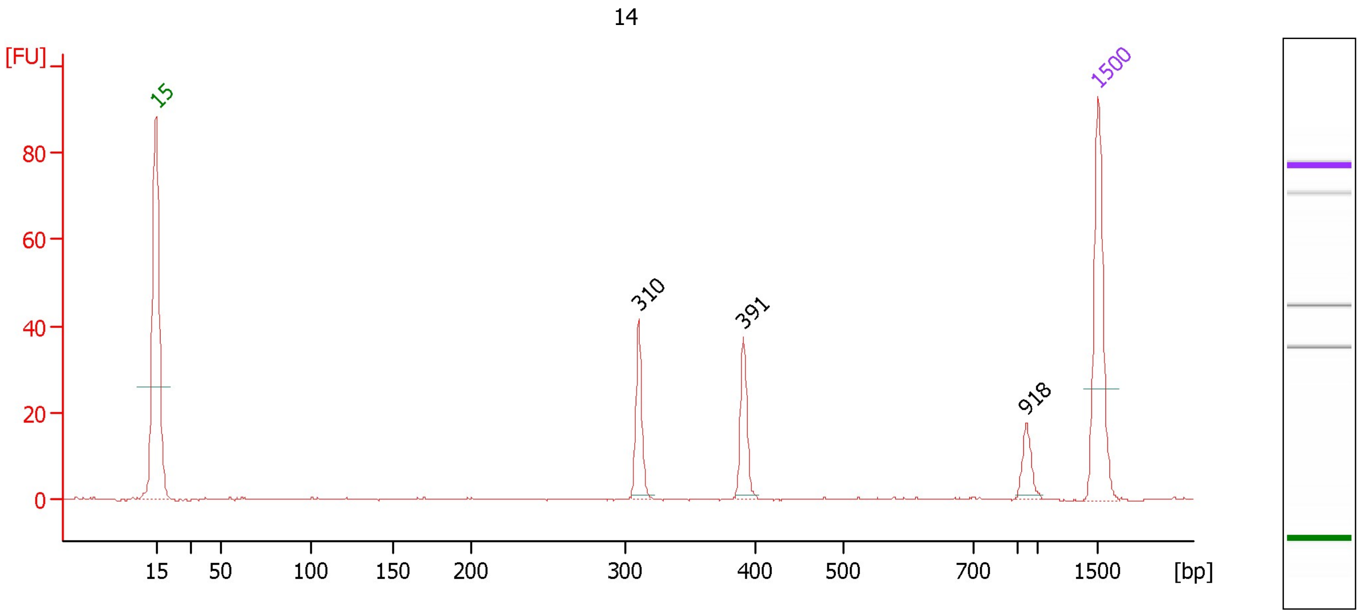

Overall Results for sample 2 : 14

Number of peaks found: 3

Peak table for sample 2 : 14

Peak		Size [bp]	Conc. [ng/µl]	Molarity [nmol/l]	Observations
1	◀	15	4.20	424.2	Lower Marker
2		310	0.93	4.5	
3		391	0.82	3.2	
4		918	0.44	0.7	
5	▶	1,500	2.10	2.1	Upper Marker

 © Copyright 2003 - 2009 Agilent Technologies, Inc.

Assay Class: DNA 1000
Data Path: C:\...-26\2100 expert_DNA 1000_DE13804763_2023-04-26_13-53-45.xad
Created: 4/26/2023 1:53:44 PM
Modified: 4/26/2023 2:36:02 PM

Electropherogram Summary Continued ...

15

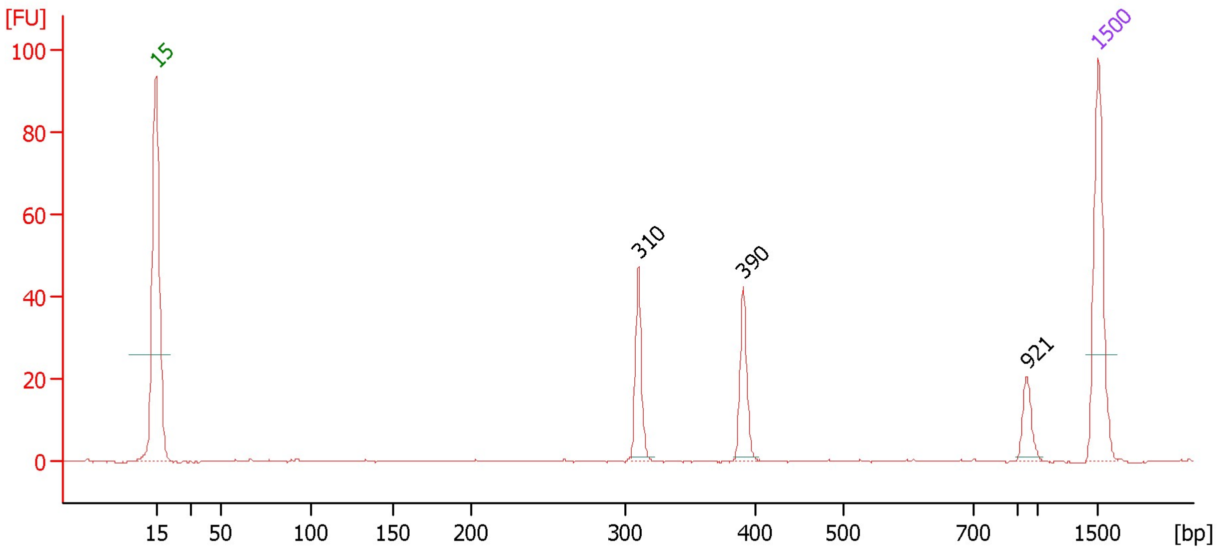

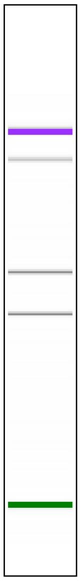

Overall Results for sample 3 : 15

Number of peaks found: 3

Peak table for sample 3 : 15

Peak		Size [bp]	Conc. [ng/µl]	Molarity [nmol/l]	Observations
1	◀	15	4.20	424.2	Lower Marker
2		310	1.04	5.1	
3		390	0.91	3.5	
4		921	0.48	0.8	
5	▶	1,500	2.10	2.1	Upper Marker

 © Copyright 2003 - 2009 Agilent Technologies, Inc.

Assay Class: DNA 1000 Created: 4/26/2023 1:53:44 PM
Data Path: C:\...-26\2100 expert_DNA 1000_DE13804763_2023-04-26_13-53-45.xad Modified: 4/26/2023 2:36:02 PM

Electropherogram Summary Continued ...

16

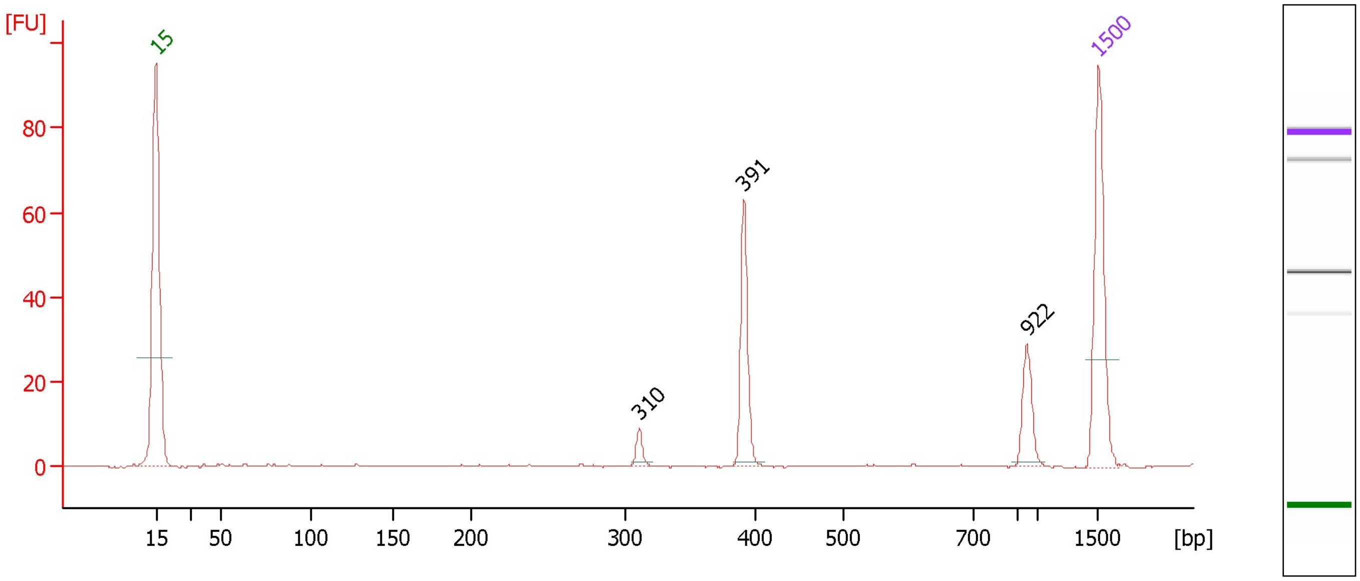

Overall Results for sample 4 : 16

Number of peaks found: 3

Peak table for sample 4 : 16

Peak		Size [bp]	Conc. [ng/µl]	Molarity [nmol/l]	Observations
1	◀	15	4.20	424.2	Lower Marker
2		310	0.20	1.0	
3		391	1.35	5.2	
4		922	0.72	1.2	
5	▶	1,500	2.10	2.1	Upper Marker

 © Copyright 2003 - 2009 Agilent Technologies, Inc.

Assay Class:	DNA 1000	Created:	4/26/2023 1:53:44 PM
Data Path:	C:\...-26\2100 expert_DNA 1000_DE13804763_2023-04-26_13-53-45.xad	Modified:	4/26/2023 2:36:02 PM

Electropherogram Summary Continued ...

17

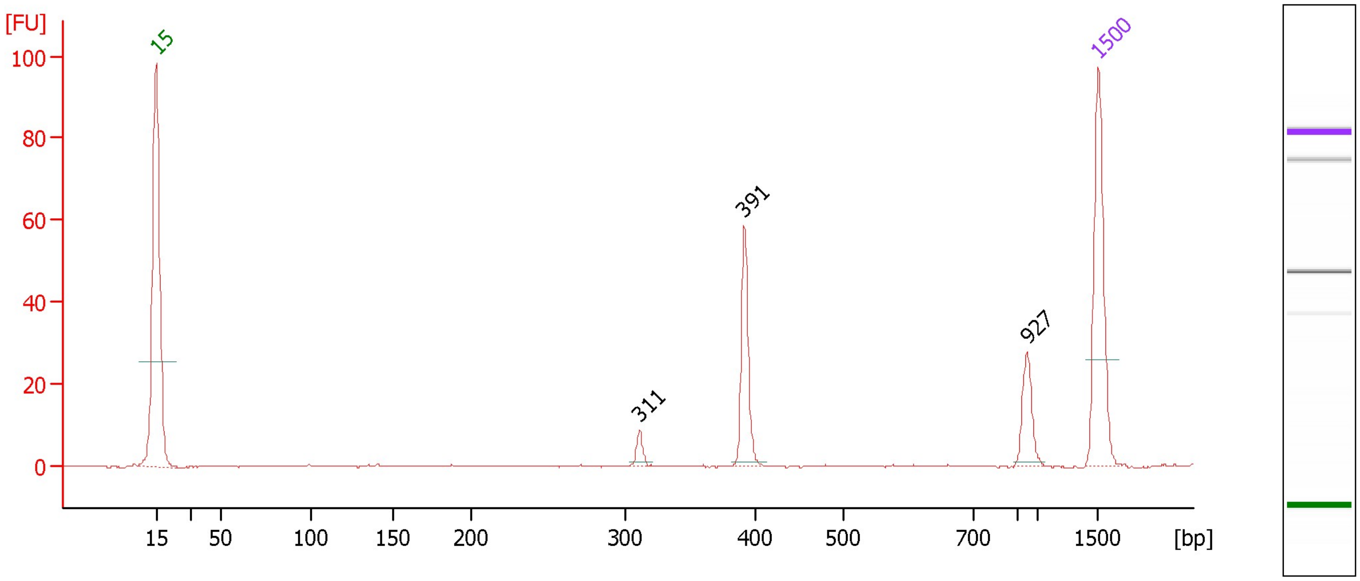

Overall Results for sample 5 : 17

Number of peaks found: 3

Peak table for sample 5 : 17

Peak		Size [bp]	Conc. [ng/µl]	Molarity [nmol/l]	Observations
1	◀	15	4.20	424.2	Lower Marker
2		311	0.19	0.9	
3		391	1.24	4.8	
4		927	0.68	1.1	
5	▶	1,500	2.10	2.1	Upper Marker

 © Copyright 2003 - 2009 Agilent Technologies, Inc.

Assay Class: DNA 1000 Created: 4/26/2023 1:53:44 PM
Data Path: C:\...-26\2100 expert_DNA 1000_DE13804763_2023-04-26_13-53-45.xad Modified: 4/26/2023 2:36:02 PM

Electropherogram Summary Continued ...

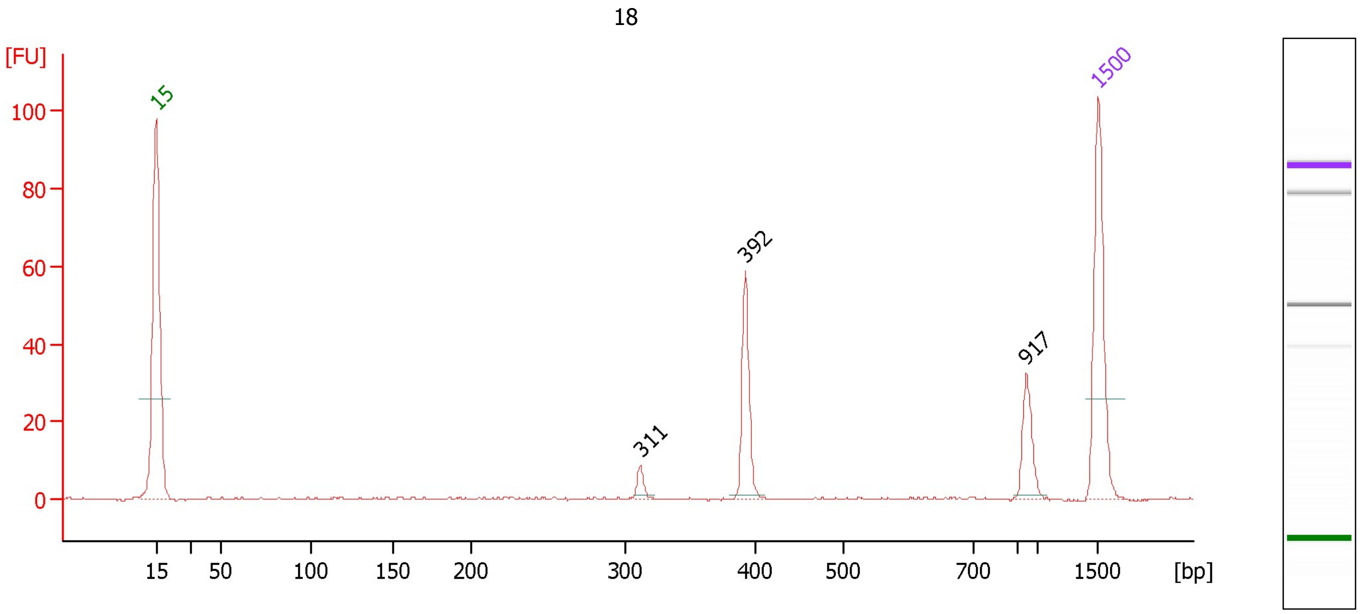

Overall Results for sample 6 : 18

Number of peaks found: 3

Peak table for sample 6 : 18

Peak		Size [bp]	Conc. [ng/µl]	Molarity [nmol/l]	Observations
1	◀	15	4.20	424.2	Lower Marker
2		311	0.18	0.9	
3		392	1.18	4.6	
4		917	0.72	1.2	
5	▶	1,500	2.10	2.1	Upper Marker

 © Copyright 2003 - 2009 Agilent Technologies, Inc.

Assay Class: DNA 1000
Data Path: C:\...-26\2100 expert_DNA 1000_DE13804763_2023-04-26_13-53-45.xad

Created: 4/26/2023 1:53:44 PM
Modified: 4/26/2023 2:36:02 PM

Electropherogram Summary Continued ...

19

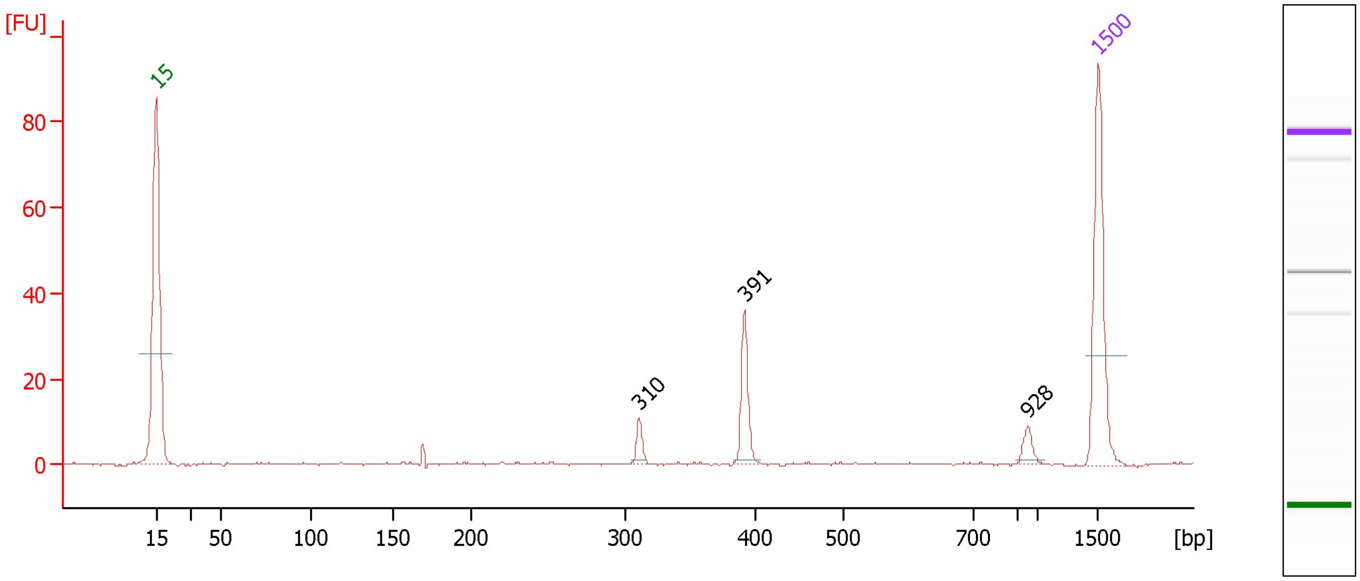

Overall Results for sample 7 : 19

Number of peaks found: 3

Peak table for sample 7 : 19

Peak		Size [bp]	Conc. [ng/µl]	Molarity [nmol/l]	Observations
1	◀	15	4.20	424.2	Lower Marker
2		310	0.22	1.1	
3		391	0.76	2.9	
4		928	0.22	0.4	
5	▶	1,500	2.10	2.1	Upper Marker

 © Copyright 2003 - 2009 Agilent Technologies, Inc.

Assay Class: DNA 1000 Created: 4/26/2023 1:53:44 PM
Data Path: C:\...-26\2100 expert_DNA 1000_DE13804763_2023-04-26_13-53-45.xad Modified: 4/26/2023 2:36:02 PM

Electropherogram Summary Continued ...

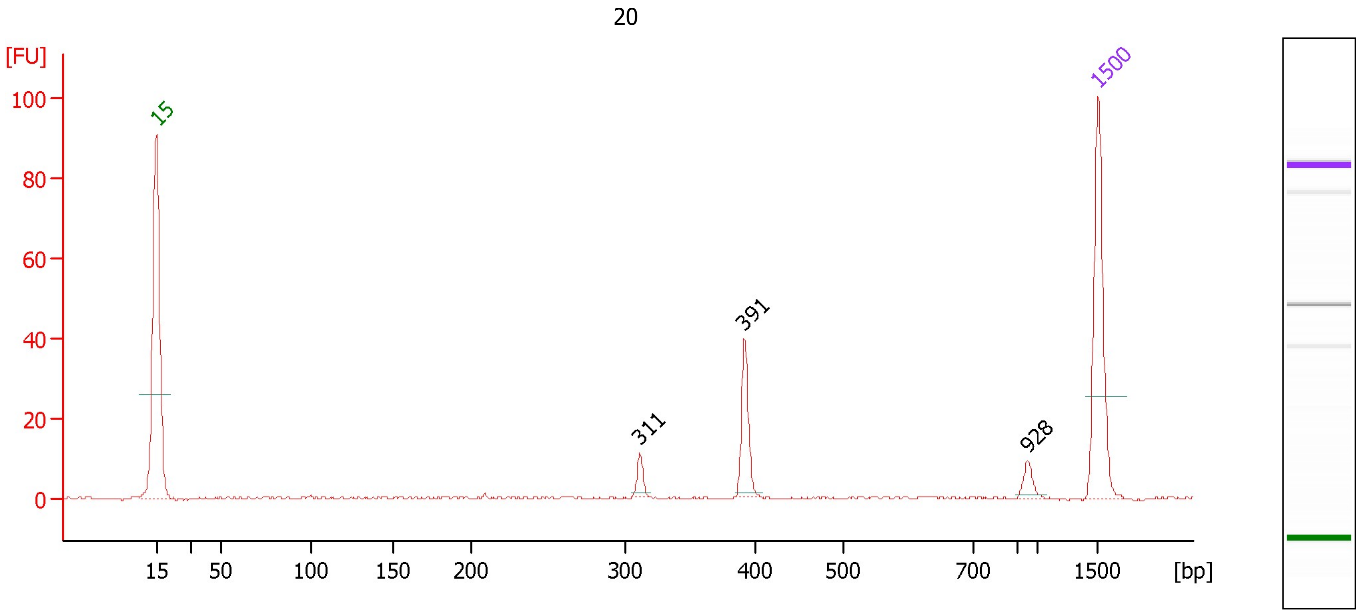

Overall Results for sample 8 : 20

Number of peaks found: 3

Peak table for sample 8 : 20

Peak		Size [bp]	Conc. [ng/µl]	Molarity [nmol/l]	Observations
1	◀	15	4.20	424.2	Lower Marker
2		311	0.24	1.2	
3		391	0.82	3.2	
4		928	0.23	0.4	
5	▶	1,500	2.10	2.1	Upper Marker

 © Copyright 2003 - 2009 Agilent Technologies, Inc.

Assay Class: DNA 1000
Data Path: C:\...-26\2100 expert_DNA 1000_DE13804763_2023-04-26_13-53-45.xad

Created: 4/26/2023 1:53:44 PM
Modified: 4/26/2023 2:36:02 PM

Electropherogram Summary Continued ...

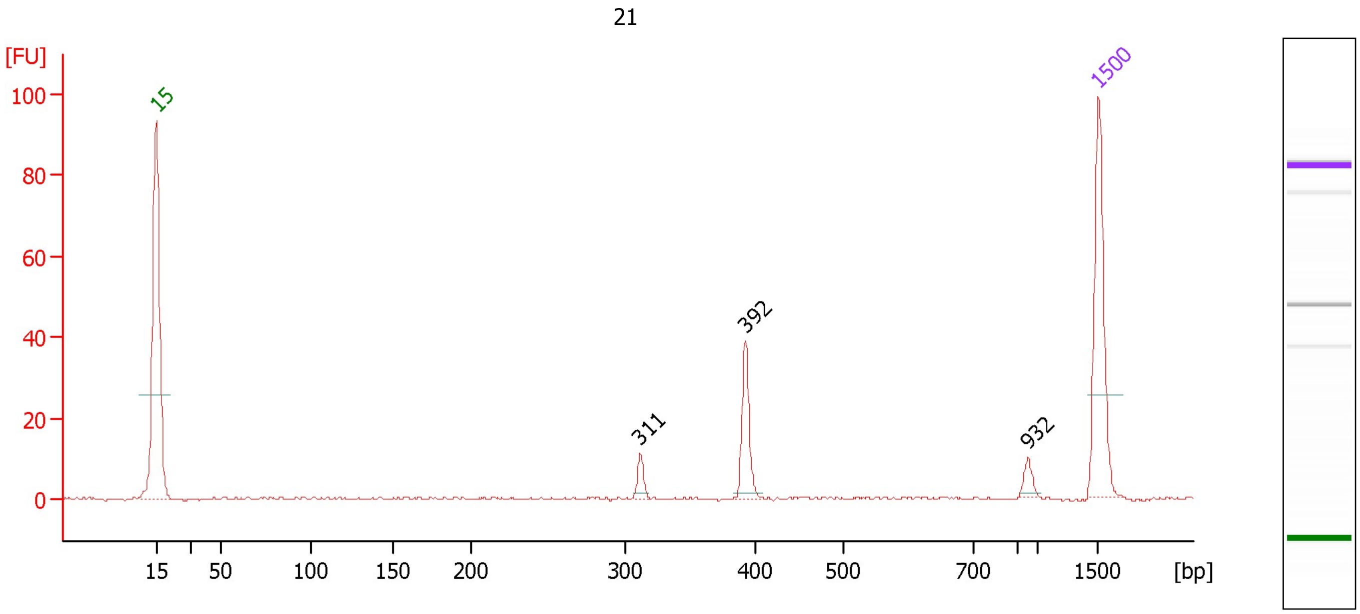

Overall Results for sample 9 : 21

Number of peaks found: 3

Peak table for sample 9 : 21

Peak		Size [bp]	Conc. [ng/µl]	Molarity [nmol/l]	Observations
1	◀	15	4.20	424.2	Lower Marker
2		311	0.23	1.1	
3		392	0.81	3.1	
4		932	0.21	0.3	
5	▶	1,500	2.10	2.1	Upper Marker

 © Copyright 2003 - 2009 Agilent Technologies, Inc.

Assay Class: DNA 1000
Data Path: C:\...-26\2100 expert_DNA 1000_DE13804763_2023-04-26_13-53-45.xad
Created: 4/26/2023 1:53:44 PM
Modified: 4/26/2023 2:36:02 PM

Electropherogram Summary Continued ...

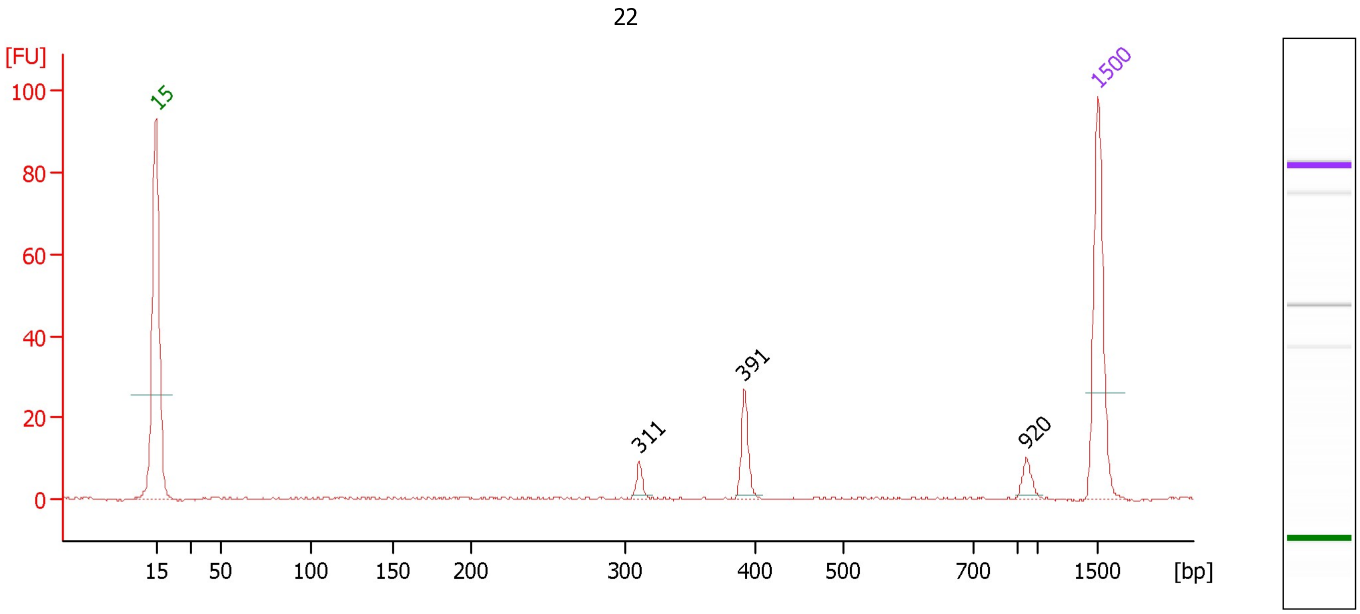

Overall Results for sample 10 : 22

Number of peaks found: 3

Peak table for sample 10 : 22

Peak	Size [bp]	Conc. [ng/µl]	Molarity [nmol/l]	Observations
1	15	4.20	424.2	Lower Marker
2	311	0.19	0.9	
3	391	0.54	2.1	
4	920	0.23	0.4	
5	1,500	2.10	2.1	Upper Marker

© Copyright 2003 - 2009 Agilent Technologies, Inc.

Assay Class: DNA 1000
Data Path: C:\...-26\2100 expert_DNA 1000_DE13804763_2023-04-26_13-53-45.xad

Created: 4/26/2023 1:53:44 PM
Modified: 4/26/2023 2:36:02 PM

Electropherogram Summary Continued ...

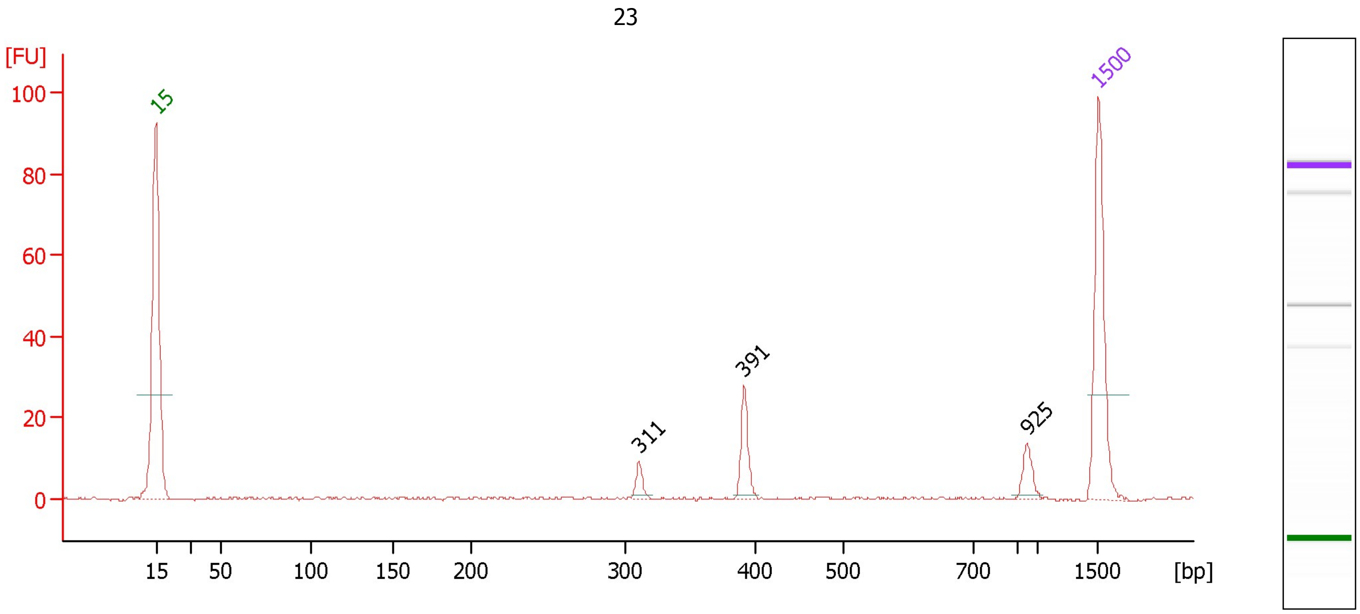

Overall Results for sample 11 : 23

Number of peaks found: 3

Peak table for sample 11 : 23

Peak		Size [bp]	Conc. [ng/µl]	Molarity [nmol/l]	Observations
1	◀	15	4.20	424.2	Lower Marker
2		311	0.21	1.0	
3		391	0.58	2.2	
4		925	0.33	0.5	
5	▶	1,500	2.10	2.1	Upper Marker

© Copyright 2003 - 2009 Agilent Technologies, Inc.

Assay Class: DNA 1000
Data Path: C:\...-26\2100 expert_DNA 1000_DE13804763_2023-04-26_13-53-45.xad

Created: 4/26/2023 1:53:44 PM
Modified: 4/26/2023 2:36:02 PM

Electropherogram Summary Continued ...

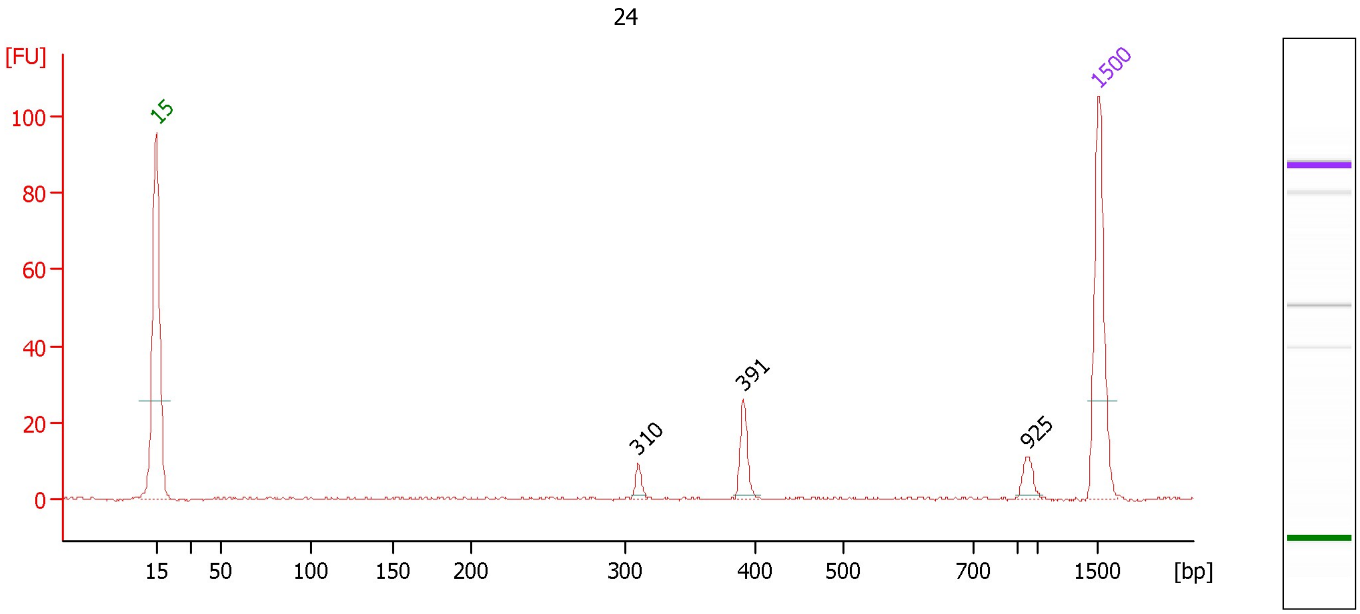

Overall Results for sample 12 : 24

Number of peaks found: 3

Peak table for sample 12 : 24

Peak		Size [bp]	Conc. [ng/µl]	Molarity [nmol/l]	Observations
1	◀	15	4.20	424.2	Lower Marker
2		310	0.17	0.9	
3		391	0.52	2.0	
4		925	0.26	0.4	
5	▶	1,500	2.10	2.1	Upper Marker

 © Copyright 2003 - 2009 Agilent Technologies, Inc.

Assay Class: DNA 1000
Data Path: C:\...-26\2100 expert_DNA 1000_DE13804763_2023-04-26_13-53-45.xad
Created: 4/26/2023 1:53:44 PM
Modified: 4/26/2023 2:36:02 PM

Gel Image

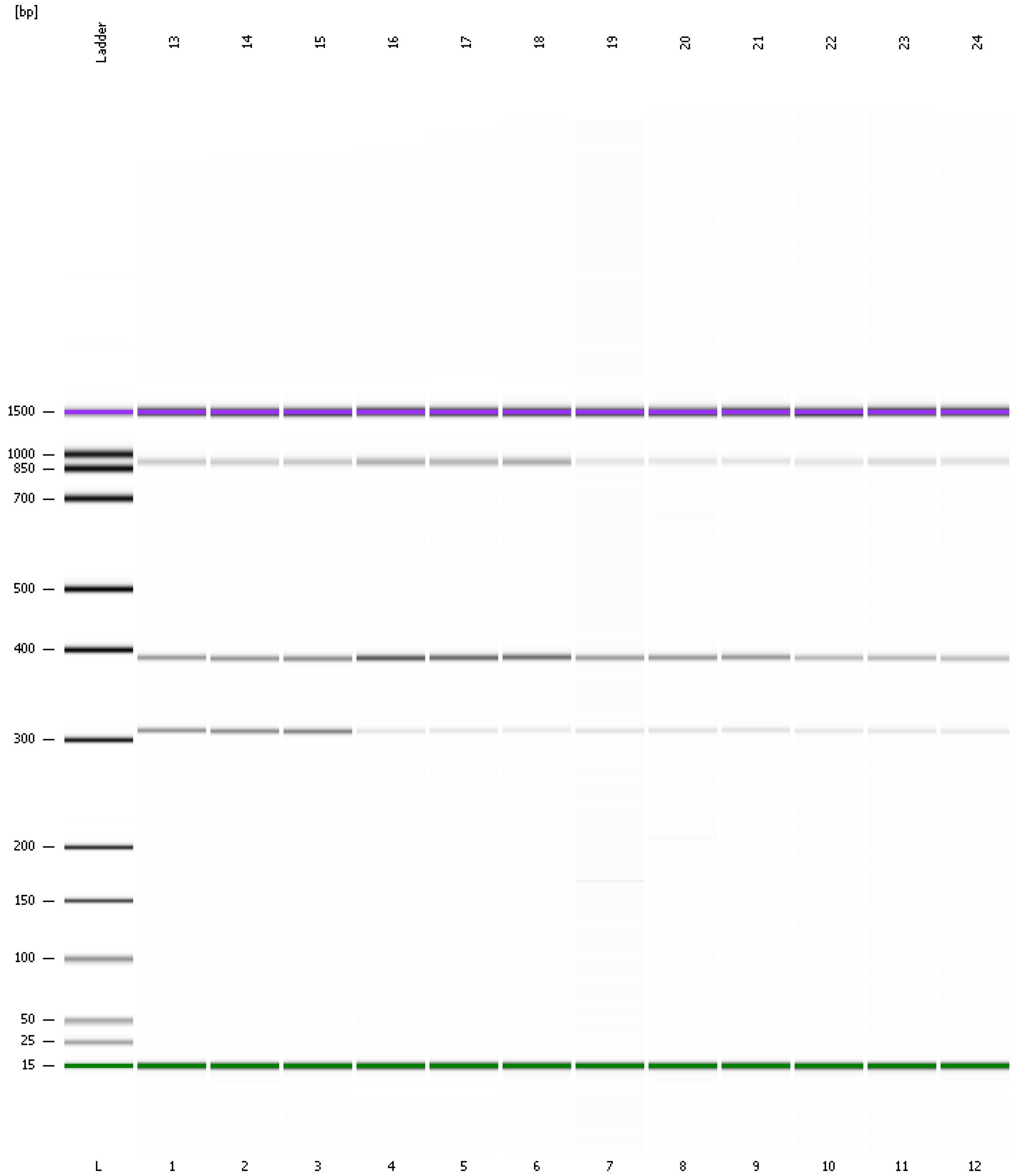

 © Copyright 2003 - 2009 Agilent Technologies, Inc.
