# Supplementary material for: Competition for the conserved branch point sequence influences physiological outcomes in pre-mRNA splicing
Source: eLife. 2026 Mar 20;13:RP103167. doi: 10.7554/eLife.103167 (PMC13004596; doi:10.7554/eLife.103167)
Supplement: Figure 3—source data 2. [file elife-103167-fig3-data2.zip › Fig3/112922_DUP-RAI14-DelMTs_C2C12_BioAnalyzer.pdf]

Assay Class: DNA 1000
Data Path: C:\...-29\2100 expert_DNA 1000_DE13804763_2022-11-29_12-51-34.xad

Created: 11/29/2022 12:51:33 PM
Modified: 11/29/2022 1:34:25 PM

Electrophoresis File Run Summary

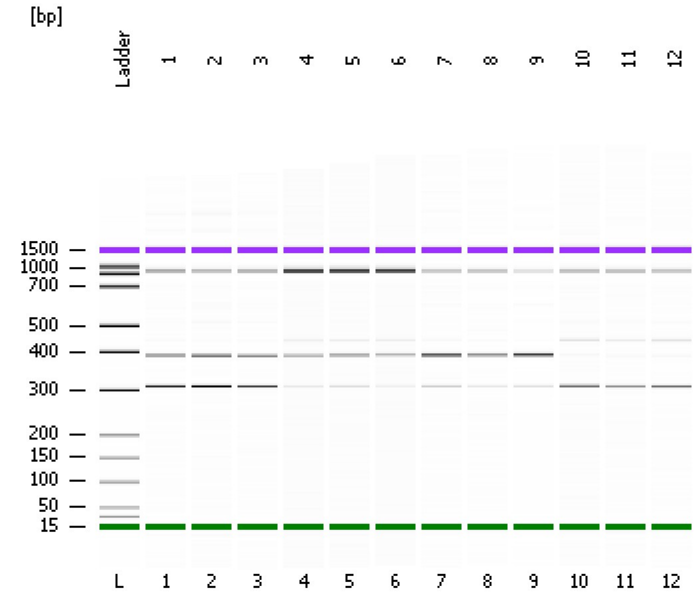

Instrument Information:

Instrument Name: DE13804763
Firmware: C.01.069
Serial#: DE13804763
Type: G2939A

Assay Information:

Assay Origin Path: C:\Program Files\Agilent\2100 bioanalyzer\2100 expert\assays\dsDNA\DNA 1000 Series II.xsy

Assay Class: DNA 1000

Version: 2.3

Assay Comments: DNA Analysis 25 -1000 bp

© Copyright 2003-2009 Agilent Technologies, Inc.

Chip Information:

Chip Lot #:

Reagent Kit Lot #:

Chip Comments:

1

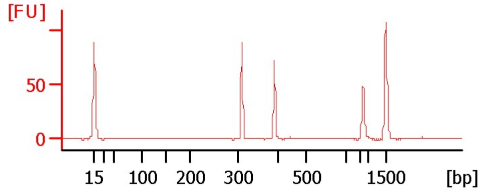

2

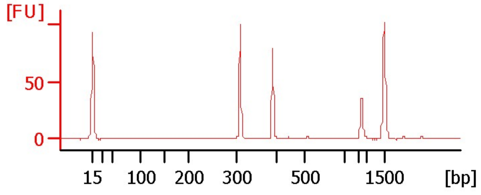

3

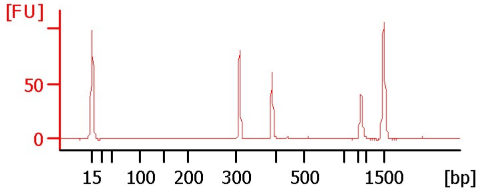

4

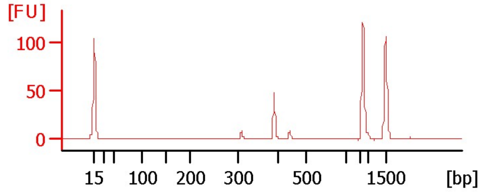

5

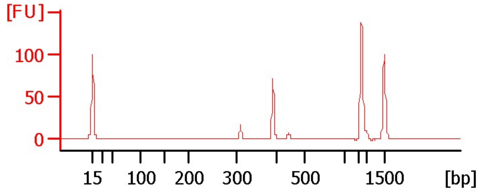

6

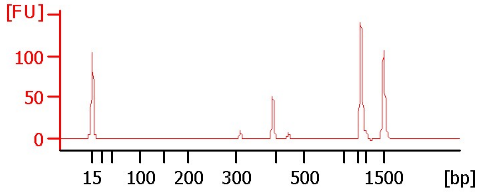

7

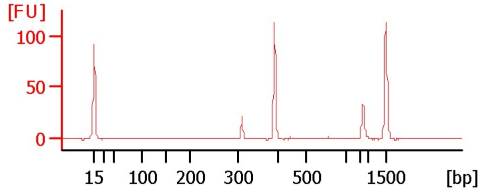

8

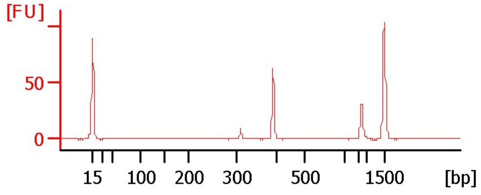

9

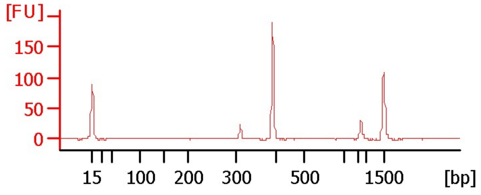

10

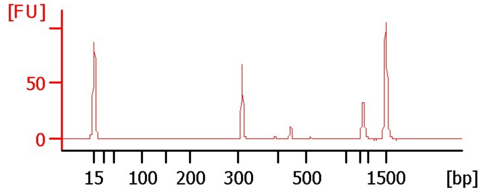

11

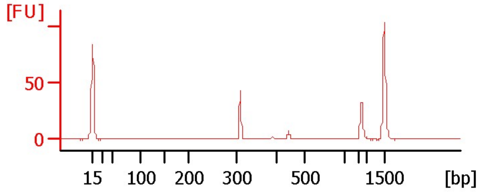

12

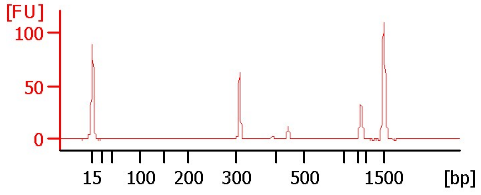

Assay Class: DNA 1000
Data Path: C:\...-29\2100 expert_DNA 1000_DE13804763_2022-11-29_12-51-34.xad

Created: 11/29/2022 12:51:33 PM
Modified: 11/29/2022 1:34:25 PM

Electrophoresis File Run Summary (Chip Summary)

Sample Name	Sample Comment	Rest. Digest	Status	Observation	Result Label	Result Color
1		☐	✔			
2		☐	✔			
3		☐	✔			
4		☐	✔			
5		☐	✔			
6		☐	✔			
7		☐	✔			
8		☐	✔			
9		☐	✔			
10		☐	✔			
11		☐	✔			
12		☐	✔			
Ladder		☐	✔			

Chip Lot # **Reagent Kit Lot #**

Chip Comments :

© Copyright 2003 - 2009 Agilent Technologies, Inc.

Assay Class: DNA 1000
Data Path: C:\...-29\2100 expert_DNA 1000_DE13804763_2022-11-29_12-51-34.xad
Created: 11/29/2022 12:51:33 PM
Modified: 11/29/2022 1:34:25 PM

Electrophoresis Assay Details

General Analysis Settings

Number of Available Sample and Ladder Wells (Max.) : 13
Minimum Visible Range [s] : 30
Maximum Visible Range [s] : 129
Start Analysis Time Range [s] : 30
End Analysis Time Range [s] : 128.95
Ladder Concentration [ng/µl] : 44
Uses Standard Area for Ladder Fragments
Lower Marker Concentration [ng/µl] : 4.2
Upper Marker Concentration [ng/µl] : 2.1
Used Upper Marker for Quantitation
Standard Curve Fit is Point to Point
Show Data Aligned to Lower and Upper Marker

Integrator Settings

Integration Start Time [s] : 30
Integration End Time [s] : 128.95
Slope Threshold : 0.5
Height Threshold [FU] : 1
Area Threshold : 0.1
Width Threshold [s] : 0.5
Baseline Plateau [s] : 0.5

Filter Settings

Filter Width [s] : 0.5
Polynomial Order : 4

Ladder

Ladder Peak	Size	Area
1	15	25
2	25	26
3	50	34
4	100	41
5	150	45
6	200	52
7	300	63
8	400	76
9	500	83
10	700	88
11	850	86
12	1000	90
13	1500	52

© Copyright 2003 - 2009 Agilent Technologies, Inc.

Assay Class: DNA 1000
Data Path: C:\...-29\2100 expert_DNA 1000_DE13804763_2022-11-29_12-51-34.xad

Created: 11/29/2022 12:51:33 PM
Modified: 11/29/2022 1:34:25 PM

Electropherogram Summary

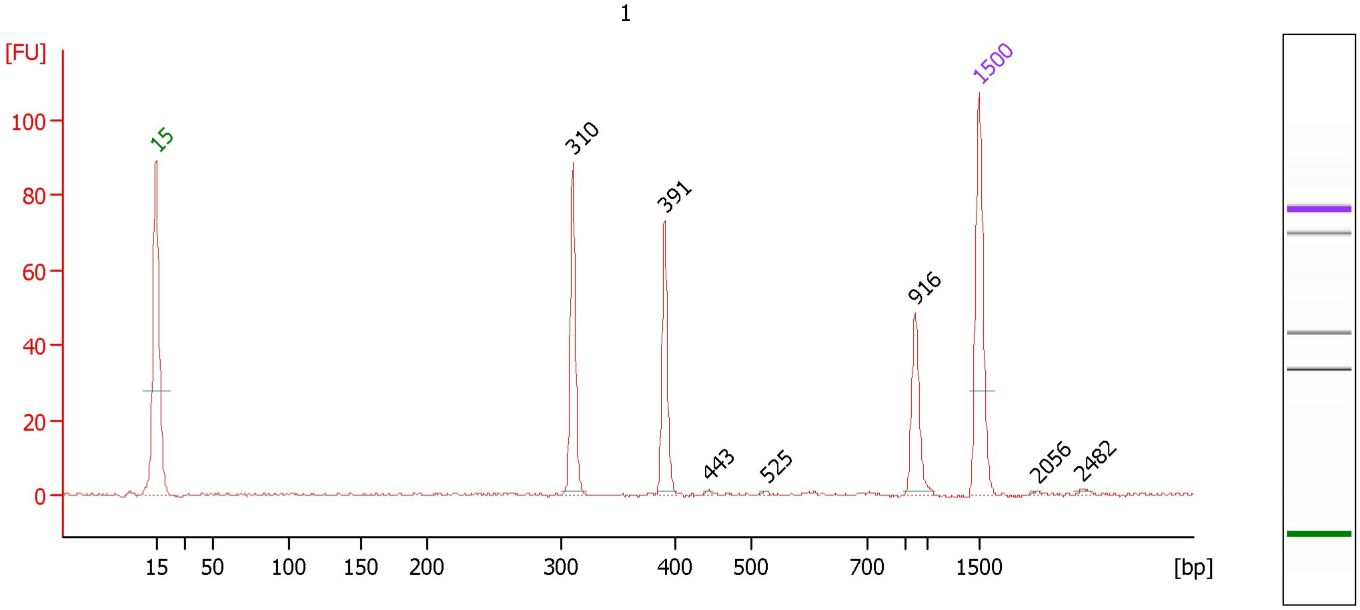

Overall Results for sample 1 : 1

Number of peaks found: 5

Peak table for sample 1 : 1

Peak		Size [bp]	Conc. [ng/µl]	Molarity [nmol/l]	Observations
1	◀	15	4.20	424.2	Lower Marker
2		310	1.79	8.7	
3		391	1.36	5.3	
4		443	0.02	0.1	
5		525	0.02	0.0	
6		916	1.08	1.8	
7	▶	1,500	2.10	2.1	Upper Marker
8		2,056	0.00	0.0	
9		2,482	0.00	0.0	

 © Copyright 2003 - 2009 Agilent Technologies, Inc.

Assay Class: DNA 1000
Data Path: C:\...-29\2100 expert_DNA 1000_DE13804763_2022-11-29_12-51-34.xad

Created: 11/29/2022 12:51:33 PM
Modified: 11/29/2022 1:34:25 PM

Electropherogram Summary Continued ...

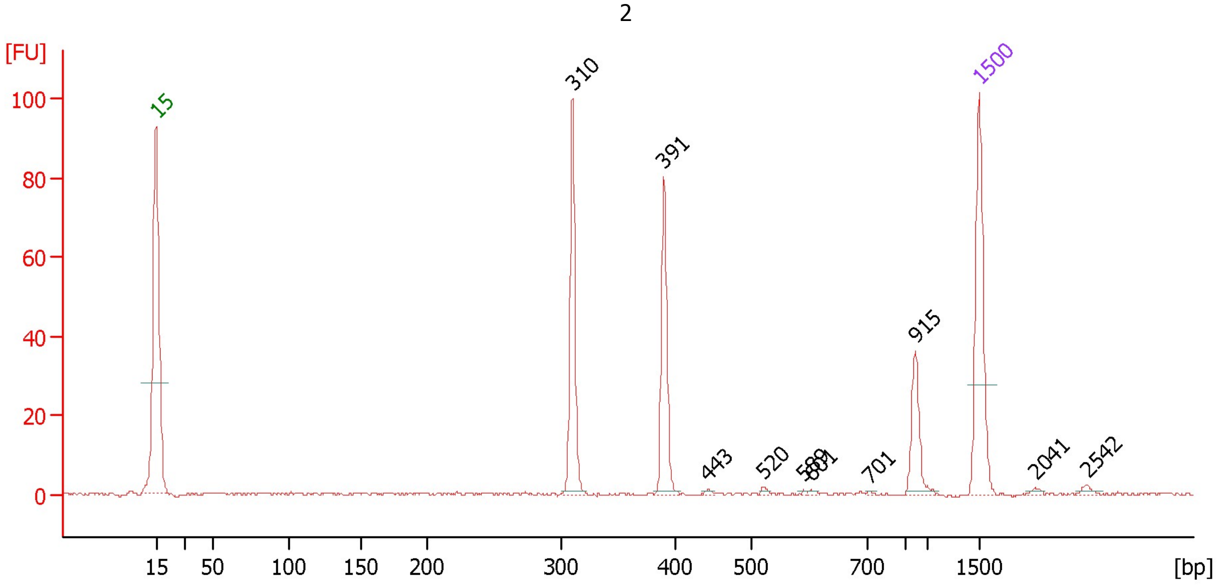

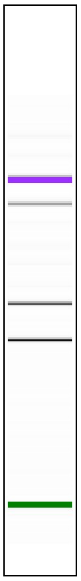

Overall Results for sample 2 : 2

Number of peaks found: 8

Peak table for sample 2 : 2

Peak		Size [bp]	Conc. [ng/µl]	Molarity [nmol/l]	Observations
1	◀	15	4.20	424.2	Lower Marker
2		310	2.09	10.2	
3		391	1.61	6.3	
4		443	0.03	0.1	
5		520	0.04	0.1	
6		589	0.02	0.0	
7		601	0.03	0.1	
8		701	0.02	0.0	
9		915	0.87	1.4	
10	▶	1,500	2.10	2.1	Upper Marker
11		2,041	0.00	0.0	
12		2,542	0.00	0.0	

 © Copyright 2003 - 2009 Agilent Technologies, Inc.

Assay Class: DNA 1000
Data Path: C:\...-29\2100 expert_DNA 1000_DE13804763_2022-11-29_12-51-34.xad

Created: 11/29/2022 12:51:33 PM
Modified: 11/29/2022 1:34:25 PM

Electropherogram Summary Continued ...

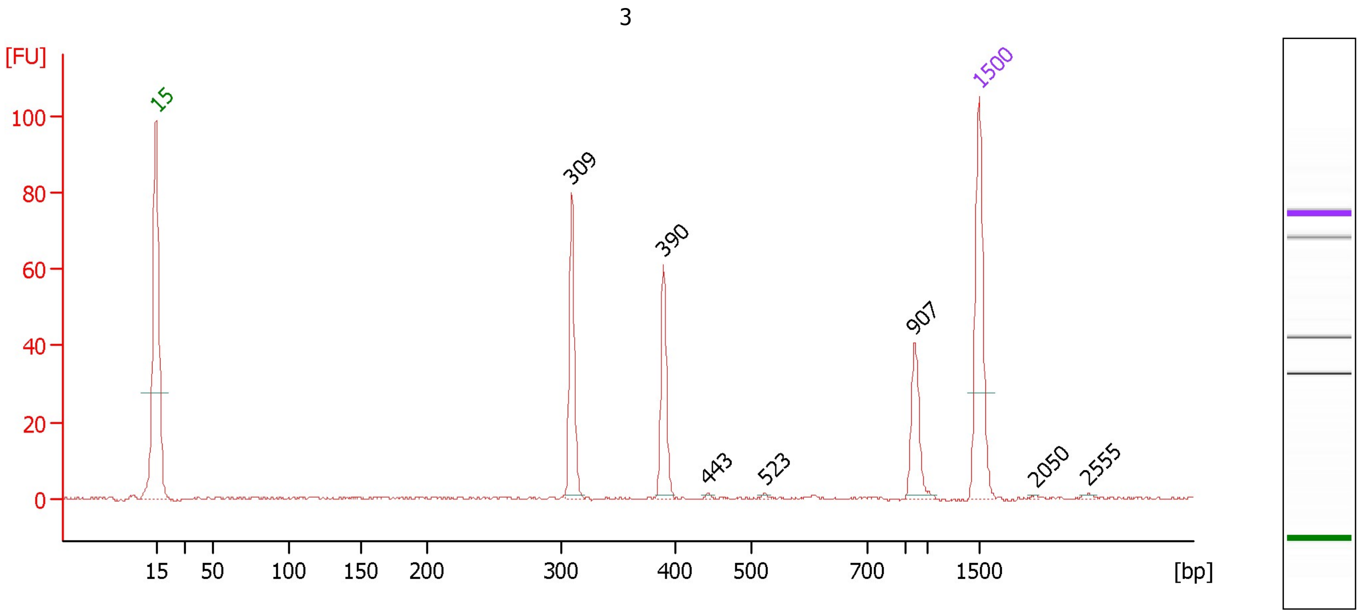

Overall Results for sample 3 : 3

Number of peaks found: 5

Peak table for sample 3 : 3

Peak		Size [bp]	Conc. [ng/µl]	Molarity [nmol/l]	Observations
1	◀	15	4.20	424.2	Lower Marker
2		309	1.56	7.7	
3		390	1.16	4.5	
4		443	0.03	0.1	
5		523	0.03	0.1	
6		907	0.95	1.6	
7	▶	1,500	2.10	2.1	Upper Marker
8		2,050	0.00	0.0	
9		2,555	0.00	0.0	

© Copyright 2003 - 2009 Agilent Technologies, Inc.

Assay Class: DNA 1000
Data Path: C:\...-29\2100 expert_DNA 1000_DE13804763_2022-11-29_12-51-34.xad

Created: 11/29/2022 12:51:33 PM
Modified: 11/29/2022 1:34:25 PM

Electropherogram Summary Continued ...

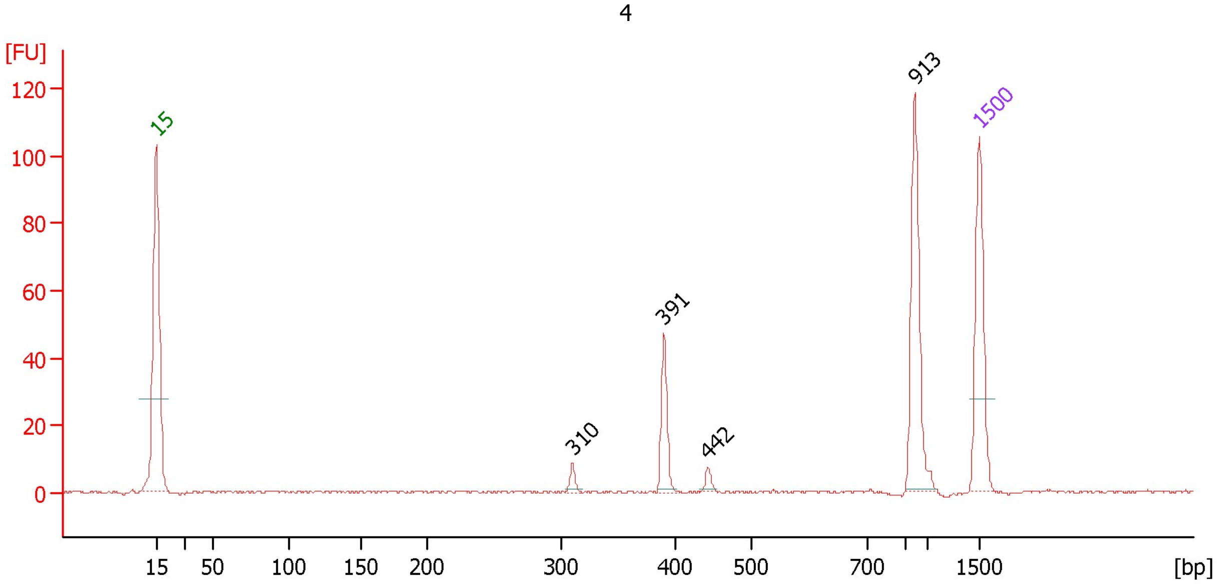

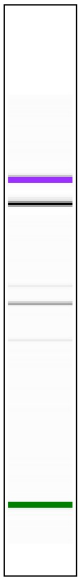

Overall Results for sample 4 : 4

Number of peaks found: 4

Peak table for sample 4 : 4

Peak		Size [bp]	Conc. [ng/µl]	Molarity [nmol/l]	Observations
1	◀	15	4.20	424.2	Lower Marker
2		310	0.16	0.8	
3		391	0.90	3.5	
4		442	0.13	0.4	
5		913	2.71	4.5	
6	▶	1,500	2.10	2.1	Upper Marker

 © Copyright 2003 - 2009 Agilent Technologies, Inc.

Assay Class: DNA 1000
Data Path: C:\...-29\2100 expert_DNA 1000_DE13804763_2022-11-29_12-51-34.xad

Created: 11/29/2022 12:51:33 PM
Modified: 11/29/2022 1:34:25 PM

Electropherogram Summary Continued ...

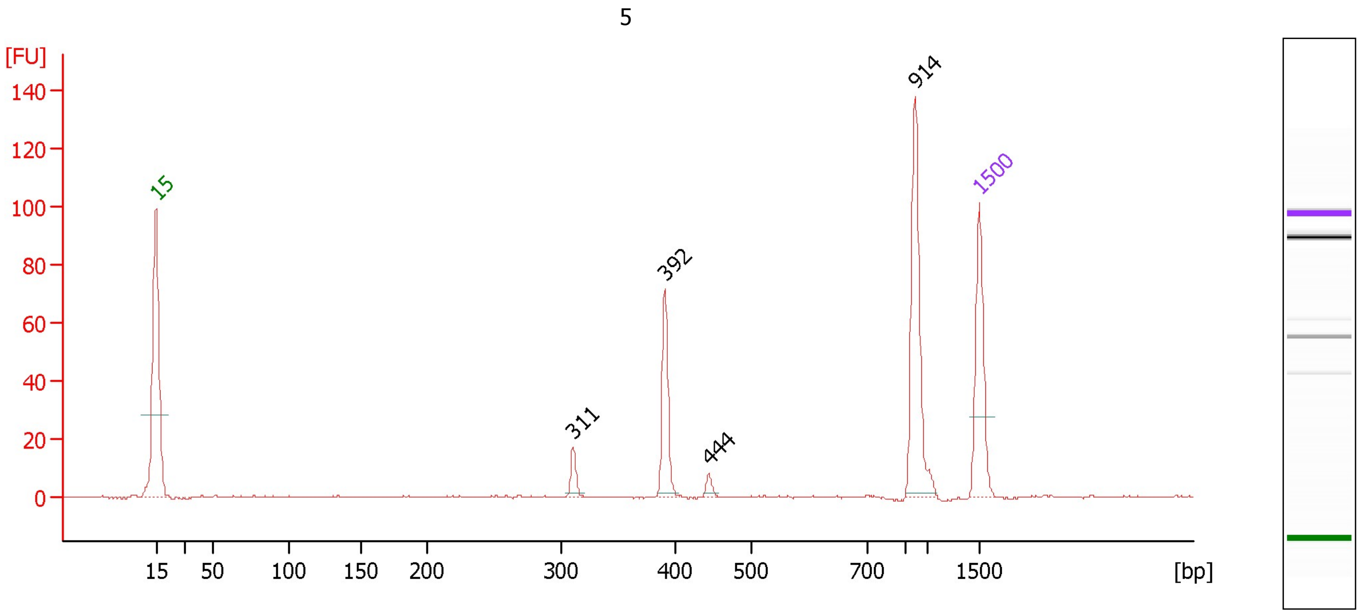

Overall Results for sample 5 : **5**

Number of peaks found: 4

Peak table for sample 5 : **5**

Peak		Size [bp]	Conc. [ng/µl]	Molarity [nmol/l]	Observations
1	◀	15	4.20	424.2	Lower Marker
2		311	0.36	1.8	
3		392	1.48	5.7	
4		444	0.16	0.5	
5		914	3.46	5.7	
6	▶	1,500	2.10	2.1	Upper Marker

© Copyright 2003 - 2009 Agilent Technologies, Inc.

Assay Class: DNA 1000
Data Path: C:\...-29\2100 expert_DNA 1000_DE13804763_2022-11-29_12-51-34.xad

Created: 11/29/2022 12:51:33 PM
Modified: 11/29/2022 1:34:25 PM

Electropherogram Summary Continued ...

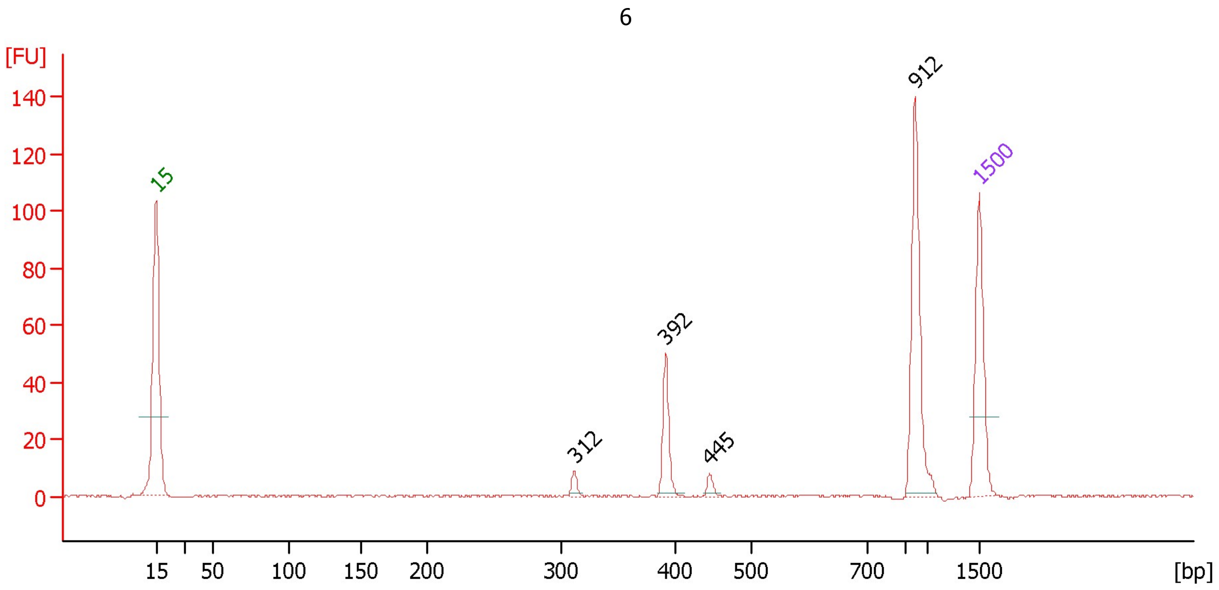

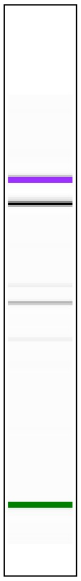

Overall Results for sample 6 : **6**

Number of peaks found: 4

Peak table for sample 6 : **6**

Peak		Size [bp]	Conc. [ng/µl]	Molarity [nmol/l]	Observations
1	◀	15	4.20	424.2	Lower Marker
2		312	0.18	0.9	
3		392	0.99	3.8	
4		445	0.15	0.5	
5		912	3.21	5.3	
6	▶	1,500	2.10	2.1	Upper Marker

 © Copyright 2003 - 2009 Agilent Technologies, Inc.

Assay Class: DNA 1000
Data Path: C:\...-29\2100 expert_DNA 1000_DE13804763_2022-11-29_12-51-34.xad
Created: 11/29/2022 12:51:33 PM
Modified: 11/29/2022 1:34:25 PM

Electropherogram Summary Continued ...

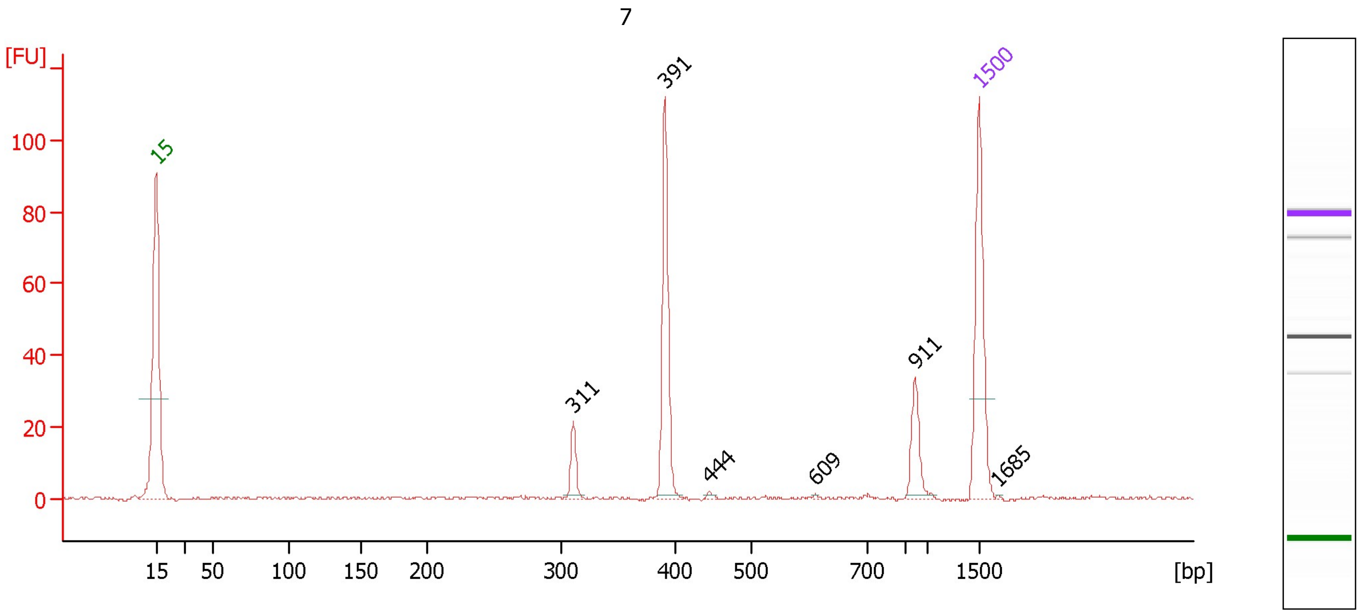

Overall Results for sample 7 : _7_

Number of peaks found: 5

Peak table for sample 7 : _7_

Peak		Size [bp]	Conc. [ng/µl]	Molarity [nmol/l]	Observations
1	◀	15	4.20	424.2	Lower Marker
2		311	0.40	2.0	
3		391	2.09	8.1	
4		444	0.03	0.1	
5		609	0.01	0.0	
6		911	0.71	1.2	
7	▶	1,500	2.10	2.1	Upper Marker
8		1,685	0.00	0.0	

 © Copyright 2003 - 2009 Agilent Technologies, Inc.

Assay Class: DNA 1000
Data Path: C:\...-29\2100 expert_DNA 1000_DE13804763_2022-11-29_12-51-34.xad

Created: 11/29/2022 12:51:33 PM
Modified: 11/29/2022 1:34:25 PM

Electropherogram Summary Continued ...

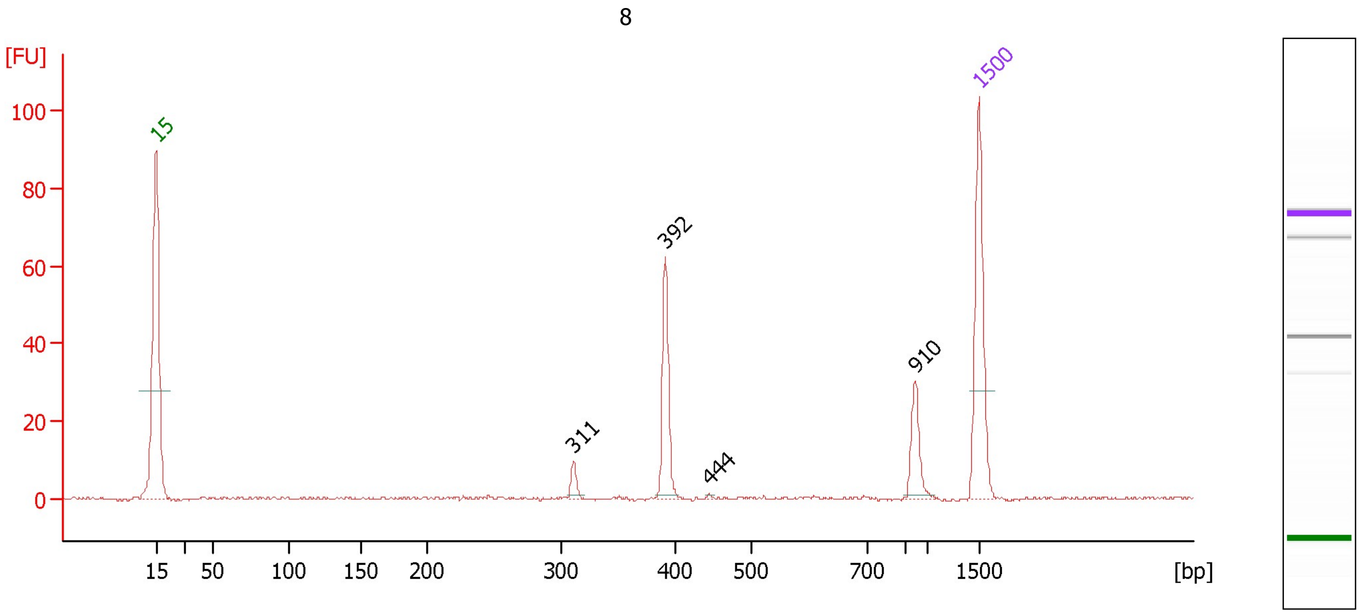

Overall Results for sample 8 : **8**

Number of peaks found: 4

Peak table for sample 8 : **8**

**Peak**		**Size [bp]**	**Conc. [ng/µl]**	**Molarity [nmol/l]**	**Observations**
1	◀	15	4.20	424.2	Lower Marker
2		311	0.20	1.0	
3		392	1.26	4.9	
4		444	0.01	0.0	
5		910	0.70	1.2	
6	▶	1,500	2.10	2.1	Upper Marker

 © Copyright 2003 - 2009 Agilent Technologies, Inc.

Assay Class: DNA 1000
Data Path: C:\...-29\2100 expert_DNA 1000_DE13804763_2022-11-29_12-51-34.xad

Created: 11/29/2022 12:51:33 PM
Modified: 11/29/2022 1:34:25 PM

Electropherogram Summary Continued ...

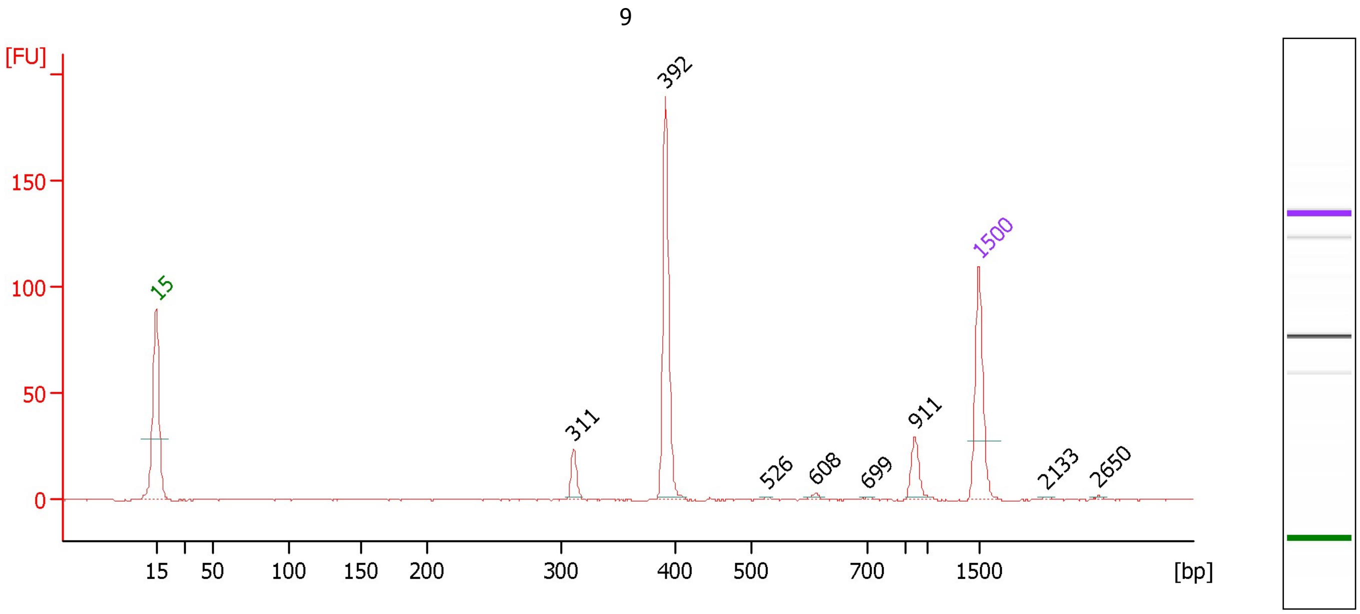

Overall Results for sample 9 : 9

Number of peaks found: 6

Peak table for sample 9 : 9

Peak		Size [bp]	Conc. [ng/µl]	Molarity [nmol/l]	Observations
1	◀	15	4.20	424.2	Lower Marker
2		311	0.47	2.3	
3		392	3.62	14.0	
4		526	0.02	0.1	
5		608	0.07	0.2	
6		699	0.03	0.1	
7		911	0.64	1.1	
8	▶	1,500	2.10	2.1	Upper Marker
9		2,133	0.00	0.0	
10		2,650	0.00	0.0	

 © Copyright 2003 - 2009 Agilent Technologies, Inc.

Assay Class: DNA 1000
Data Path: C:\...-29\2100 expert_DNA 1000_DE13804763_2022-11-29_12-51-34.xad

Created: 11/29/2022 12:51:33 PM
Modified: 11/29/2022 1:34:25 PM

Electropherogram Summary Continued ...

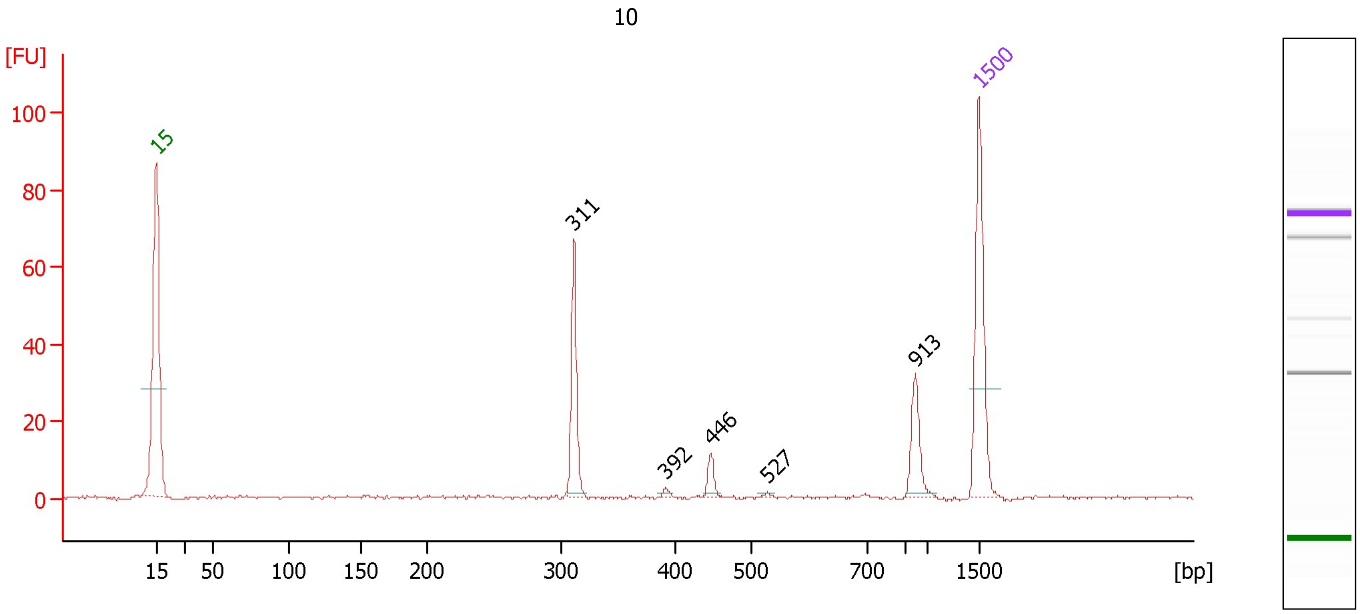

Overall Results for sample 10 : 10

Number of peaks found: 5

Peak table for sample 10 : 10

Peak		Size [bp]	Conc. [ng/µl]	Molarity [nmol/l]	Observations
1	◀	15	4.20	424.2	Lower Marker
2		311	1.38	6.7	
3		392	0.05	0.2	
4		446	0.22	0.7	
5		527	0.02	0.1	
6		913	0.76	1.3	
7	▶	1,500	2.10	2.1	Upper Marker

 © Copyright 2003 - 2009 Agilent Technologies, Inc.

Assay Class: DNA 1000 Created: 11/29/2022 12:51:33 PM
Data Path: C:\...-29\2100 expert_DNA 1000_DE13804763_2022-11-29_12-51-34.xad Modified: 11/29/2022 1:34:25 PM

Electropherogram Summary Continued ...

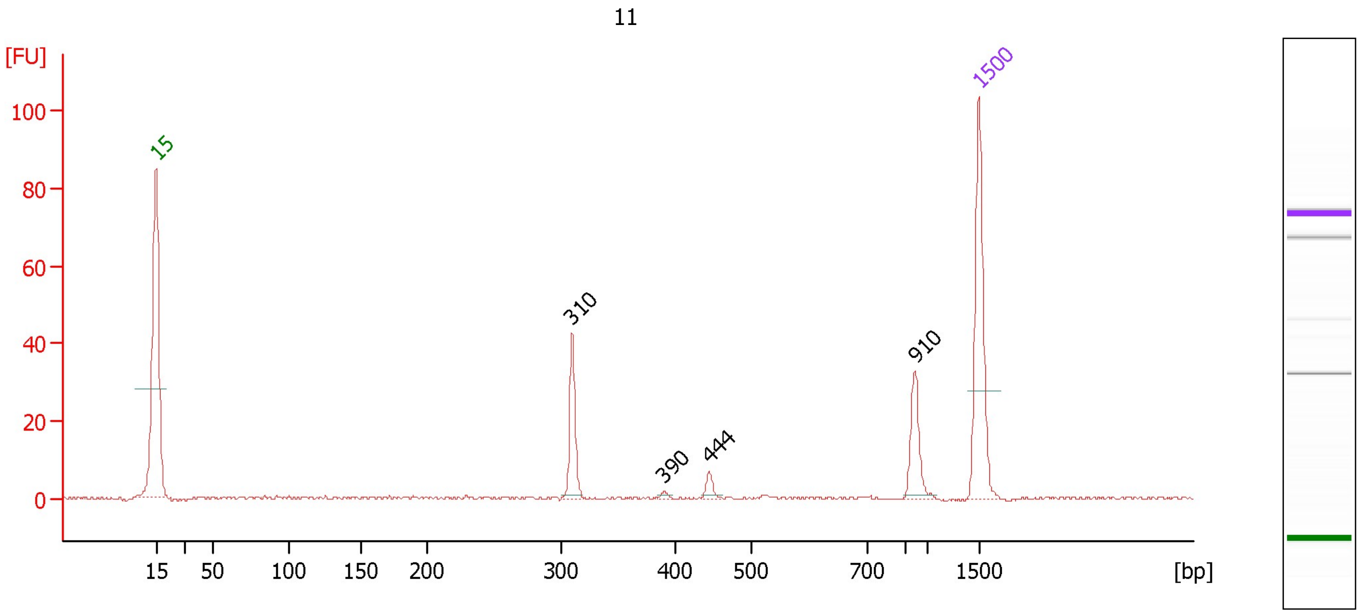

Overall Results for sample 11 : 11

Number of peaks found: 4

Peak table for sample 11 : 11

Peak		Size [bp]	Conc. [ng/µl]	Molarity [nmol/l]	Observations
1	◀	15	4.20	424.2	Lower Marker
2		310	0.89	4.3	
3		390	0.04	0.1	
4		444	0.14	0.5	
5		910	0.80	1.3	
6	▶	1,500	2.10	2.1	Upper Marker

 © Copyright 2003 - 2009 Agilent Technologies, Inc.

Assay Class: DNA 1000 Created: 11/29/2022 12:51:33 PM
Data Path: C:\...-29\2100 expert_DNA 1000_DE13804763_2022-11-29_12-51-34.xad Modified: 11/29/2022 1:34:25 PM

Electropherogram Summary Continued ...

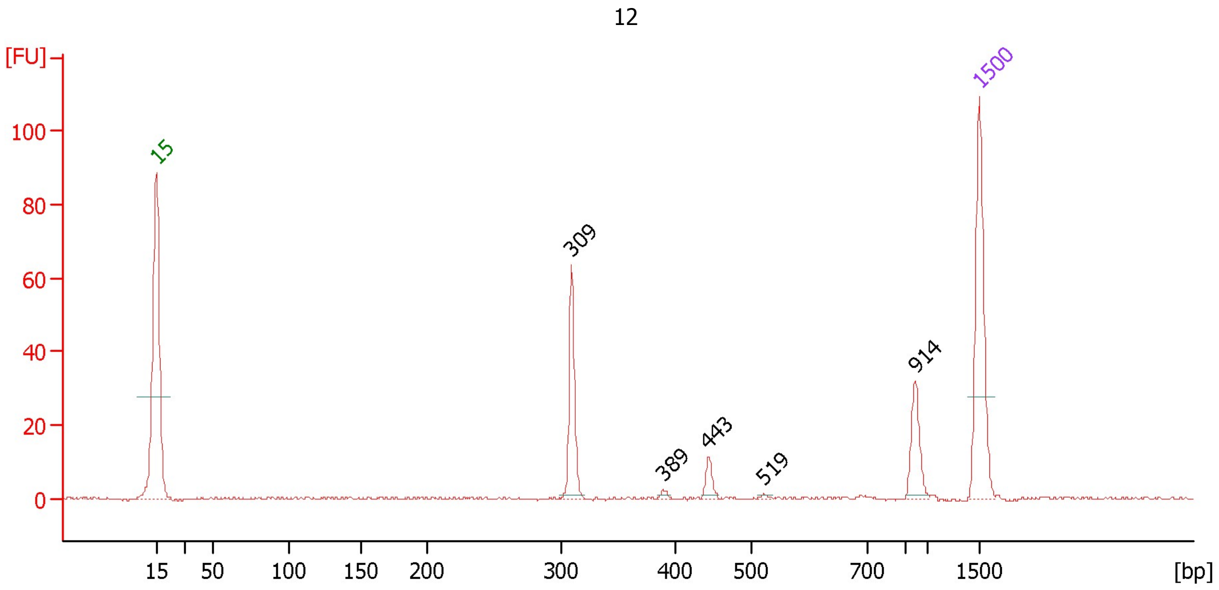

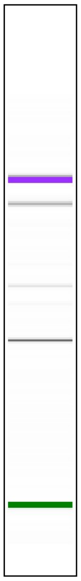

Overall Results for sample 12 : 12

Number of peaks found: 5

Peak table for sample 12 : 12

Peak		Size [bp]	Conc. [ng/µl]	Molarity [nmol/l]	Observations
1	◀	15	4.20	424.2	Lower Marker
2		309	1.23	6.0	
3		389	0.05	0.2	
4		443	0.22	0.7	
5		519	0.03	0.1	
6		914	0.71	1.2	
7	▶	1,500	2.10	2.1	Upper Marker

 © Copyright 2003 - 2009 Agilent Technologies, Inc.

Assay Class: DNA 1000
Data Path: C:\...-29\2100 expert_DNA 1000_DE13804763_2022-11-29_12-51-34.xad

Created: 11/29/2022 12:51:33 PM
Modified: 11/29/2022 1:34:25 PM

Gel Image

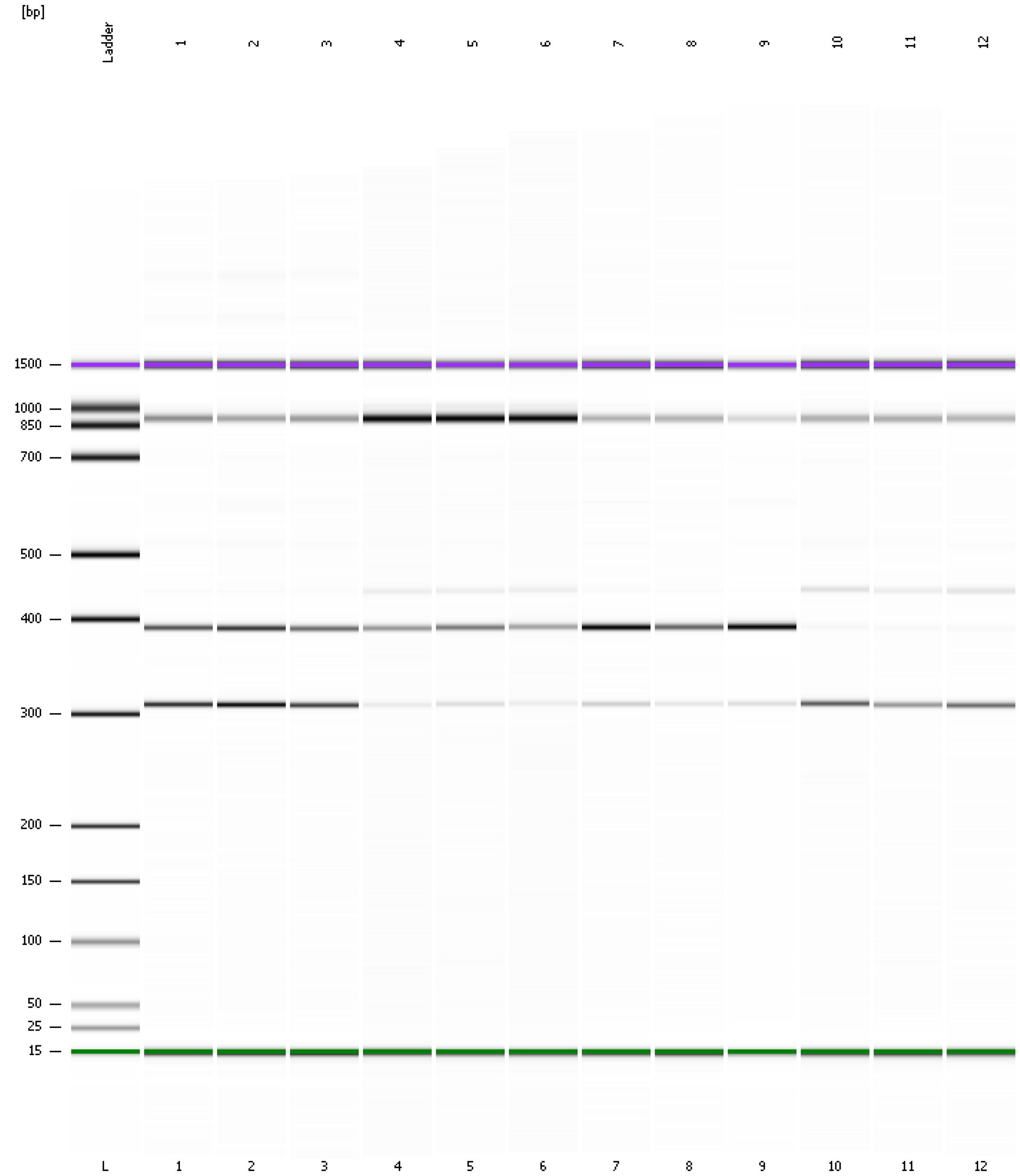

© Copyright 2003 - 2009 Agilent Technologies, Inc.
