# Supplementary material for: Competition for the conserved branch point sequence influences physiological outcomes in pre-mRNA splicing
Source: eLife. 2026 Mar 20;13:RP103167. doi: 10.7554/eLife.103167 (PMC13004596; doi:10.7554/eLife.103167)
Supplement: Figure 3—figure supplement 1—source data 1. [file elife-103167-fig3-figsupp1-data1.pdf]

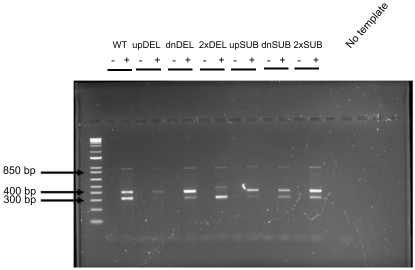

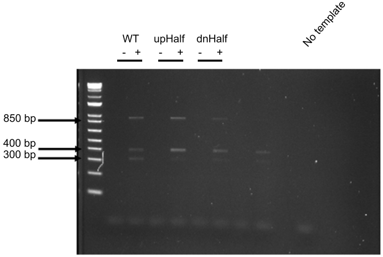

Supplemental Figure S3—source data 1. Original agarose gel images for Supplemental Figure S3. Original uncropped agarose gel images showing RT-PCR amplification of RAI14 reporter constructs in the absen-ce (−) or presence (+) of reverse transcriptase. The top panel displays the original gel for WT, upDEL, dnDEL, 2xDEL, upSUB, dnSUB, and 2xSUB constructs as depicted in the main manuscript. The bottom panel displays the original gel for WT, upHalf, and dnHalf constructs. Following the dnHalf samples on the bottom gel, lanes correspond to a mutant construct with a deletion of the second ACU motif; these lanes were not depicted in the final version of Supplemental Figure S3. The first lane of each gel corresponds to the molecular weight ladder with sizes indicated in base pairs (bp).
