# Supplementary material for: Competition for the conserved branch point sequence influences physiological outcomes in pre-mRNA splicing
Source: eLife. 2026 Mar 20;13:RP103167. doi: 10.7554/eLife.103167 (PMC13004596; doi:10.7554/eLife.103167)
Supplement: Figure 4—source data 1. [file elife-103167-fig4-data1.pdf]

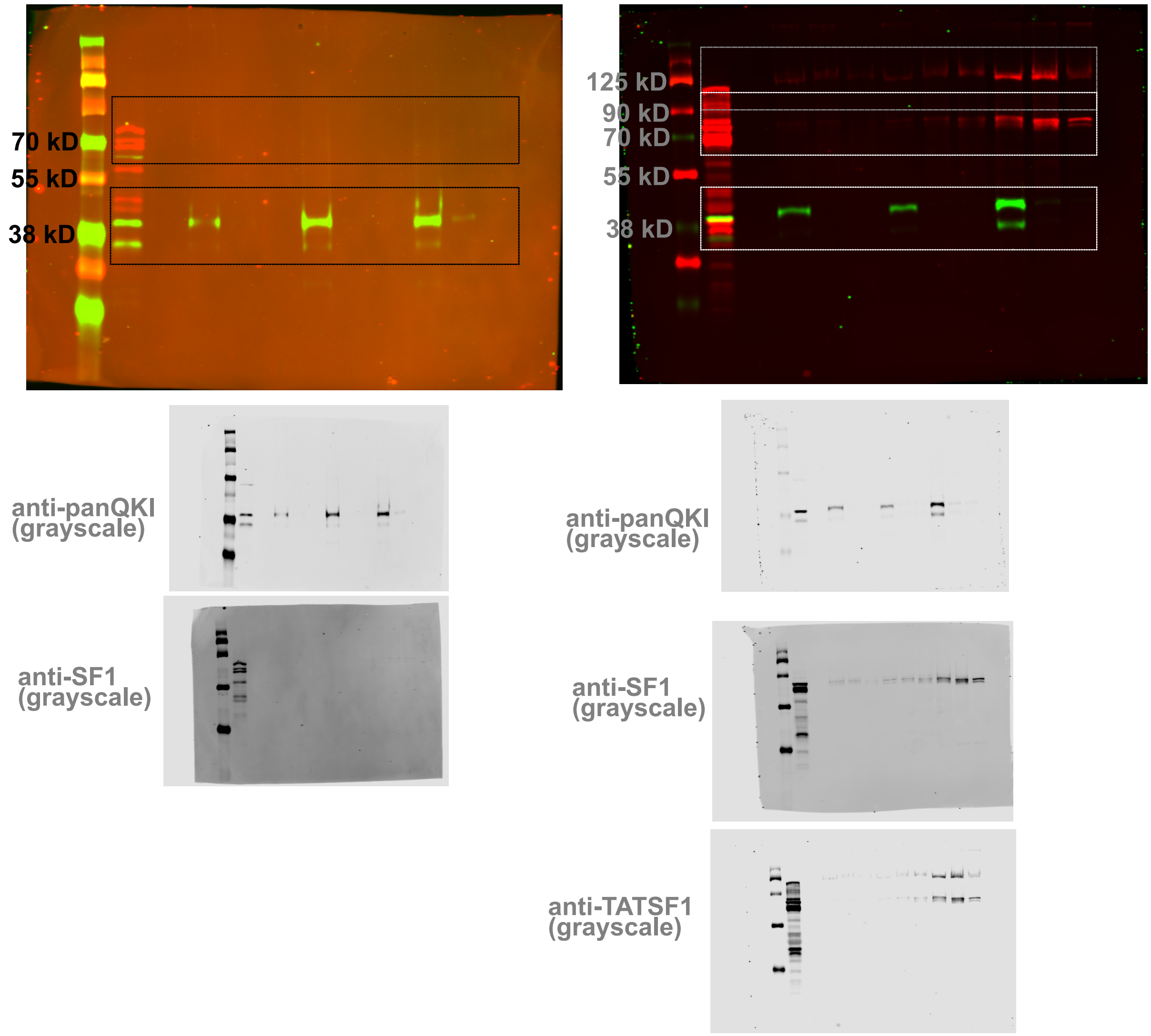

Figure 4, Source Data 1. Original membranes corresponding to western blot depicted in Figure 4 panel D (left): top panel shows color image (QKI in green and SF1 in red), and each individual channel as grayscale (underneath), which were used in Fig4 panel D. Original membrane from Figure 4 panel G: top shows QKI in green, SF1 in red (middle boxed area), and TATSF1 in red (top box). QKI and SF1 were originally probed and imaged with antibodies to each, then TATSF1 was re-probed and imaged the following day. Each individual grayscale image (used in Figure 4 panel G) is shown below.
