# Supplementary material for: Competition for the conserved branch point sequence influences physiological outcomes in pre-mRNA splicing
Source: eLife. 2026 Mar 20;13:RP103167. doi: 10.7554/eLife.103167 (PMC13004596; doi:10.7554/eLife.103167)
Supplement: Figure 5—source data 1. [file elife-103167-fig5-data1.pdf]

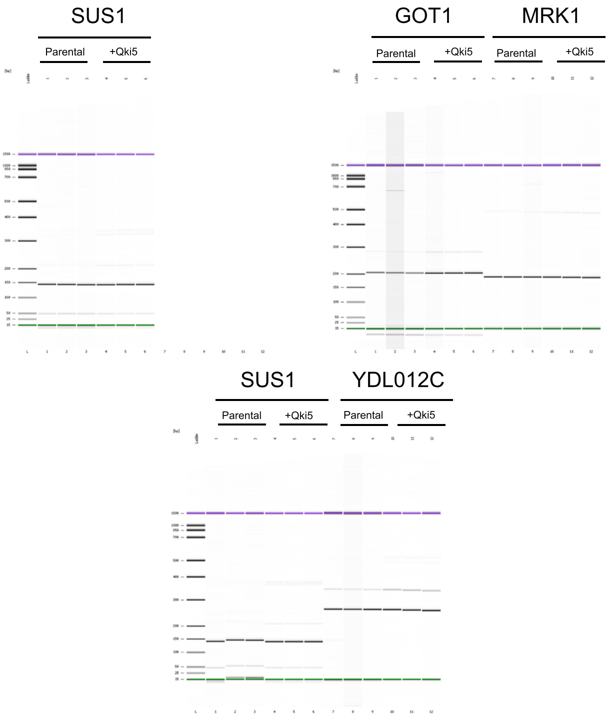

Figure 5—source data 1. Original capillary electrophoresis (BioAnalyzer) images for Figure 5D. Original uncropped BioAnalyzer gel-like images showing the splicing patterns of SUS1, GOT1, MRK1, and YDL012C genes in parental cells and cells expressing +Qki5. The top panel displays the original images for SUS1, GOT1, and MRK1 as depicted in the main manuscript. The bottom panel displays the original image for YDL012C used in the manuscript, alongside an additional BioAnalyzer image for SUS1 that was excluded from the final version of Figure 5D due to insufficient gel quality. Lane L corresponds to the molecular weight ladder with sizes indicated in base pairs (bp).
