# Supplementary material for: Competition for the conserved branch point sequence influences physiological outcomes in pre-mRNA splicing
Source: eLife. 2026 Mar 20;13:RP103167. doi: 10.7554/eLife.103167 (PMC13004596; doi:10.7554/eLife.103167)
Supplement: Figure 5—source data 2. [file elife-103167-fig5-data2.zip › Fig5/GCM2236_3_26_24_yeast4h_sus1(bad)_YDL012C.pdf]

Assay Class: DNA 1000
Data Path: C:\...-26\2100 expert_DNA 1000_DE13804763_2024-03-26_07-52-06.xad

Created: 3/26/2024 7:52:05 AM
Modified: 3/26/2024 8:39:19 AM

Electrophoresis File Run Summary

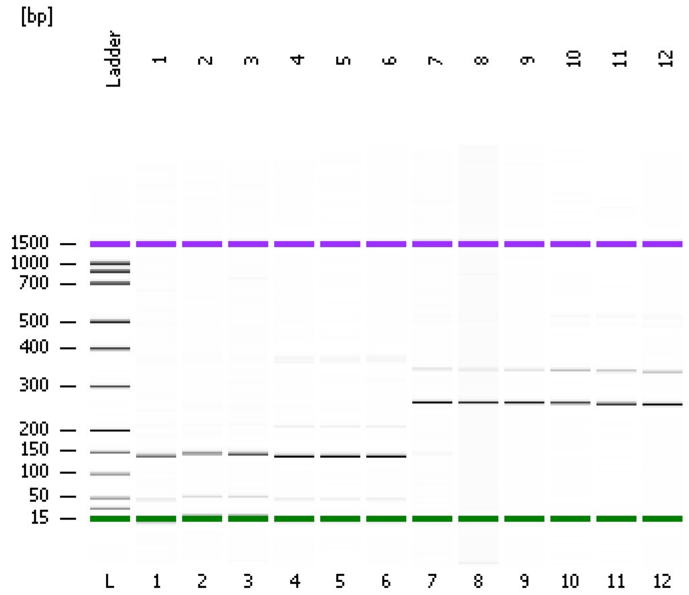

Instrument Information:

Instrument Name:	DE13804763	Firmware:	C.01.069
Serial#:	DE13804763	Type:	G2939A

Assay Information:

Assay Origin Path:	C:\Program Files\Agilent\2100 bioanalyzer\2100 expert\assays\dsDNA\DNA 1000 Series II.xsy
Assay Class:	DNA 1000
Version:	2.3
Assay Comments:	DNA Analysis 25 -1000 bp

© Copyright 2003-2009 Agilent Technologies, Inc.

Chip Information:

Chip Lot #:
Reagent Kit Lot #:
Chip Comments:

1

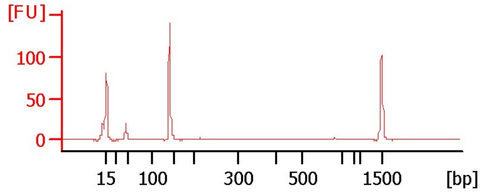

2

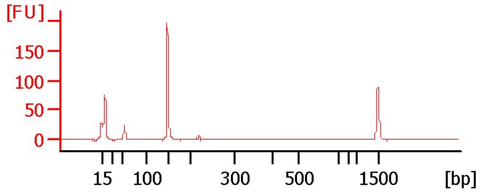

3

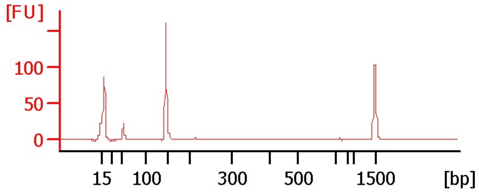

4

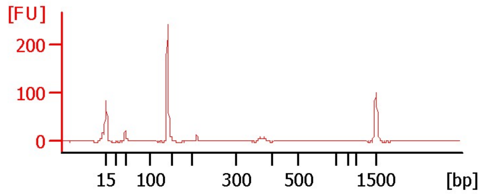

5

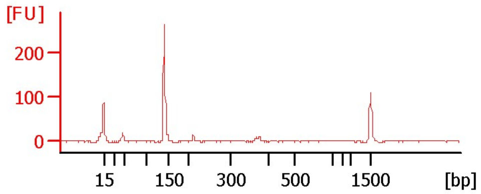

6

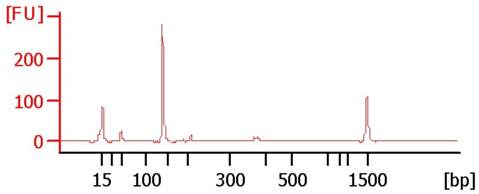

7

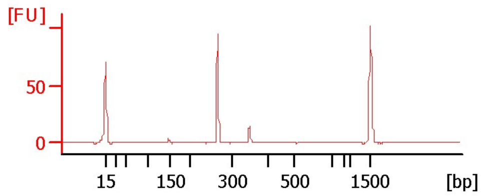

8

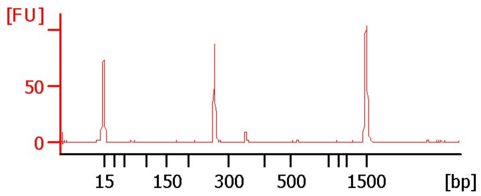

9

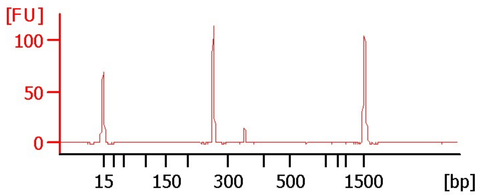

10

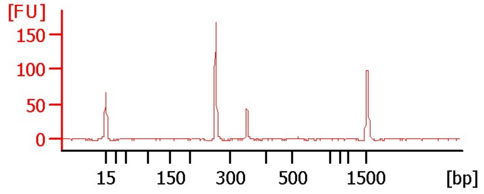

11

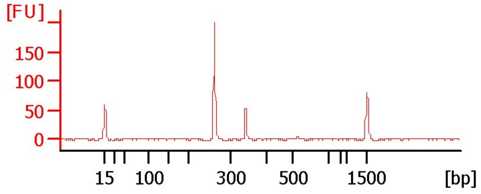

12

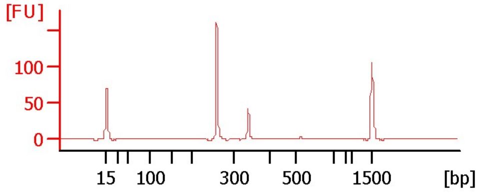

Assay Class: DNA 1000
Data Path: C:\...-26\2100 expert_DNA 1000_DE13804763_2024-03-26_07-52-06.xad

Created: 3/26/2024 7:52:05 AM
Modified: 3/26/2024 8:39:19 AM

Electrophoresis File Run Summary (Chip Summary)

Sample Name	Sample Comment	Rest. Digest	Status	Observation	Result Label	Result Color
1		☐	✓			
2		☐	✓			
3		☐	✓			
4		☐	✓			
5		☐	✓			
6		☐	✓			
7		☐	✓			
8		☐	✓			
9		☐	✓			
10		☐	✓			
11		☐	✓			
12		☐	✓			
Ladder		☐	✓			

Chip Lot #

Reagent Kit Lot #

Chip Comments :

© Copyright 2003 - 2009 Agilent Technologies, Inc.

Assay Class: DNA 1000 Created: 3/26/2024 7:52:05 AM
Data Path: C:\...-26\2100 expert_DNA 1000_DE13804763_2024-03-26_07-52-06.xad Modified: 3/26/2024 8:39:19 AM

Electrophoresis Assay Details

General Analysis Settings

Number of Available Sample and Ladder Wells (Max.) : 13
Minimum Visible Range [s] : 30
Maximum Visible Range [s] : 129
Start Analysis Time Range [s] : 30
End Analysis Time Range [s] : 128.95
Ladder Concentration [ng/µl] : 44
Uses Standard Area for Ladder Fragments
Lower Marker Concentration [ng/µl] : 4.2
Upper Marker Concentration [ng/µl] : 2.1
Used Upper Marker for Quantitation
Standard Curve Fit is Point to Point
Show Data Aligned to Lower and Upper Marker

Integrator Settings

Integration Start Time [s] : 30
Integration End Time [s] : 128.95
Slope Threshold : 0.5
Height Threshold [FU] : 1
Area Threshold : 0.1
Width Threshold [s] : 0.5
Baseline Plateau [s] : 0.5

Filter Settings

Filter Width [s] : 0.5
Polynomial Order : 4

Ladder

Ladder Peak	Size	Area
1	15	25
2	25	26
3	50	34
4	100	41
5	150	45
6	200	52
7	300	63
8	400	76
9	500	83
10	700	88
11	850	86
12	1000	90
13	1500	52

 © Copyright 2003 - 2009 Agilent Technologies, Inc.

Assay Class: DNA 1000
Data Path: C:\...-26\2100 expert_DNA 1000_DE13804763_2024-03-26_07-52-06.xad

Created: 3/26/2024 7:52:05 AM
Modified: 3/26/2024 8:39:19 AM

Electropherogram Summary

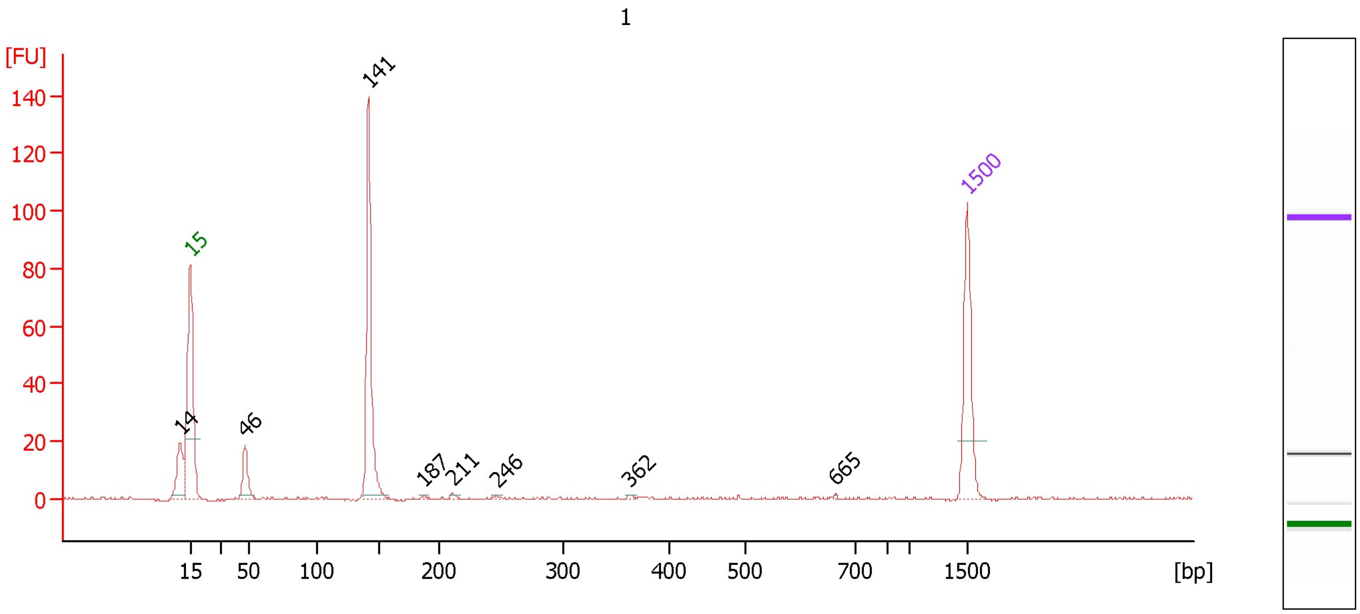

Overall Results for sample 1 : 1

Number of peaks found: 7

Peak table for sample 1 : 1

Peak		Size [bp]	Conc. [ng/µl]	Molarity [nmol/l]	Observations
1		14	0.00	0.0	
2	◀	15	4.20	424.2	Lower Marker
3		46	0.78	25.7	
4		141	4.04	43.4	
5		187	0.02	0.2	
6		211	0.05	0.3	
7		246	0.03	0.2	
8		362	0.03	0.1	
9		665	0.01	0.0	
10	▶	1,500	2.10	2.1	Upper Marker

© Copyright 2003 - 2009 Agilent Technologies, Inc.

Assay Class: DNA 1000
Data Path: C:\...-26\2100 expert_DNA 1000_DE13804763_2024-03-26_07-52-06.xad
Created: 3/26/2024 7:52:05 AM
Modified: 3/26/2024 8:39:19 AM

Electropherogram Summary Continued ...

2

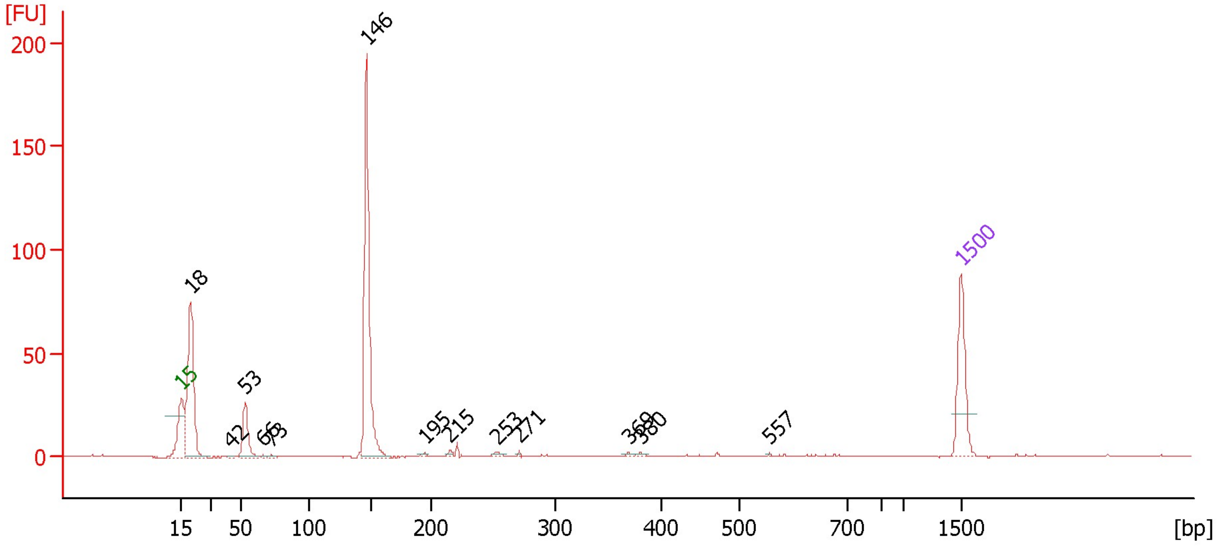

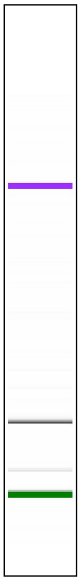

Overall Results for sample 2 : 2

Number of peaks found: 13

Peak table for sample 2 : 2

Peak		Size [bp]	Conc. [ng/µl]	Molarity [nmol/l]	Observations
1	◀	15	4.20	424.2	Lower Marker
2		18	6.22	521.8	
3		42	0.04	1.4	
4		53	1.33	37.9	
5		66	0.04	0.8	
6		73	0.07	1.4	
7		146	6.68	69.3	
8		195	0.07	0.6	
9		215	0.09	0.6	
10		253	0.09	0.6	
11		271	0.03	0.2	
12		369	0.04	0.2	
13		380	0.04	0.2	
14		557	0.01	0.0	
15	▶	1,500	2.10	2.1	Upper Marker

 © Copyright 2003 - 2009 Agilent Technologies, Inc.

Assay Class: DNA 1000 Created: 3/26/2024 7:52:05 AM
Data Path: C:\...-26\2100 expert_DNA 1000_DE13804763_2024-03-26_07-52-06.xad Modified: 3/26/2024 8:39:19 AM

Electropherogram Summary Continued ...

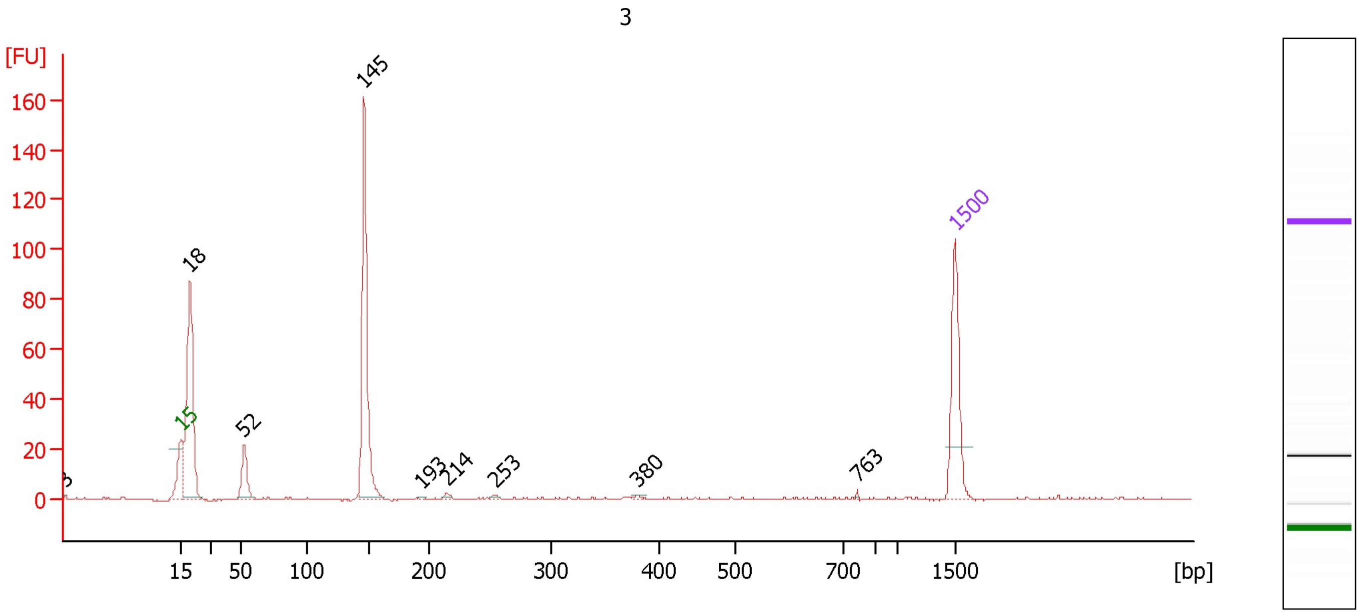

Overall Results for sample 3 : 3

Number of peaks found: 8

Peak table for sample 3 : 3

Peak		Size [bp]	Conc. [ng/µl]	Molarity [nmol/l]	Observations
1		3	0.00	0.0	
2	◀	15	4.20	424.2	Lower Marker
3		18	5.99	507.6	
4		52	0.89	25.6	
5		145	4.70	49.0	
6		193	0.04	0.3	
7		214	0.08	0.5	
8		253	0.05	0.3	
9		380	0.03	0.1	
10		763	0.02	0.0	
11	▶	1,500	2.10	2.1	Upper Marker

 © Copyright 2003 - 2009 Agilent Technologies, Inc.

Assay Class: DNA 1000
Data Path: C:\...-26\2100 expert_DNA 1000_DE13804763_2024-03-26_07-52-06.xad
Created: 3/26/2024 7:52:05 AM
Modified: 3/26/2024 8:39:19 AM

Electropherogram Summary Continued ...

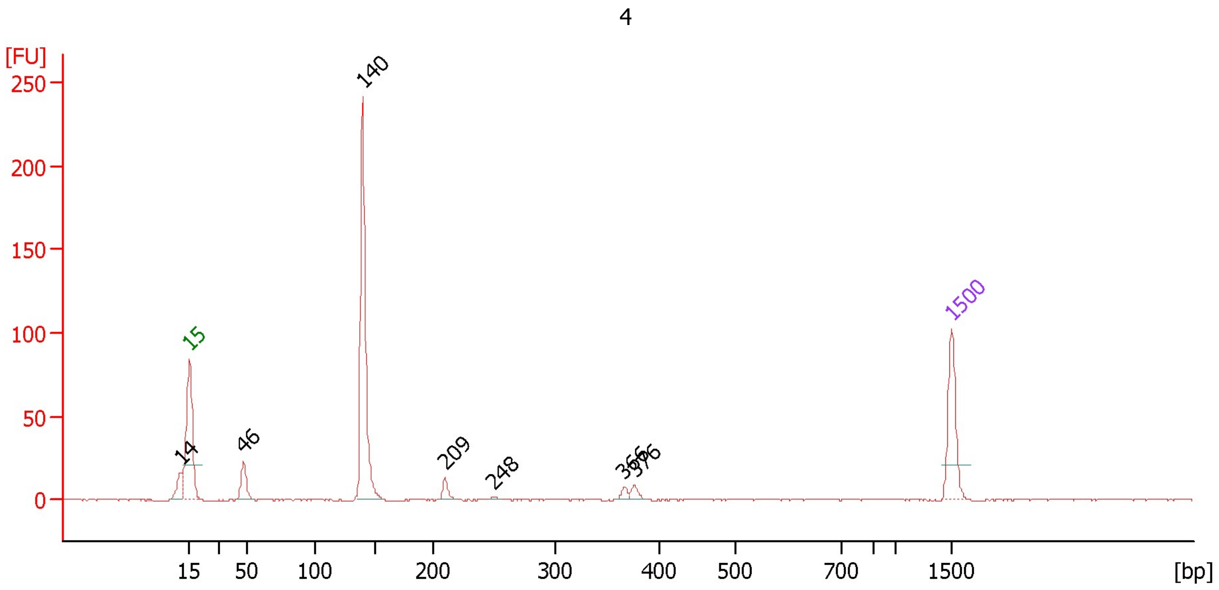

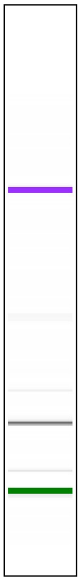

Overall Results for sample 4 : **4**

Number of peaks found: 6

Peak table for sample 4 : **4**

Peak		Size [bp]	Conc. [ng/µl]	Molarity [nmol/l]	Observations
1		14	0.00	0.0	
2	◀	15	4.20	424.2	Lower Marker
3		46	0.98	32.1	
4		140	7.20	77.9	
5		209	0.34	2.4	
6		248	0.04	0.3	
7		366	0.19	0.8	
8		376	0.22	0.9	
9	▶	1,500	2.10	2.1	Upper Marker

 © Copyright 2003 - 2009 Agilent Technologies, Inc.

Assay Class: DNA 1000
Data Path: C:\...-26\2100 expert_DNA 1000_DE13804763_2024-03-26_07-52-06.xad

Created: 3/26/2024 7:52:05 AM
Modified: 3/26/2024 8:39:19 AM

Electropherogram Summary Continued ...

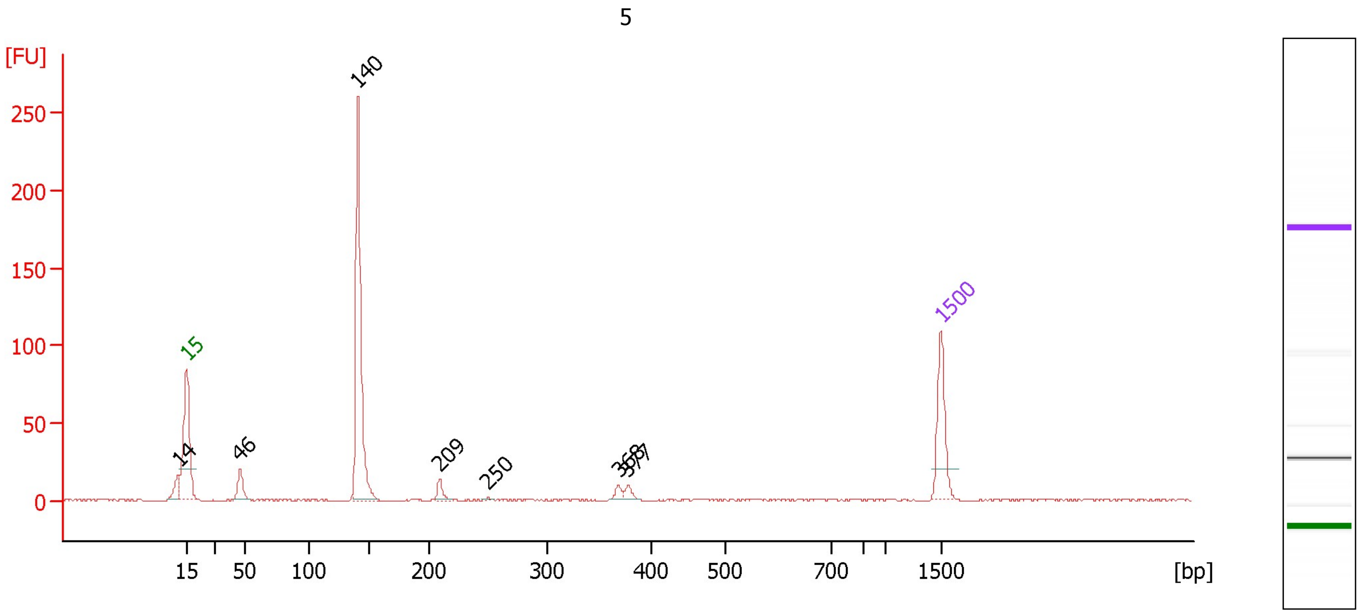

Overall Results for sample 5 : 5

Number of peaks found: 6

Peak table for sample 5 : 5

Peak		Size [bp]	Conc. [ng/µl]	Molarity [nmol/l]	Observations
1		14	0.00	0.0	
2	◀	15	4.20	424.2	Lower Marker
3		46	0.81	26.5	
4		140	7.56	82.1	
5		209	0.35	2.5	
6		250	0.04	0.3	
7		368	0.22	0.9	
8		377	0.24	1.0	
9	▶	1,500	2.10	2.1	Upper Marker

 © Copyright 2003 - 2009 Agilent Technologies, Inc.

Assay Class: DNA 1000
Data Path: C:\...-26\2100 expert_DNA 1000_DE13804763_2024-03-26_07-52-06.xad

Created: 3/26/2024 7:52:05 AM
Modified: 3/26/2024 8:39:19 AM

Electropherogram Summary Continued ...

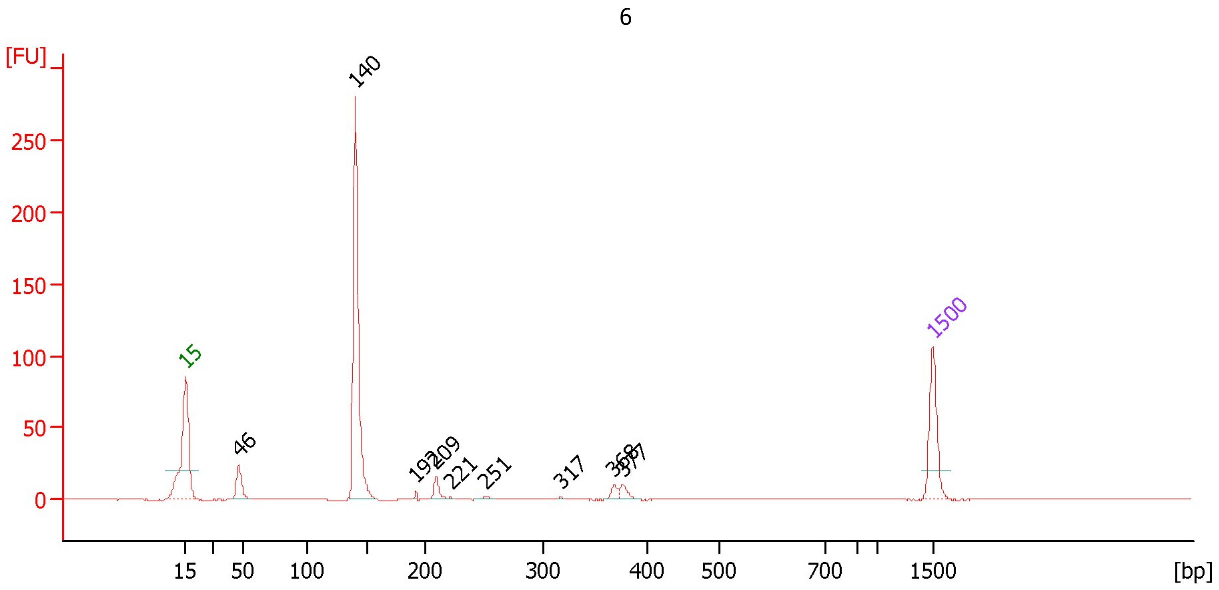

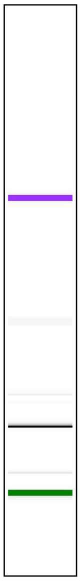

Overall Results for sample 6 : 6

Number of peaks found: 9

Peak table for sample 6 : 6

Peak		Size [bp]	Conc. [ng/µl]	Molarity [nmol/l]	Observations
1	◀	15	4.20	424.2	Lower Marker
2		46	1.00	32.6	
3		140	8.35	90.4	
4		192	0.06	0.5	
5		209	0.40	2.9	
6		221	0.02	0.1	
7		251	0.06	0.4	
8		317	0.02	0.1	
9		368	0.24	1.0	
10		377	0.28	1.1	
11	▶	1,500	2.10	2.1	Upper Marker

 © Copyright 2003 - 2009 Agilent Technologies, Inc.

Assay Class: DNA 1000
Data Path: C:\...-26\2100 expert_DNA 1000_DE13804763_2024-03-26_07-52-06.xad

Created: 3/26/2024 7:52:05 AM
Modified: 3/26/2024 8:39:19 AM

Electropherogram Summary Continued ...

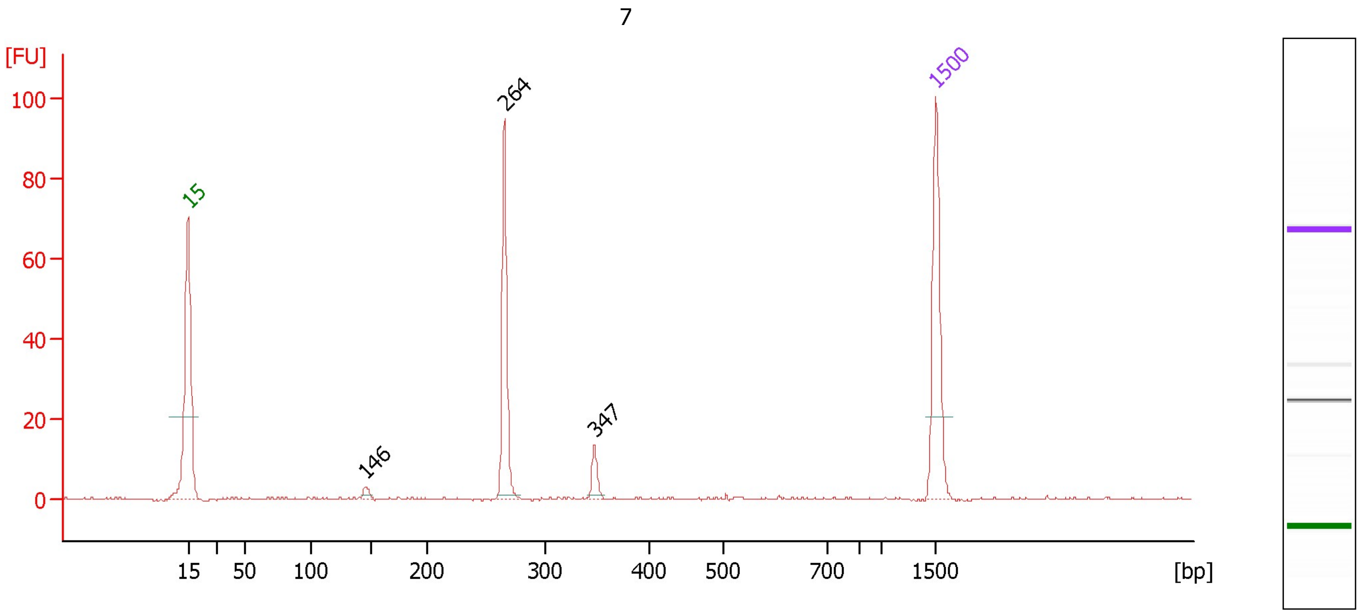

Overall Results for sample 7 : _7_

Number of peaks found: 3

Peak table for sample 7 : _7_

Peak		Size [bp]	Conc. [ng/µl]	Molarity [nmol/l]	Observations
1	◀	15	4.20	424.2	Lower Marker
2		146	0.11	1.2	
3		264	2.13	12.2	
4		347	0.29	1.3	
5	▶	1,500	2.10	2.1	Upper Marker

 © Copyright 2003 - 2009 Agilent Technologies, Inc.

Assay Class: DNA 1000
Data Path: C:\...-26\2100 expert_DNA 1000_DE13804763_2024-03-26_07-52-06.xad

Created: 3/26/2024 7:52:05 AM
Modified: 3/26/2024 8:39:19 AM

Electropherogram Summary Continued ...

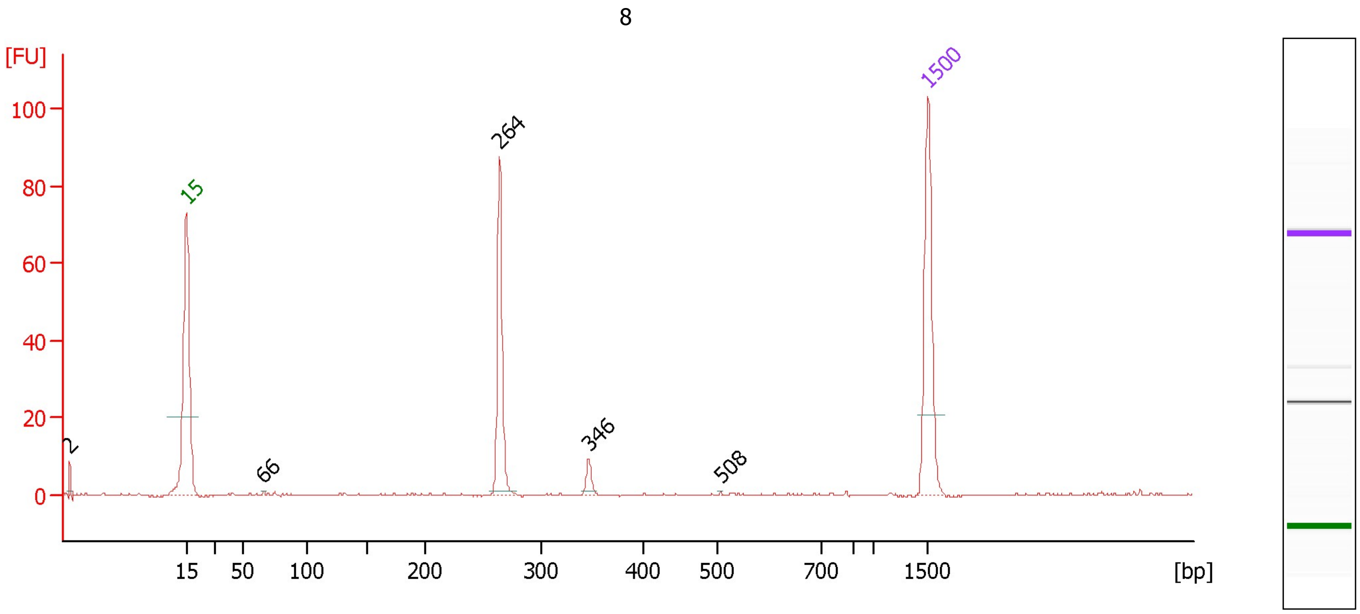

Overall Results for sample 8 : 8

Number of peaks found: 4

Peak table for sample 8 : 8

Peak		Size [bp]	Conc. [ng/µl]	Molarity [nmol/l]	Observations
1		2	0.00	0.0	
2	◀	15	4.20	424.2	Lower Marker
3		66	0.02	0.5	
4		264	1.93	11.1	
5		346	0.19	0.8	
6		508	0.01	0.0	
7	▶	1,500	2.10	2.1	Upper Marker

 © Copyright 2003 - 2009 Agilent Technologies, Inc.

Assay Class: DNA 1000 Created: 3/26/2024 7:52:05 AM
Data Path: C:\...-26\2100 expert_DNA 1000_DE13804763_2024-03-26_07-52-06.xad Modified: 3/26/2024 8:39:19 AM

Electropherogram Summary Continued ...

9

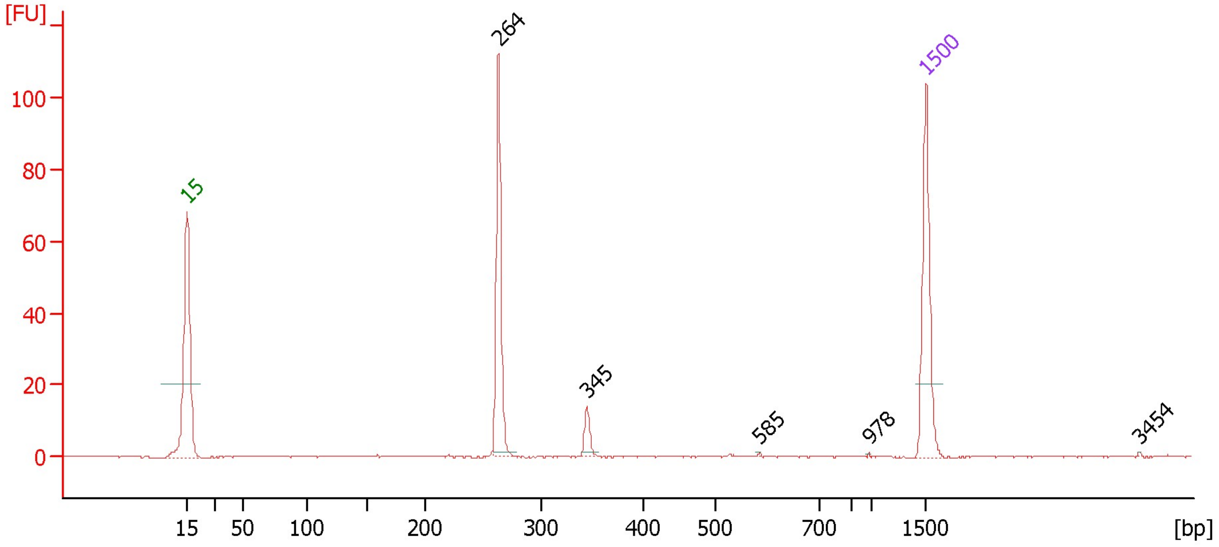

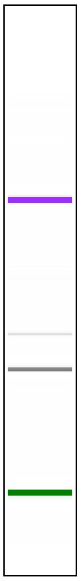

Overall Results for sample 9 : 9

Number of peaks found: 4

Peak table for sample 9 : 9

Peak		Size [bp]	Conc. [ng/µl]	Molarity [nmol/l]	Observations
1	◀	15	4.20	424.2	Lower Marker
2		264	2.56	14.7	
3		345	0.31	1.3	
4		585	0.01	0.0	
5		978	0.01	0.0	
6	▶	1,500	2.10	2.1	Upper Marker
7		3,454	0.00	0.0	

 © Copyright 2003 - 2009 Agilent Technologies, Inc.

Assay Class: DNA 1000
Data Path: C:\...-26\2100 expert_DNA 1000_DE13804763_2024-03-26_07-52-06.xad
Created: 3/26/2024 7:52:05 AM
Modified: 3/26/2024 8:39:19 AM

Electropherogram Summary Continued ...

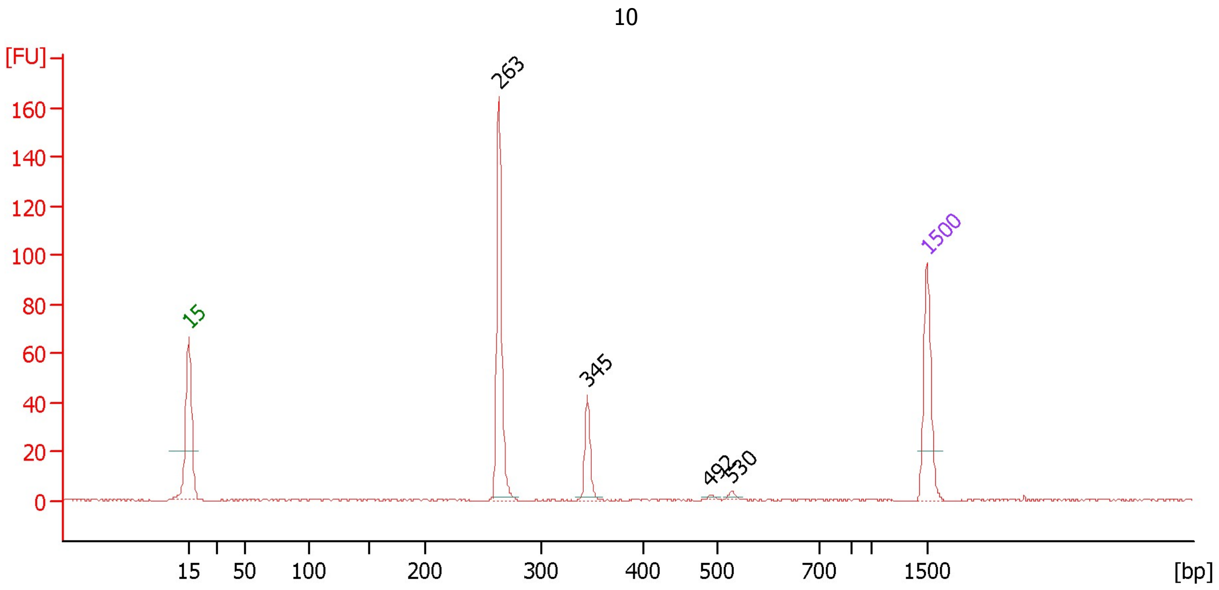

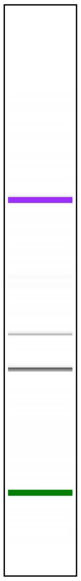

Overall Results for sample 10 : 10

Number of peaks found: 4

Peak table for sample 10 : 10

Peak		Size [bp]	Conc. [ng/µl]	Molarity [nmol/l]	Observations
1	◀	15	4.20	424.2	Lower Marker
2		263	4.05	23.3	
3		345	0.99	4.3	
4		492	0.05	0.2	
5		530	0.09	0.2	
6	▶	1,500	2.10	2.1	Upper Marker

© Copyright 2003 - 2009 Agilent Technologies, Inc.

Assay Class: DNA 1000
Data Path: C:\...-26\2100 expert_DNA 1000_DE13804763_2024-03-26_07-52-06.xad

Created: 3/26/2024 7:52:05 AM
Modified: 3/26/2024 8:39:19 AM

Electropherogram Summary Continued ...

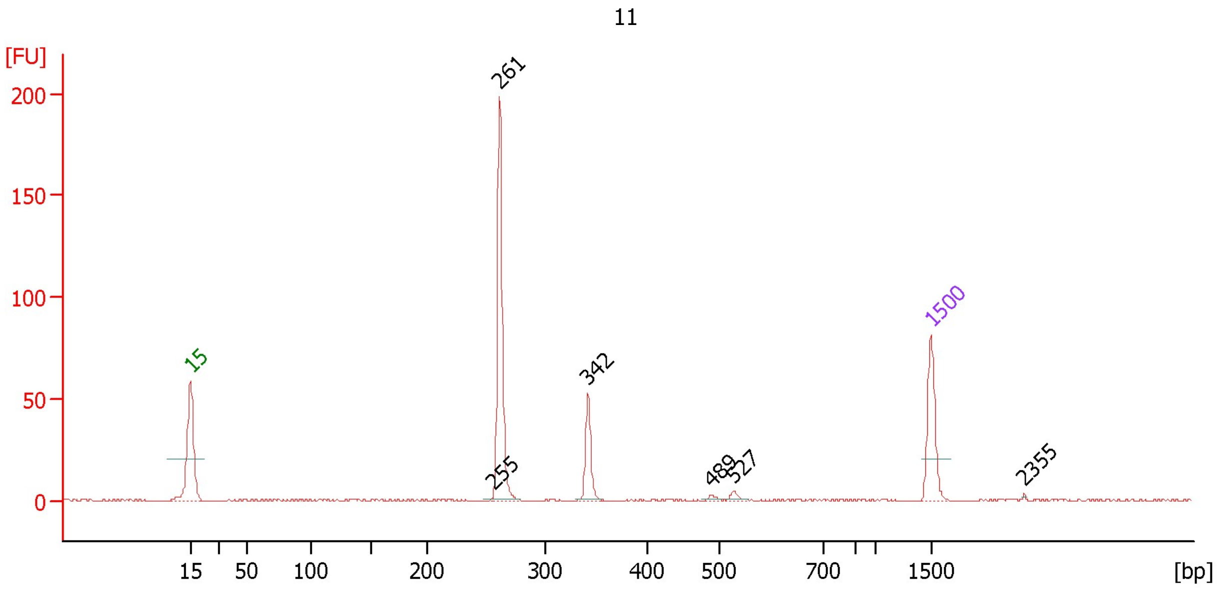

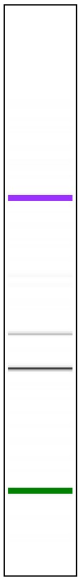

Overall Results for sample 11 : 11

Number of peaks found: 5

Peak table for sample 11 : 11

Peak		Size [bp]	Conc. [ng/µl]	Molarity [nmol/l]	Observations
1	◀	15	4.20	424.2	Lower Marker
2		255	0.03	0.2	
3		261	5.67	32.9	
4		342	1.41	6.2	
5		489	0.08	0.2	
6		527	0.14	0.4	
7	▶	1,500	2.10	2.1	Upper Marker
8		2,355	0.00	0.0	

 © Copyright 2003 - 2009 Agilent Technologies, Inc.

Assay Class: DNA 1000 Created: 3/26/2024 7:52:05 AM
Data Path: C:\...-26\2100 expert_DNA 1000_DE13804763_2024-03-26_07-52-06.xad Modified: 3/26/2024 8:39:19 AM

Electropherogram Summary Continued ...

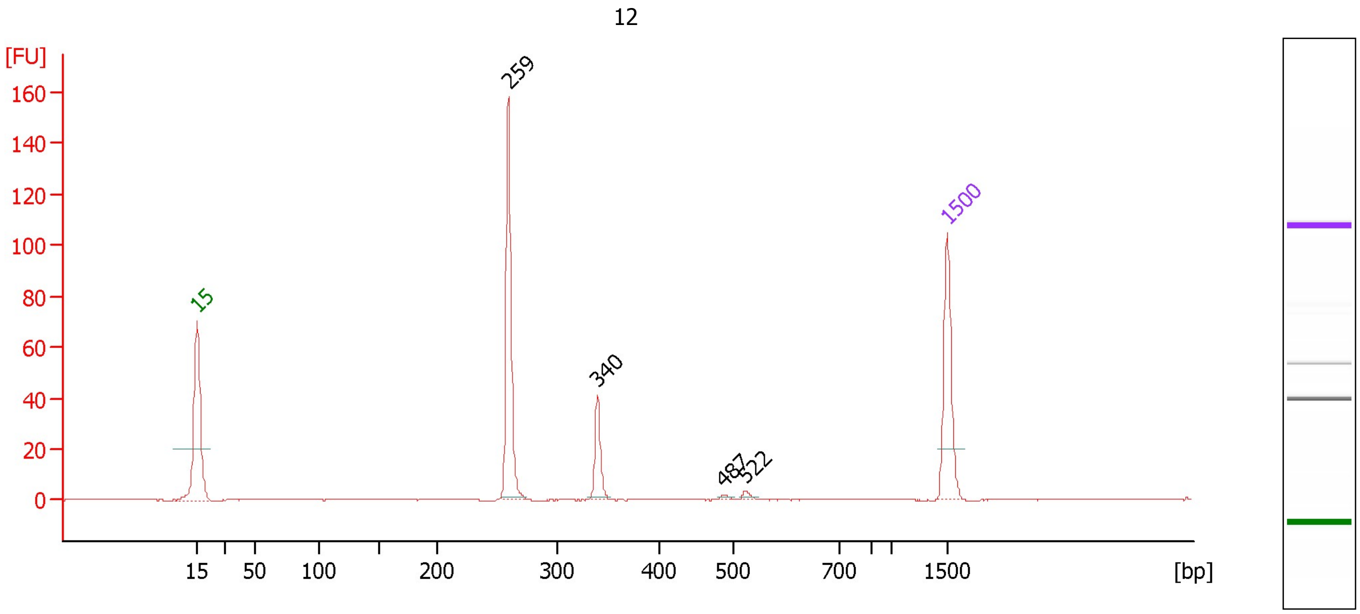

Overall Results for sample 12 : 12

Number of peaks found: 4

Peak table for sample 12 : 12

Peak		Size [bp]	Conc. [ng/µl]	Molarity [nmol/l]	Observations
1	◀	15	4.20	424.2	Lower Marker
2		259	3.60	21.0	
3		340	0.87	3.9	
4		487	0.05	0.1	
5		522	0.08	0.2	
6	▶	1,500	2.10	2.1	Upper Marker

 © Copyright 2003 - 2009 Agilent Technologies, Inc.

Assay Class: DNA 1000
Data Path: C:\...-26\2100 expert_DNA 1000_DE13804763_2024-03-26_07-52-06.xad
Created: 3/26/2024 7:52:05 AM
Modified: 3/26/2024 8:39:19 AM

Gel Image

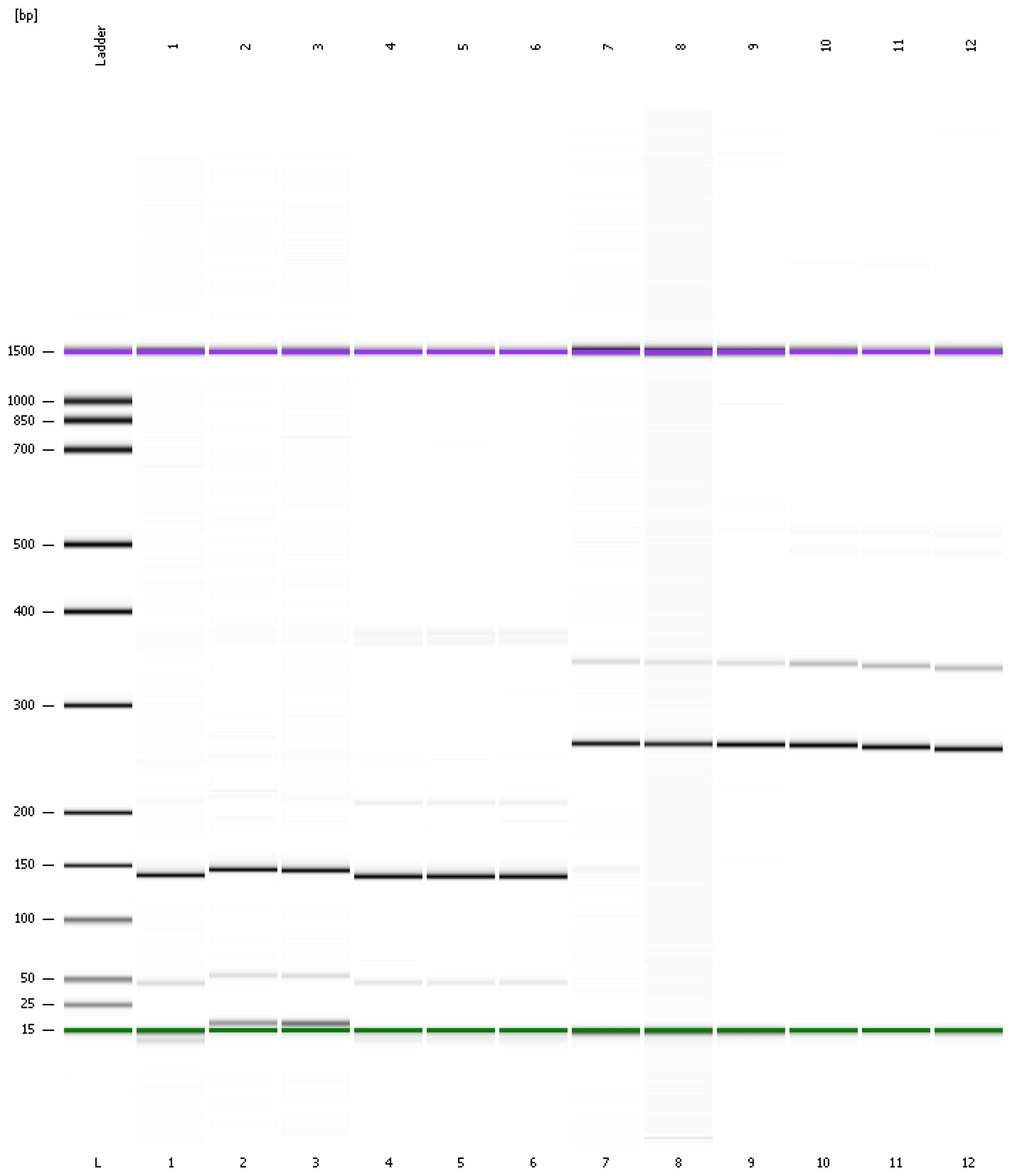

© Copyright 2003 - 2009 Agilent Technologies, Inc.
