# Supplementary material for: Competition for the conserved branch point sequence influences physiological outcomes in pre-mRNA splicing
Source: eLife. 2026 Mar 20;13:RP103167. doi: 10.7554/eLife.103167 (PMC13004596; doi:10.7554/eLife.103167)
Supplement: Figure 5—source data 2. [file elife-103167-fig5-data2.zip › Fig5/2100 expert_DNA 1000_DE13804763_2024-03-28_13-17-54_3_28_24_SUS1.pdf]

Assay Class: DNA 1000
Data Path: C:\...-28\2100 expert_DNA 1000_DE13804763_2024-03-28_13-17-54.xad
Created: 3/28/2024 1:17:53 PM
Modified: 3/28/2024 1:51:17 PM

Electrophoresis File Run Summary

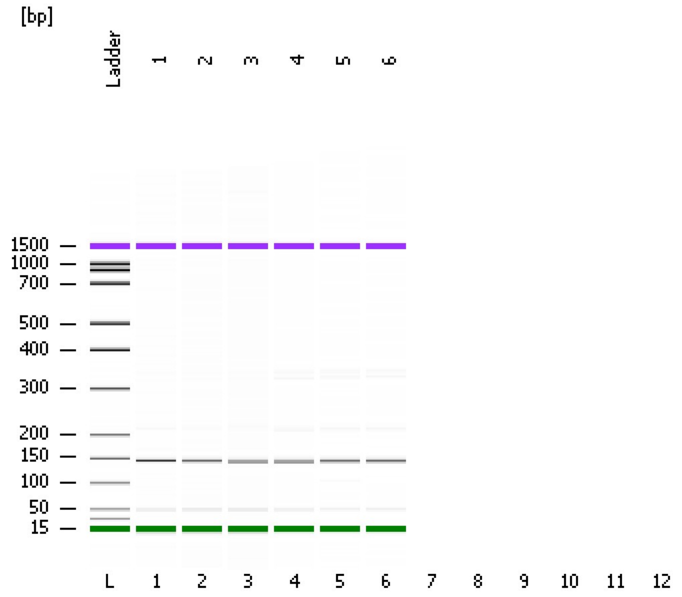

Instrument Information:

Instrument Name: DE13804763 Firmware: C.01.069
Serial#: DE13804763 Type: G2939A

Assay Information:

Assay Origin Path: C:\Program Files\Agilent\2100 bioanalyzer\2100 expert\assays\dsDNA\DNA 1000 Series II.xsy

Assay Class: DNA 1000

Version: 2.3

Assay Comments: DNA Analysis 25 -1000 bp

© Copyright 2003-2009 Agilent Technologies, Inc.

Chip Information:

Chip Lot #:

Reagent Kit Lot #:

Chip Comments:

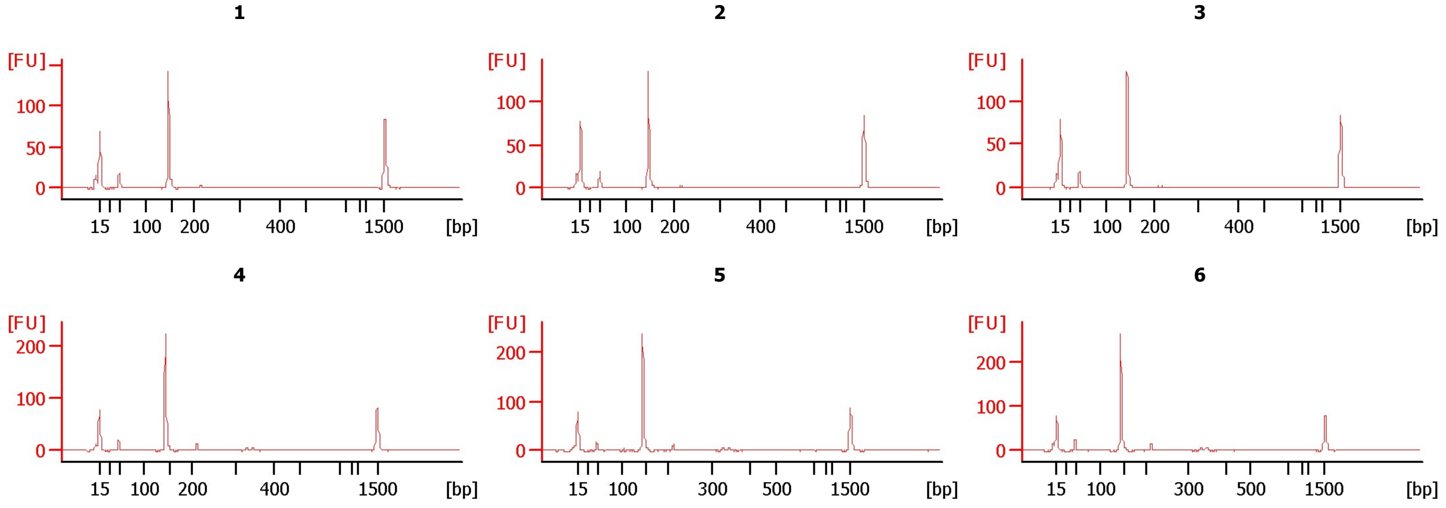

Assay Class: DNA 1000
Data Path: C:\...-28\2100 expert_DNA 1000_DE13804763_2024-03-28_13-17-54.xad
Created: 3/28/2024 1:17:53 PM
Modified: 3/28/2024 1:51:17 PM

Electrophoresis File Run Summary (Chip Summary)

Sample Name	Sample Comment	Rest. Digest	Status	Observation	Result Label	Result Color
1		☐	✔			
2		☐	✔			
3		☐	✔			
4		☐	✔			
5		☐	✔			
6		☐	✔			
		☐				
		☐				
		☐				
		☐				
		☐				
		☐				
Ladder		☐	✔			

Chip Lot # **Reagent Kit Lot #**

Chip Comments :

© Copyright 2003 - 2009 Agilent Technologies, Inc.

Assay Class:	DNA 1000	Created:	3/28/2024 1:17:53 PM
Data Path:	C:\...-28\2100 expert_DNA 1000_DE13804763_2024-03-28_13-17-54.xad	Modified:	3/28/2024 1:51:17 PM

Electrophoresis Assay Details

General Analysis Settings

Number of Available Sample and Ladder Wells (Max.) : 13
Minimum Visible Range [s] : 30
Maximum Visible Range [s] : 129
Start Analysis Time Range [s] : 30
End Analysis Time Range [s] : 128.95
Ladder Concentration [ng/µl] : 44
Uses Standard Area for Ladder Fragments
Lower Marker Concentration [ng/µl] : 4.2
Upper Marker Concentration [ng/µl] : 2.1
Used Upper Marker for Quantitation
Standard Curve Fit is Point to Point
Show Data Aligned to Lower and Upper Marker

Integrator Settings

Integration Start Time [s] : 30
Integration End Time [s] : 128.95
Slope Threshold : 0.5
Height Threshold [FU] : 0.5
Area Threshold : 0.1
Width Threshold [s] : 0.5
Baseline Plateau [s] : 0.5

Filter Settings

Filter Width [s] : 0.5
Polynomial Order : 4

Ladder

Ladder Peak	Size	Area
1	15	25
2	25	26
3	50	34
4	100	41
5	150	45
6	200	52
7	300	63
8	400	76
9	500	83
10	700	88
11	850	86
12	1000	90
13	1500	52

 © Copyright 2003 - 2009 Agilent Technologies, Inc.

Assay Class: DNA 1000
Data Path: C:\...-28\2100 expert_DNA 1000_DE13804763_2024-03-28_13-17-54.xad
Created: 3/28/2024 1:17:53 PM
Modified: 3/28/2024 1:51:17 PM

Electropherogram Summary

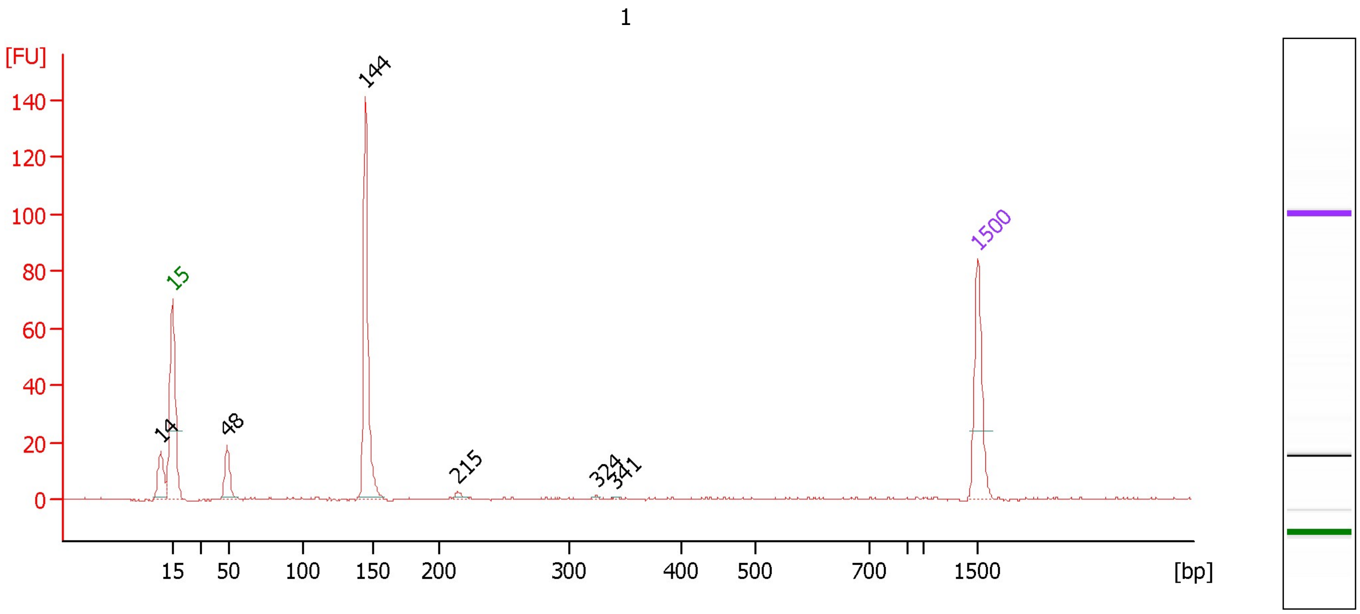

Overall Results for sample 1 : 1

Number of peaks found: 5

Peak table for sample 1 : 1

Peak		Size [bp]	Conc. [ng/µl]	Molarity [nmol/l]	Observations
1		14	0.00	0.0	
2	◀	15	4.20	424.2	Lower Marker
3		48	0.91	28.7	
4		144	4.80	50.5	
5		215	0.10	0.7	
6		324	0.02	0.1	
7		341	0.01	0.1	
8	▶	1,500	2.10	2.1	Upper Marker

© Copyright 2003 - 2009 Agilent Technologies, Inc.

Assay Class: DNA 1000
Data Path: C:\...-28\2100 expert_DNA 1000_DE13804763_2024-03-28_13-17-54.xad
Created: 3/28/2024 1:17:53 PM
Modified: 3/28/2024 1:51:17 PM

Electropherogram Summary Continued ...

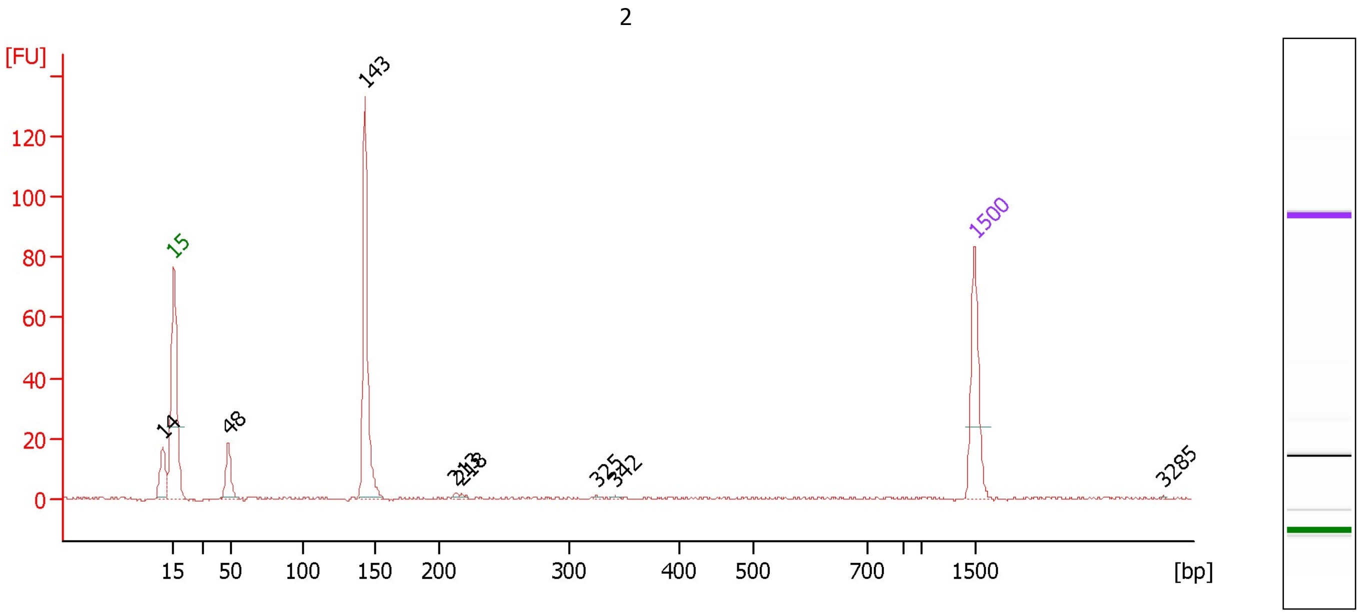

Overall Results for sample 2 : **2**

Number of peaks found: 6

Peak table for sample 2 : **2**

Peak		Size [bp]	Conc. [ng/µl]	Molarity [nmol/l]	Observations
1		14	0.00	0.0	
2	◀	15	4.20	424.2	Lower Marker
3		48	0.89	28.1	
4		143	4.53	47.9	
5		213	0.05	0.3	
6		218	0.04	0.3	
7		325	0.02	0.1	
8		342	0.02	0.1	
9	▶	1,500	2.10	2.1	Upper Marker
10		3,285	0.00	0.0	

© Copyright 2003 - 2009 Agilent Technologies, Inc.

Assay Class: DNA 1000
Data Path: C:\...-28\2100 expert_DNA 1000_DE13804763_2024-03-28_13-17-54.xad
Created: 3/28/2024 1:17:53 PM
Modified: 3/28/2024 1:51:17 PM

Electropherogram Summary Continued ...

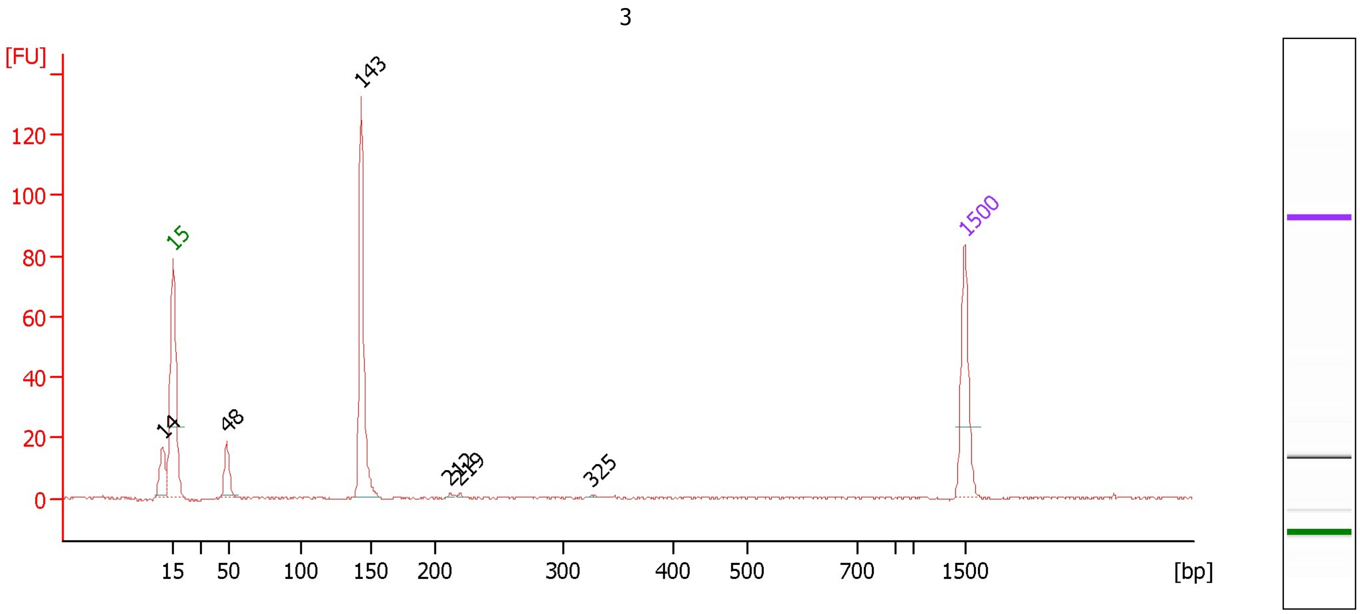

Overall Results for sample 3 : 3

Number of peaks found: 5

Peak table for sample 3 : 3

Peak		Size [bp]	Conc. [ng/µl]	Molarity [nmol/l]	Observations
1		14	0.00	0.0	
2	◀	15	4.20	424.2	Lower Marker
3		48	0.90	28.6	
4		143	4.58	48.6	
5		212	0.05	0.3	
6		219	0.04	0.3	
7		325	0.02	0.1	
8	▶	1,500	2.10	2.1	Upper Marker

 © Copyright 2003 - 2009 Agilent Technologies, Inc.

Assay Class: DNA 1000
Data Path: C:\...-28\2100 expert_DNA 1000_DE13804763_2024-03-28_13-17-54.xad

Created: 3/28/2024 1:17:53 PM
Modified: 3/28/2024 1:51:17 PM

Electropherogram Summary Continued ...

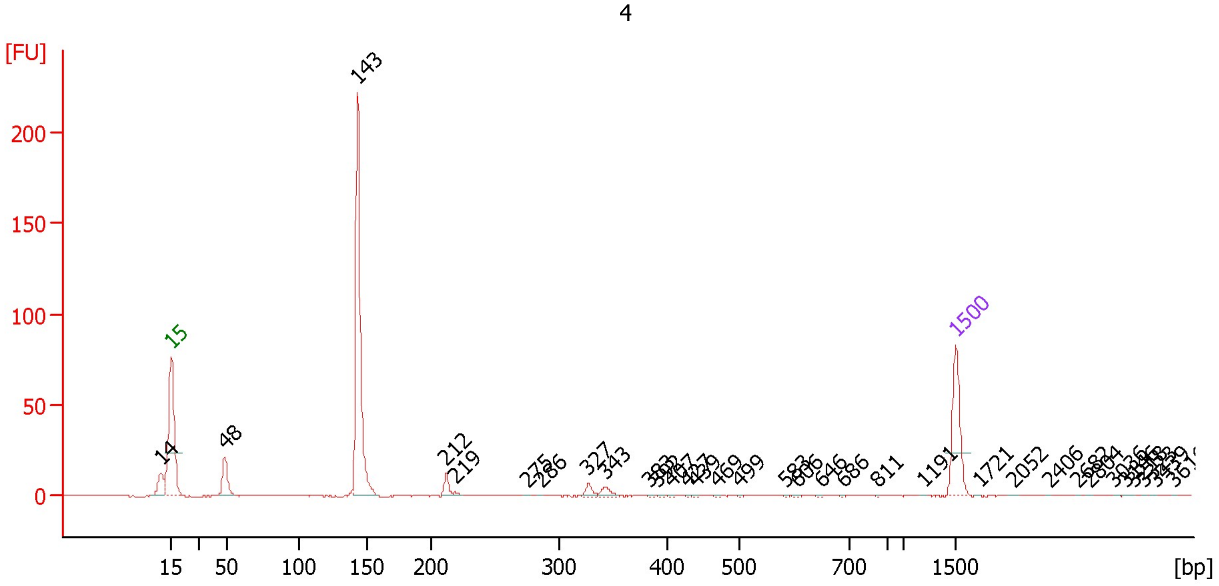

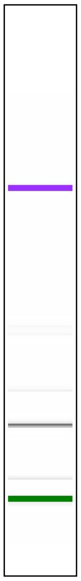

Overall Results for sample 4 : **4**

Number of peaks found: 21

Peak table for sample 4 : **4**

Peak		Size [bp]	Conc. [ng/µl]	Molarity [nmol/l]	Observations
1		14	0.00	0.0	
2	◀	15	4.20	424.2	Lower Marker
3		48	1.09	34.7	
4		143	7.75	82.4	
5		212	0.34	2.5	
6		219	0.05	0.4	
7		275	0.01	0.1	
8		286	0.01	0.1	
9		327	0.23	1.1	
10		343	0.22	1.0	
11		383	0.01	0.1	
12		392	0.01	0.1	
13		407	0.01	0.0	
14		427	0.01	0.0	
15		439	0.01	0.0	
16		469	0.01	0.0	
17		499	0.01	0.0	
18		583	0.01	0.0	
19		606	0.01	0.0	
20		646	0.01	0.0	
21		686	0.01	0.0	
22		811	0.01	0.0	
23		1,191	0.01	0.0	
24	▶	1,500	2.10	2.1	Upper Marker
25		1,721	0.00	0.0	
26		2,052	0.00	0.0	
27		2,406	0.00	0.0	

 © Copyright 2003 - 2009 Agilent Technologies, Inc.

Assay Class: DNA 1000
Data Path: C:\...-28\2100 expert_DNA 1000_DE13804763_2024-03-28_13-17-54.xad

Created: 3/28/2024 1:17:53 PM
Modified: 3/28/2024 1:51:17 PM

Electropherogram Summary Continued ...

... Peak table for sample 4 : 4

Peak	Size [bp]	Conc. [ng/µl]	Molarity [nmol/l]	Observations
28	2,682	0.00	0.0	
29	2,804	0.00	0.0	
30	3,036	0.00	0.0	
31	3,146	0.00	0.0	
32	3,218	0.00	0.0	
33	3,323	0.00	0.0	
34	3,439	0.00	0.0	
35	3,616	0.00	0.0	

© Copyright 2003 - 2009 Agilent Technologies, Inc.

Assay Class:	DNA 1000	Created:	3/28/2024 1:17:53 PM
Data Path:	C:\...-28\2100 expert_DNA 1000_DE13804763_2024-03-28_13-17-54.xad	Modified:	3/28/2024 1:51:17 PM

Electropherogram Summary Continued ...

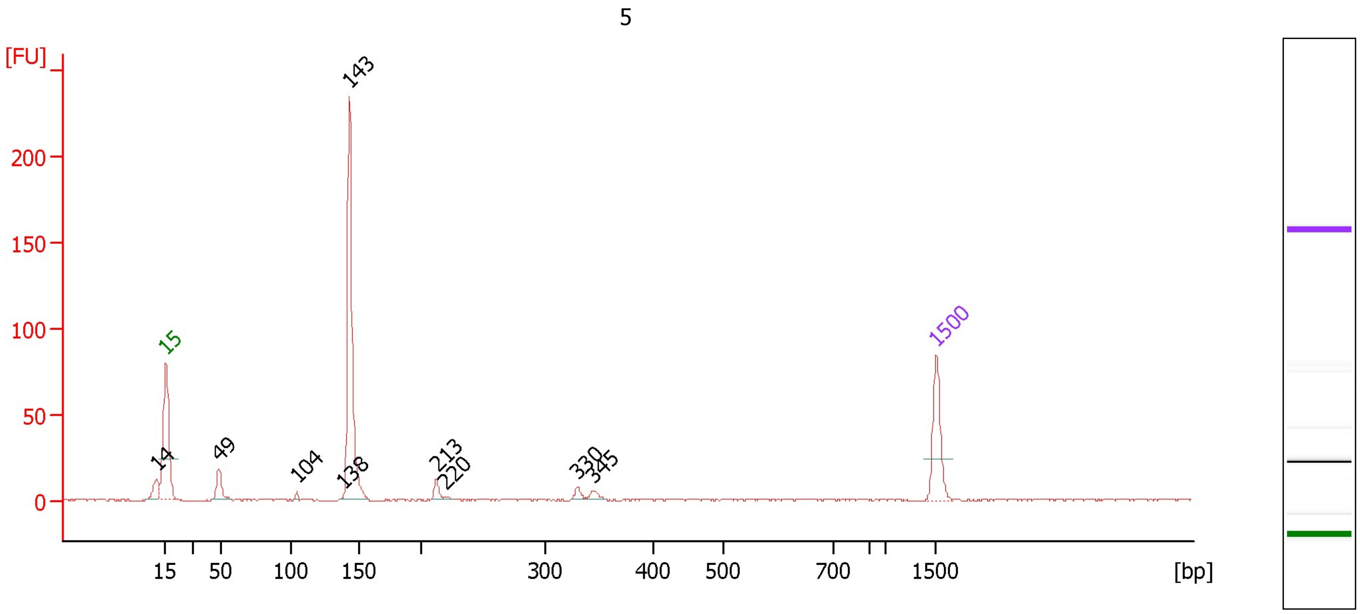

Overall Results for sample 5 : 5

Number of peaks found: 8

Peak table for sample 5 : 5

Peak		Size [bp]	Conc. [ng/µl]	Molarity [nmol/l]	Observations
1		14	0.00	0.0	
2	◀	15	4.20	424.2	Lower Marker
3		49	0.94	29.2	
4		104	0.08	1.1	
5		138	0.03	0.4	
6		143	8.22	86.8	
7		213	0.34	2.4	
8		220	0.06	0.4	
9		330	0.22	1.0	
10		345	0.23	1.0	
11	▶	1,500	2.10	2.1	Upper Marker

 © Copyright 2003 - 2009 Agilent Technologies, Inc.

Assay Class: DNA 1000
Data Path: C:\...-28\2100 expert_DNA 1000_DE13804763_2024-03-28_13-17-54.xad
Created: 3/28/2024 1:17:53 PM
Modified: 3/28/2024 1:51:17 PM

Electropherogram Summary Continued ...

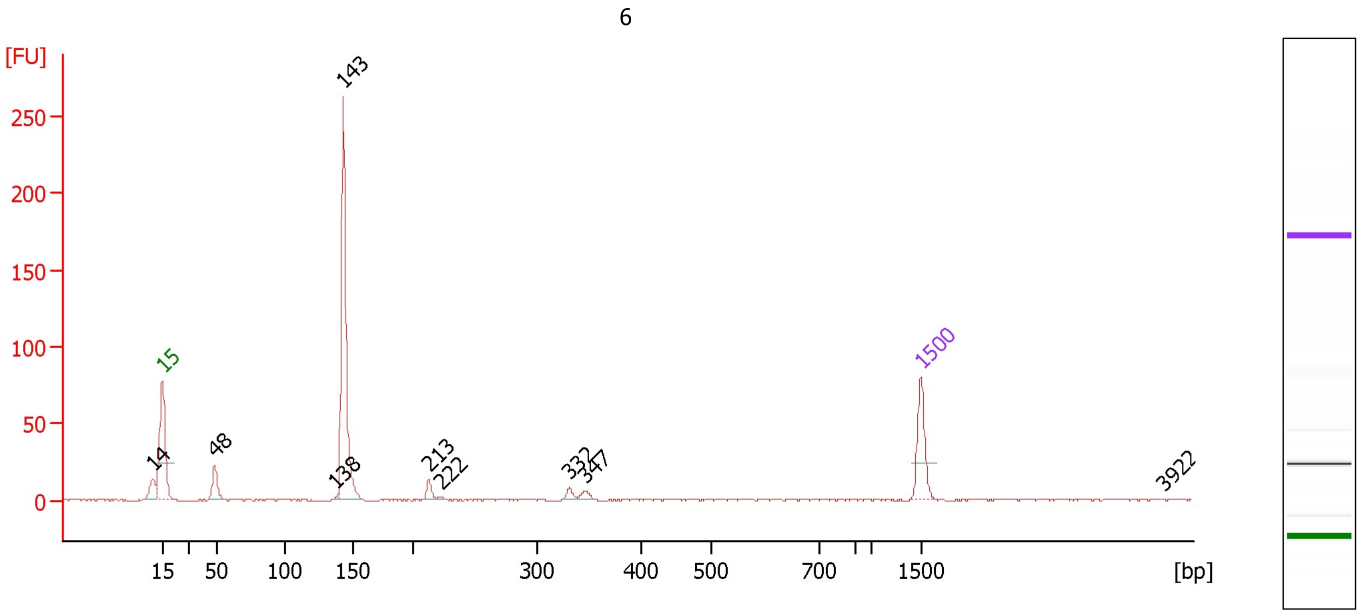

Overall Results for sample 6 : 6

Number of peaks found: 7

Peak table for sample 6 : 6

Peak		Size [bp]	Conc. [ng/µl]	Molarity [nmol/l]	Observations
1		14	0.00	0.0	
2	◀	15	4.20	424.2	Lower Marker
3		48	1.20	37.5	
4		138	0.05	0.5	
5		143	9.79	103.5	
6		213	0.41	2.9	
7		222	0.06	0.4	
8		332	0.25	1.2	
9		347	0.27	1.2	
10	▶	1,500	2.10	2.1	Upper Marker
11		3,922	0.00	0.0	

 © Copyright 2003 - 2009 Agilent Technologies, Inc.

Assay Class:	DNA 1000	Created:	3/28/2024 1:17:53 PM
Data Path:	C:\...-28\2100 expert_DNA 1000_DE13804763_2024-03-28_13-17-54.xad	Modified:	3/28/2024 1:51:17 PM

Gel Image

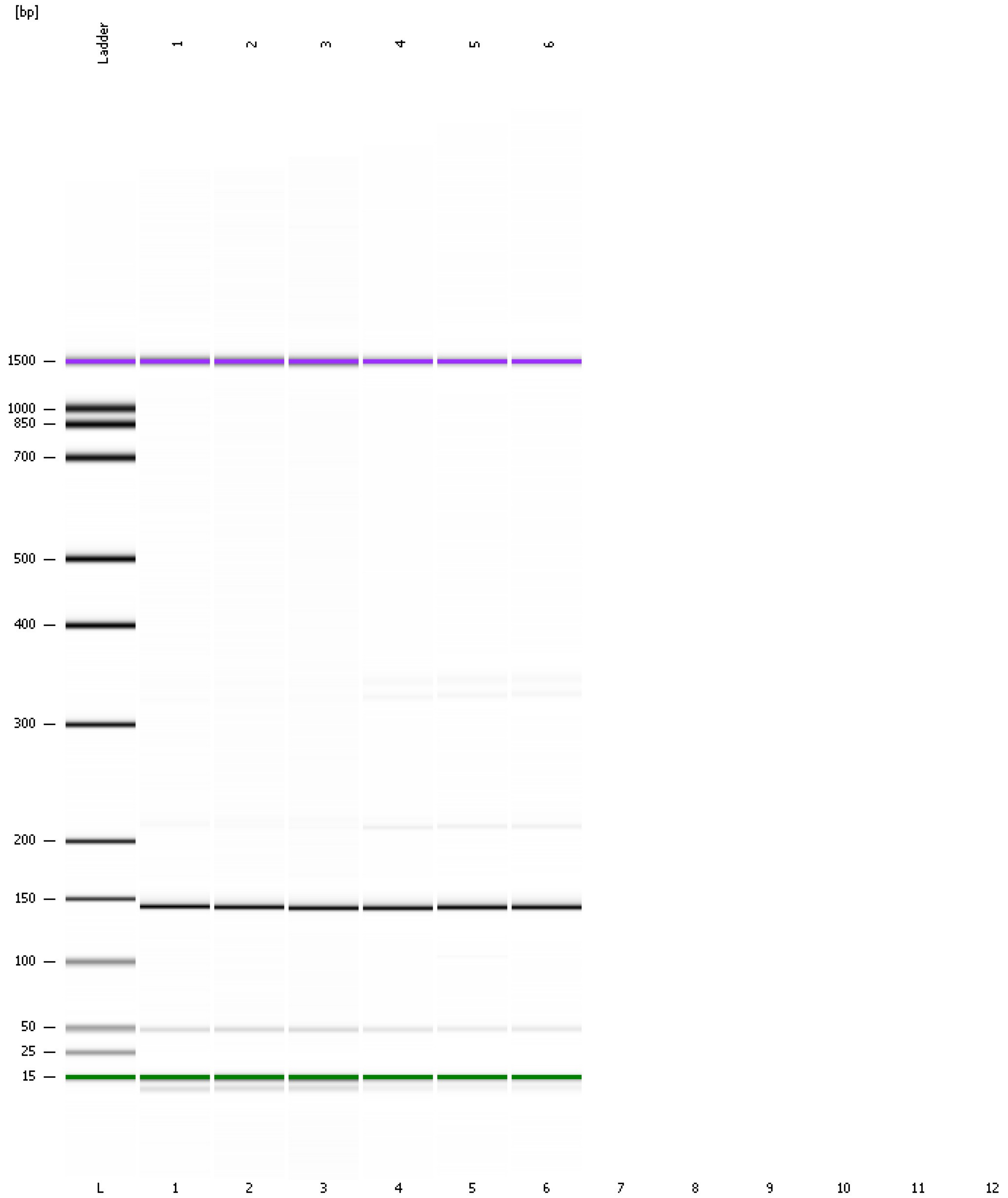

 © Copyright 2003 - 2009 Agilent Technologies, Inc.

Invalid Samples

Sample 7 has not been run, no results available.

Sample 8 has not been run, no results available.

Sample 9 has not been run, no results available.

Sample 10 has not been run, no results available.

Sample 11 has not been run, no results available.

Sample 12 has not been run, no results available.

 © Copyright 2003 - 2009 Agilent Technologies, Inc.
