# Supplementary material for: Competition for the conserved branch point sequence influences physiological outcomes in pre-mRNA splicing
Source: eLife. 2026 Mar 20;13:RP103167. doi: 10.7554/eLife.103167 (PMC13004596; doi:10.7554/eLife.103167)
Supplement: Figure 5—figure supplement 1—source data 1. [file elife-103167-fig5-figsupp1-data1.pdf]

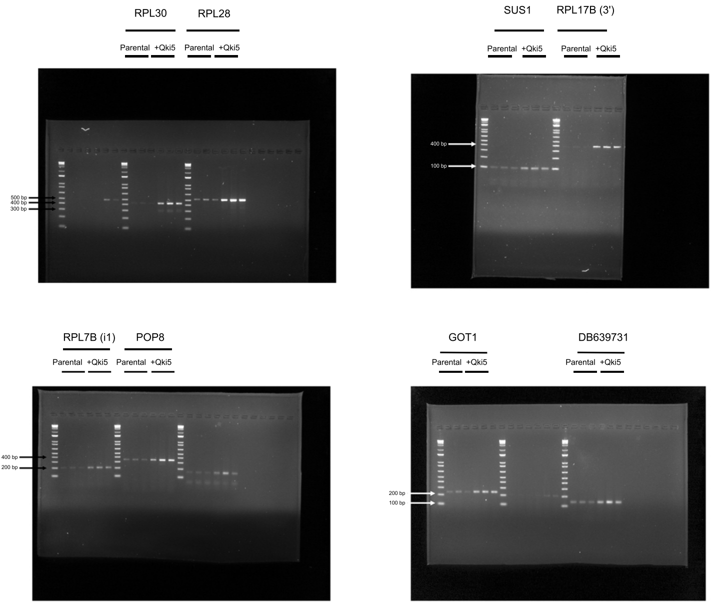

Supplementary Figure 5—source data 1. Original agarose gel images for Supplementary Figure 5. Original uncropped agarose gel images showing RT-PCR analysis of indicated gene transcripts in parental and +Qki5 cells. The panels display original gels for genes including RPL30, RPL28, SUS1, RPL17B (3'), RPL17 (i1), POP8, GOT1, and DB639731. The first lane of each gel corresponds to the molecular weight ladder with sizes indicated in base pairs (bp). For each gene, lanes correspond to biological triplicates of parental and +QKI5 yeast cells as depicted in the main manuscript. Only the genes labeled on the original gels were depicted in the final version of Supplementary Figure 5.

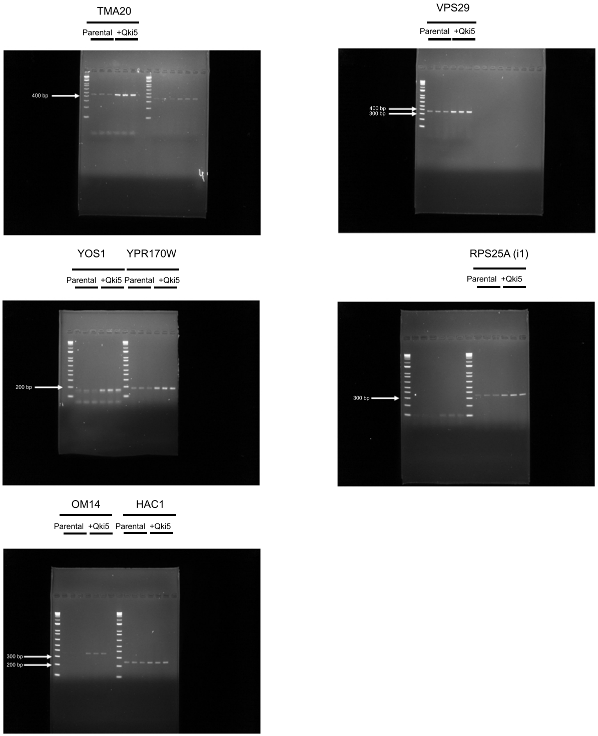

Supplementary Figure 5—source data 1. Original agarose gel images for Supplementary Figure 5, continued. Original uncropped agarose gel images showing RT-PCR analysis of the remaining gene transcripts in parental and +Qki5 cells. The panels display original gels for TMA20, VPS29, YOS160, YPR170W, RPS25A (i1), OM14, and HAC1. The first lane of each gel corresponds to the molecular weight ladder (bp). For each gene, lanes correspond to biological triplicates of parental and +Qki5 yeast cells as depicted in the main manuscript. Only the genes labeled on the original gels were depicted in the final version of Supplementary Figure 5.

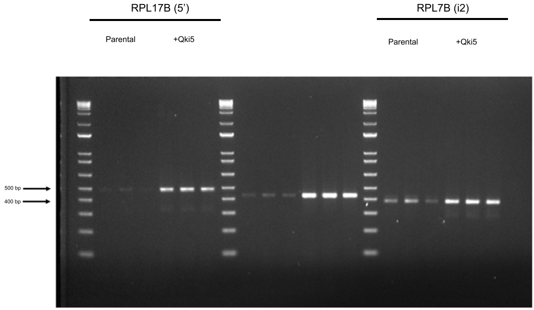

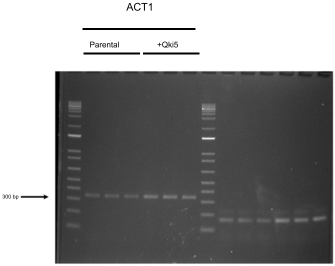

Supplementary Figure 5—source data 1. Original agarose gel images for Supplementary Figure 5, continued. Original uncropped agarose gel images showing RT-PCR analysis of the final set of gene transcripts in parental and +Qki5 cells. The panels display original gels for genes including RPL17B (5'), RPL7B (i2), and ACT1. The first lane of each gel corresponds to the molecular weight ladder with sizes indicated in base pairs (bp). For each gene, lanes correspond to biological triplicates of pa rental and +Qki5 yeast cells as depicted in the main manuscript. Only the genes labeled on the original gels were depicted in the final version of Supplementary Figure 5.
